# A Remarkable Selectivity Observed in Hetero-Diels–Alder Reactions of Levoglucosenone (LGO) with Thiochalcones: An Experimental and Computational Study †

**DOI:** 10.3390/molecules30183783

**Published:** 2025-09-17

**Authors:** Grzegorz Mlostoń, Katarzyna Urbaniak, Marcin Palusiak, Ernst-Ulrich Würthwein, Hans-Ulrich Reissig, Zbigniew J. Witczak

**Affiliations:** 1 Department of Organic and Applied Chemistry, Faculty of Chemistry, University of Lodz, Tamka 12, PL-91-403 Lodz, Poland; 2 Department of Physical Chemistry, Faculty of Chemistry, University of Lodz, Pomorska 163/165, PL-90-236 Lodz, Poland; marcin.palusiak@chemia.uni.lodz.pl; 3 Organisch-Chemisches Institut and Center for Multiscale Theory and Computation (CMTC), Universität Münster, Corrensstrasse 40, D-48149 Münster, Germany; 4 Institut für Chemie und Biochemie, Freie Universität Berlin, Takustrasse 3, D-14195 Berlin, Germany; hans.reissig@chemie.fu-berlin.de; 5 Department of Pharmaceutical Sciences, Nesbitt School of Pharmacy, Wilkes University, 84 W. South Street, Wilkes-Barre, PA 18766, USA; zbigniew.witczak@wilkes.edu

**Keywords:** hetero-Diels–Alder reactions, levoglucosenone, thiochalcones, sulfur heterocycles, single crystal X-ray analysis, DFT calculations

## Abstract

Levoglucosenone (LGO) smoothly undergoes microwave-assisted hetero-Diels–Alder reactions with thiochalcones in THF solution at 60 °C. The studied reactions are completed after 10 min, and the expected tricyclic 2,3-dihydro-4*H*-thiopyran derivatives are formed in a highly regio- and moderately stereoselective manner via competitive *exo*- and *endo*-attacks of the 1-thiadiene moiety onto the activated C=C bond of dienophile LGO. Although eight isomers are possible, only the formation of *exo*,*exo*- (major) and *exo*,*endo*- (minor) cycloadducts was observed. In most cases, isomeric products were separated by preparative layer chromatography and identified by means of spectroscopic methods. Some of the cycloadducts were obtained as single crystalline solids, and X-ray analyses enabled unambiguous confirmation of their structures. In order to explain the observed selectivity of the studied hetero-Diels–Alder reactions, DFT studies were carried out to determine the thermodynamic and kinetic properties of all regio- and stereoisomers. The results of these calculations predict the preferred formation of the two experimentally observed isomers. In addition, remarkable details on the electronic structure of *E*-1,3-diphenylprop-2-en-1-thione and on involved and hypothetical transition states could be elucidated.

## 1. Introduction

**Figure 1:**
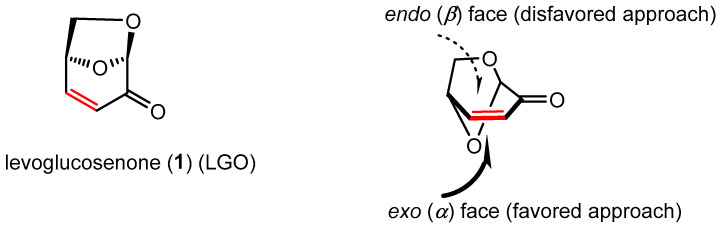
(−)-Levoglucosenone (LGO) (**1**) and presentation of two competitive approaches to the activated C=C bond.

(−)-Levoglucosenone (LGO) (**1**) (systematic names: *1,6-anhydro-3,4-dideoxy-β-D-glycero-hex-3-enopyranos-2-ulose* or *(1S,5R)-6,8-dioxabicyclo [3.2.1]oct-2-en-4-one*) [1,2] is considered a biomass-derived, chiral platform molecule that is accessible by a Brønsted acid-assisted pyrolysis of cellulose. Its first isolation and structure determination was published in 1979 by a Japanese group [3] (Figure 1), and the ‘Organic Syntheses’ procedure was recently elaborated by B. A. Greatrex et al., which allows the preparation of pure **1** (95% purity) on a laboratory scale in ca. 9% yield [4]. Due to the rapidly growing demand for large-scale applications, industrial methods for the fabrication of **1**, including biotechnological processes, have also been developed, and they are summarized in a recent review [5].

In recent years, a growing interest in new applications of **1** as a versatile reagent can be observed, and the compound has become an important enantiopure synthetic building block for the preparation of biologically active compounds available via multi-step procedures [6]. In addition, methods for the production of biodegradable polymers starting from levoglucosenone are currently under rapid development [7,8,9].

**Scheme 1:**
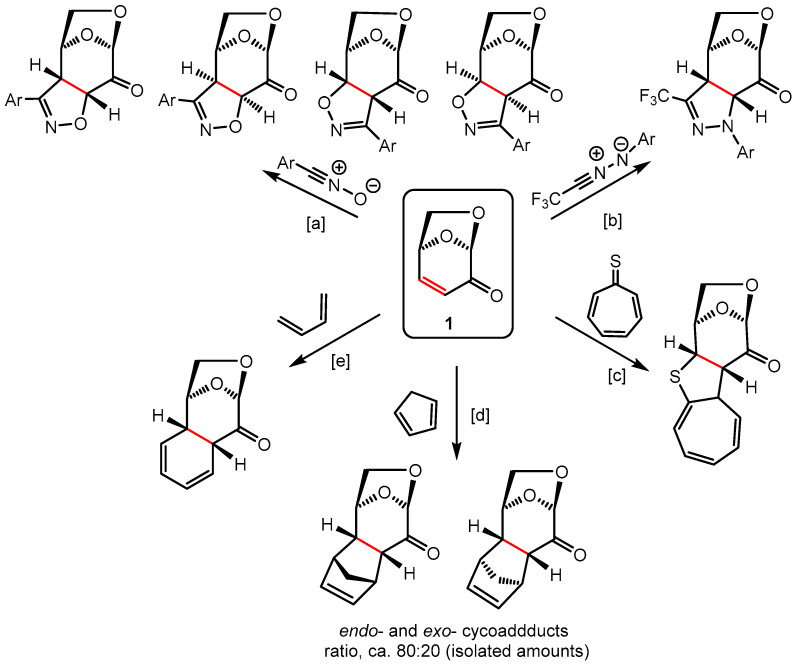
Selected examples of 1,3-dipolar cycloadditions, a higher order cycloaddition or Diels–Alder reactions starting with levoglucosenone (LGO) (**1**), and [a] nitrile oxides [10]; [b] fluorinated nitrile imines [12]; [c] tropothione [14]; [d] cyclopentadiene [15,16]; [e] buta-1,3-diene [15].

As shown in Scheme 1, efficient modifications of the LGO (**1**) skeleton can be achieved via various transformations of the reactive C=O and/or C=C bonds. In the current study, the α,β-unsaturated ketone moiety of **1** plays an important role in cycloadditions. A series of (3+2) cycloadditions (1,3-dipolar cycloadditions, Huisgen reactions) was successfully performed with **1** using nitrones [10,11], in situ-generated nitrile oxides [10], or nitrile imines [10,12] as 1,3-dipoles. Whereas reactions with a nitrile oxide and a *C*(Ph), *N*(Ph)-nitrile imine occurred with poor selectivity yielding mixtures of regio- and stereoisomeric isoxazole and pyrazole derivatives [10], (3+2) cycloadditions with fluorinated nitrile imines were highly stereoselective and *exo*-cycloadducts were formed as exclusive products without oxidation (and concomitant aromatization) of the initially formed 4,5-dihydropyrazole derivatives [12] (Scheme 1, [a] and [b]). The 1,3-dipolar cycloaddition of **1** with ethyl diazoacetate occurred with the formation of a single but unstable cycloadduct, which, under the reaction conditions, underwent further conversions initiated by dinitrogen elimination [13]. Notably, in a recent publication, a ‘higher order cycloaddition’ [(8+2) cycloaddition] of **1** with tropothione was also reported, and this reaction led to an *exo*-cycloadduct as a single product with complete stereoselectivity [14] (Scheme 1, [c]).

The first study on the (4+2) cycloadditions (Diels–Alder reactions) of LGO (**1**) with cyclopentadiene and buta-1,3-diene was published in 1981 [15]. In this report, the reaction with cyclopentadiene was claimed to occur with complete *exo*,*exo*-stereoselectivity with respect to both components (Scheme 1, [d] and [e]). Interestingly, the less reactive furan did not undergo the (4+2) cycloaddition with **1**, but in the presence of the Lewis acid AlCl_3_, a Michael adduct was isolated, with **1** acting as a Michael acceptor [15]. It is worth noting that in a subsequent publication, the reaction of **1** with cyclopentadiene was reported to deliver an approximate 80:20 mixture (according to isolated amounts of isomeric products) of *endo*- and *exo*-cycloadducts, and this result is also presented in Scheme 1 (equation [d]) [16]. In a recent publication, diverse compounds derived from **1** were obtained via manipulation of the initially obtained polycyclic Diels–Alder cycloadducts [17]. The (4+2) cycloadditions of LGO (**1**) and of 4-cyano-levoglucosenone with four differently substituted dienes—e.g., cyclopentadiene, 2,3-dimethylbuta-1,3-diene, etc.—were carefully studied by means of experimental and computational methods, and the influence of diene substituents on the regioselectivity of the observed reactions was discussed [18]. Moreover, asymmetric Diels–Alder reactions starting with **1** or its derivatives, applied as chiral auxiliaries, have also been described [19,20].

**Scheme 2:**
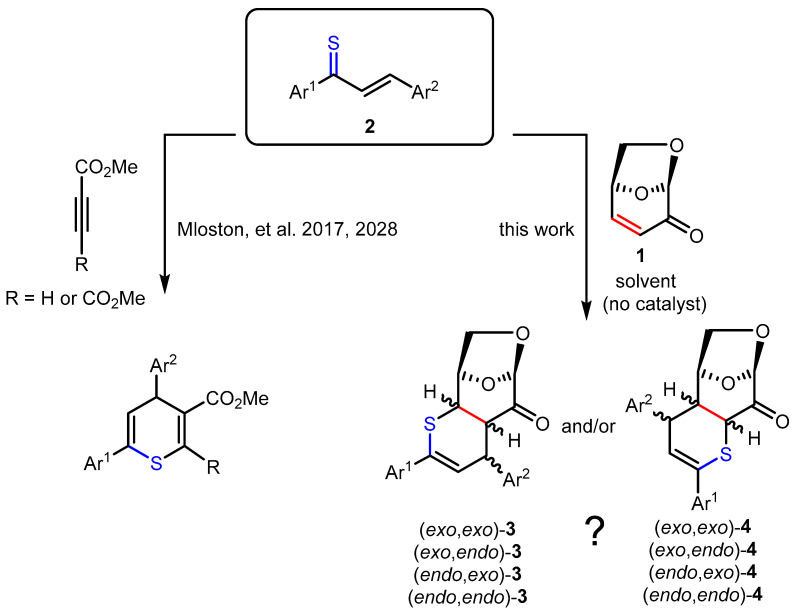
Hetero-Diels–Alder reactions of thiochalcones (**2**) with acetylenic dienophiles leading to functionalized thiopyrans [28,29] and the possible course of their reactions with levoglucosenone (**1**), providing stereoisomers of regioisomeric tricyclic products **3** or **4**.

Notably, in spite of the general importance of hetero-Diels–Alder reactions for the synthesis of various six-membered heterocycles [21,22,23], conversions of this type, starting with **1** as an active C=C dienophile, have not yet been reported. On the other side, in our continuing studies on cycloaddition reactions, sulfur-containing functionalized dipolarophiles play an important role. We employed aromatic and cycloaliphatic thioketones [24] as well as 1-thiadienes, such as thiochalcones **2** (α,β-unsaturated thioketones) [25,26,27], as reactive components. In the previous studies we found that thiochalcones efficiently capture acetylenic C≡C dienophiles to give functionalized 4*H*-thiopyrans in high yields (Scheme 2, left side) [28,29].

Encouraged by the well-documented importance of thiopyrans as attractive bio-isosters applicable in the design of some biologically active compounds and as practically useful building blocks for the preparation of more complex sulfur heterocycles [30], we decided to examine the usefulness of LGO (**1**) as a dienophile in hetero-Diels–Alder reactions with thiochalcones **2**. The main target of the present study was to prove whether novel, polycyclic, and enantiopure tricyclic thiopyrans can be formed in these reactions in the absence of any catalyst. In addition, the scope and limitations of this synthetic approach, as well as the configuration of the anticipated (4+2) cycloadducts **3**, should be established. Based on the results of already reported Diels–Alder reactions, formation of four diastereoisomers (*exo*,*exo*)-**3**, (*exo*,*endo*)-**3**, (*endo*,*exo*)-**3**, and (*endo*,*endo*)-**3** can be anticipated [15,16], whereas the formation of the regioisomeric cycloadducts **4** is less likely. In order to understand the experimental results and to support the suggested reaction mechanism of this thus far unexplored reaction, DFT calculations were performed. Importantly, discussions on mechanistic aspects of cycloaddition reactions (stepwise versus concerted pathways) are in the focus of attention of numerous research groups [31,32,33].

## 2. Results and Discussion

### 2.1. Experimental Work

**Scheme 3:**
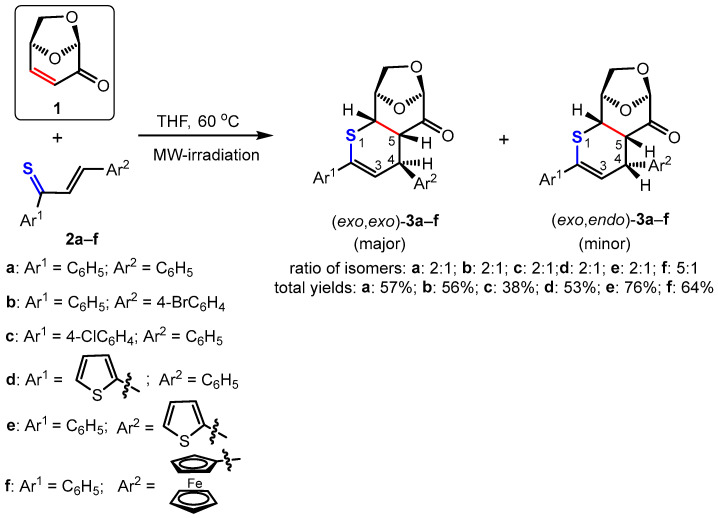
Regioselective hetero-Diels–Alder reactions of levoglucosenone (LGO) (**1**) with thiochalcones **2a**–**f** leading to the formation of isomeric *exo*,*exo*- and *exo*,*endo*-cycloadducts **3a**–**f** (tricyclic 3,4-dihydro-4*H*-thiopyrans).

The test experiment was performed in THF solution without any catalyst, starting with equimolar amounts of LGO (**1**) and thiochalcone **2a** (*E*-1,3-diphenylprop-2-en-1-thione). In the first experiment, the blue colored solution was heated in a thick-walled test tube with screw cap placed in an oil bath heated to 90 °C. After 1 h, the reaction was finished, but the brownish color of the obtained reaction solution indicated the formation of substantial amounts of tarry side products. Therefore, the reaction conditions were modified, and the second experiment was carried out in THF solution at 60 °C with the support of microwave (MW) irradiation. Gratifyingly, in this case, the reaction was complete already after 10 min, and the crude reaction mixture was almost colorless, indicating no formation of the undesired decomposition products. Based on this observation, the second protocol was applied as a standard procedure to study reactions of **1** with thiochalcones **2** (see Experimental). The ^1^H-NMR spectrum of the crude product mixture revealed two sets of signals, which could eventually be attributed to isomers of the expected cycloadduct derived from **1**. Two doublets found at 6.26 (^3^*J*_H,H_ = 6.20 Hz) and 6.61 (^3^*J*_H,H_ = 3.90 Hz) ppm were of particular diagnostic value as they were located in the characteristic region known for *H*C(3) atoms of the thiopyran skeleton (for numbering of atoms, see Scheme 3). The ratio of intensities of these signals was established to be ca. 2:1, and it thereby determined the ratio of the anticipated isomeric (4+2) cycloadducts **3a** formed in this reaction. Chromatographic separation (silica gel plate chromatographic separation) led to the isolation of two fractions, and the less polar fraction gave the major component found in the crude mixture in 40% yield. After crystallization, this fraction afforded colorless crystals with m.p. 148–149 °C. On the other hand, the more polar fraction separated chromatographically was identified as the minor component of the crude mixture (17% yield based on the isolated material). After crystallization, this cycloadduct formed colorless crystals with m.p. 186 °C (decomp.). The ^1^H-NMR spectra registered for purified products confirmed the structures of two isomers of the anticipated (4+2) cycloadducts. In the case of the major product, the most characteristic signals, found at 6.26 (d), 4.70 (m), and 4.37 (dd) ppm were attributed to *H*-atoms located at C(3), C(5), and C(4), respectively. For the minor product, analogous absorptions for the atoms *H*C(3), *H*C(5), and *H*(C4) were found at 6.61 (d), 4.75 (dd), and 4.33 (dd), respectively. In the ^13^C-NMR spectra of both products, the same number of signals (17 signals) confirmed the anticipated structure of the isomeric (4+2) cycloadducts, and the low-field shifted signals of the C=O group were found at 197.9 ppm for the major product and at 197.7 ppm for the minor component (Scheme 3).

Elemental analysis confirmed for both compounds the molecular formula C_21_H_18_O_3_S related to the structure of the isomeric (4+2) cycloadducts **3a**.

Similar results were obtained using thiochalcones **2b**–**f** as highly active 1-thiadienes. The ratios of the isomeric (*exo*,*exo*)- and (*exo*,*endo*)-cycloadducts **3b**–**e** were similar and in analogy to **3a**. The stereochemical structure is based on the registered ^1^H-NMR spectra of the crude product mixtures, giving ratios of approximately 2:1. An exceptional case was observed for ferrocenyl functionalized thiochalcone **3f**, which formed a 5:1 mixture of isomeric (*exo*,*exo*)- and (*exo*,*endo*)-cycloadducts. As expected, the ferrocenyl moiety exhibits a higher steric repulsion between LGO (**1**) and the sandwich-type ferrocenyl group, and therefore, the transition state leading to the (*exo*,*endo*)-**3f** is strongly disfavored (Scheme 3; Ar^2^ = F_c_). In analogy to **3a**, isomeric (*exo*,*exo*)- and (*exo*,*endo*)-cycloadducts **3b** and **3d** could successfully be separated by silica gel plate chromatographic separation (Scheme 3).

**Figure 2:**
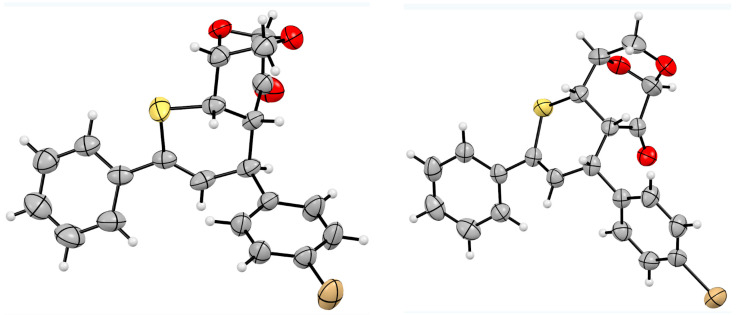
Molecular structures of the (4+2) cycloadducts (*exo*,*exo*)-**3b** (major, **left**) and (*exo*,*endo*)-**3b** (minor, **right**). Atoms are represented by thermal ellipsoids (50%). For graphics with atom labelling, see Figure S10a,b, respectively (Supplementary Materials).

In the case of cycloadducts **3b**, bearing a 4-BrC_6_H_4_ substituent at the *C*(4) atom, the separated isomers provided after crystallization from petroleum ether/CH_2_Cl_2_ mixture single crystals suitable for X-ray measurements which unambiguously confirmed the anticipated (*exo*,*exo*)-**3b** and (*exo*,*endo*)-**3b** structures for the less polar fraction (major isomer) and for the more polar fraction (minor isomer) (Figure 2).

Both isomeric cycloadducts **3b** crystallized in the same P 2_1_2,_1_2_1_ space group with a single molecule in the unit cell. The enantiomorphic space group confirms the presence of only one enantiomeric form in each X-ray diffraction-identified crystal phase. The absolute configuration of both compounds **3b** (*exo,exo* and *exo*,*endo*) is also confirmed by Flack parameter values collected in Table S1 (Supplementary Materials). The molecular geometry of both isomers is essentially comparable, with all bond lengths and angles falling within standard expected values. The key structural difference arises from the distinct arrangement of the 4-BrC_6_H_4_ substituent at the carbon atom C(4) in the heterocyclic ring (Figure 2).

**Scheme 4:**
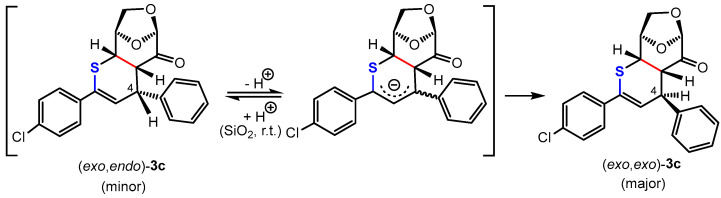
Epimerization process of less stable (*exo*,*endo*)-**3c** in the course of the attempted chromatographic purification (SiO_2_ column), leading to (*exo,exo*)-**3c**.

Remarkably, attempted chromatographic separation of isomeric cycloadducts **3c** with 4-ClC_6_H_4_ substituents at the carbon atom *C*(4) atom led to epimerization of the stereogenic center and the minor cycloadduct (*exo*,*endo*)-**3c**, which could initially be observed in the crude mixture of products, underwent complete conversion into the major one (i.e., (*exo*,*exo*)-**3c**). This effect can be rationalized by the electron-withdrawing effect of the 4-ClC_6_H_4_ substituent, which enhances the acidity of the *H*C(4) atom and thereby induces the observed epimerization by a deprotonation/protonation process (Scheme 4).

Notably, in the case of hetaryl-substituted cycloadducts **3e** and **3f**, all attempts to isolate pure diastereomers either by crystallization or by chromatography were unsuccessful. It is also worth stressing that no oxidation of compounds **3** was observed, which could lead to the corresponding 4*H*-thiopyran derivatives. In contrast, the structurally similar 2,3-dihydro-4*H*-thiopyrans, obtained by hetero-Diels–Alder reactions of quinones with thiochalcones, undergo this oxidation [34].

**Figure 3:**
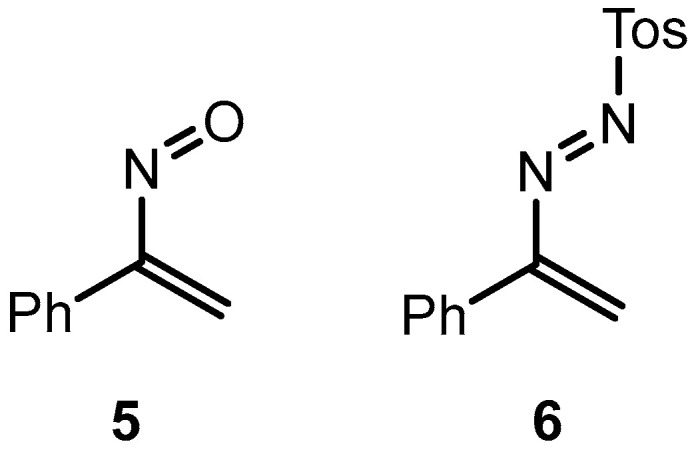
The in situ-generated electron-deficient heterodienes **5** and **6** examined in reactions with levoglucosenone (**1**).

In order to learn more about the scope and limitations of the presented hetero-Diels–Alder reactions of levoglucosenone (**1**), we compared the reactivity of rather electron-rich thiochalcones **2** with that of electron-deficient heterodienes such as in situ-generated α-nitrosoalkene **5** and azoalkene **6** (Figure 3), which react efficiently with thioketones, yielding the expected (4+2)-cycloadducts [35,36,37]. However, in both experiments performed under typical conditions [35,36], no formation of the anticipated six-membered cycloadducts—i.e., 1,2-oxazine or pyridazine derivatives, respectively—could be observed.

**Scheme 5:**
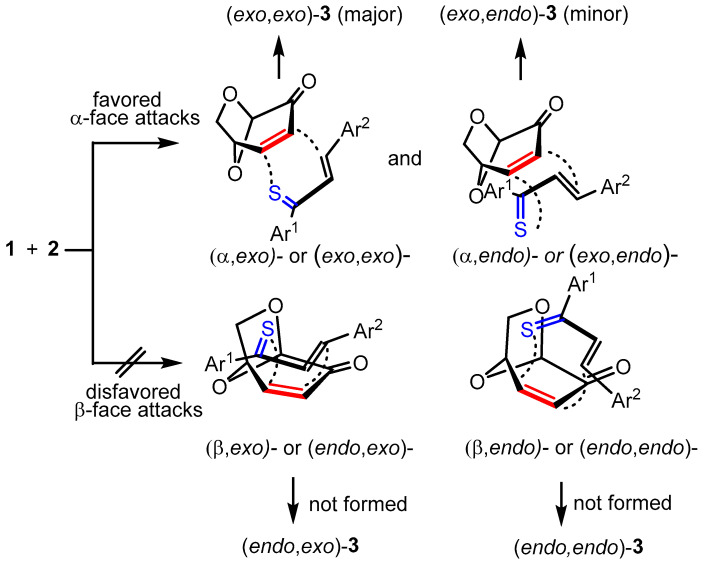
Presentation of four possible transition states in the concerted hetero-Diels–Alder reactions of **1** with thiochalcones **2,** leading to tricyclic thiopyrans **3**.

Before discussing the stereochemical course of the studied hetero-Diels–Alder reactions, the regioselectivity of the cycloaddition should be regarded. The performed experiments demonstrated that only products derived from an attack of the sulfur terminus of thiochalcones **2** on the β-carbon of the α,β-unsaturated ketone **1** are isolated. This observation is in accordance with our earlier study on the reactions of thiochalcones **2** with α-nitrosoalkenes; only cycloadducts resulting from an attack of the sulfur terminus of **2** to the more electrophilic β-carbon of the heterodiene were identified [25]. The formation of mixtures of the isomeric cycloadducts (*exo*,*exo*)-**3** and (*exo*,*endo*)-**3** is likely the diastereoselectivity of the cycloaddition, which is influenced by two factors controlling the corresponding transition states in the concerted (4+2) cycloadditions of starting dienophile **1** and heterodiene **2** (Scheme 5).

The obtained experimental results demonstrate that the exclusive reaction pathway via the favored attack of the less hindered α-face (*exo*-face) of **1** determines the structure of the finally formed (4+2) cycloadducts. The major products (*exo*,*exo*)-**3** result from *exo*-attacks with respect to thiochalcones **2,** and the minor products (*exo*,*endo*)-**3** from *endo*-approaches, respectively.

An alternative explanation of the observed formation of mixtures of isomeric cycloadducts **3** could be based on the assumption of a stepwise mechanism of the studied hetero-Diels–Alder reactions, either via intermediate zwitterionic or diradical species. In order to examine this hypothesis, an experiment was performed starting with **1** and thiochalcone **2a** (THF solution, MW irradiation) in the presence of 3 mol equivalents of methanol used as a trapping reagent (for a similar experiment reported by R. Sustmann et al., see e.g., [38]). The obtained mixture of crude products was examined by ^1^H-NMR spectroscopy, which demonstrated that its composition was identical to that discussed above, performed with **1** and **2a** in the absence of methanol. This result can be considered as strong evidence of a concerted and not a stepwise pathway of the hetero-Diels–Alder reactions of **1** with thiochalcones **2**. This assumption is supported by the comprehensive DFT calculations (vide infra).

### 2.2. Mechanistic Investigations by DFT Calculations

The mechanism as well as the regioselectivity and diastereoselectivity of the hetero-Diels–Alder reactions were also analyzed by a systematic quantum chemical DFT study of the eight possible isomers of **3a** and **4a** (see Scheme 2). In the following, the experimental results will be interpreted based on the calculated Gibbs free energy surfaces (ΔG_298_ [kcal/mol]), which describe the thermodynamic and kinetic properties.

On the basis of DFT optimizations using the B3LYP/6-31G(d) [39,40] + GD3BJ [41,42] functional, geometry optimizations using PBE1PBE/def2tzvp [43,44,45,46] + GD3BJ [42], including the PCM solvent sphere of tetrahydrofuran [47], were performed. Subsequent frequency calculations (zero-point vibrational energies and free enthalpy contributions) gave the final free Gibbs energies (DG298 [kcal/mol]), which were used for the interpretation of the experimental results. For all calculations, the GAUSSIAN 16 package of programs was used (see Supplementary Information) [48]. In accordance with the reaction conditions (60 °C, microwave irradiation, no light, no catalysts), only closed-shell calculations were performed. Some of the calculated minima were checked by the “stable” option of Gaussian; the wave functions were found to be stable under the perturbations considered.

**Scheme 6:**
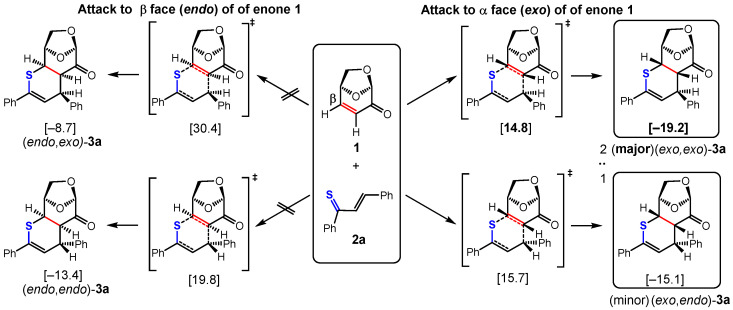
DFT results of the (4+2) cycloadditions of thiochalcone **2a** with levoglucosenone (LGO) (**1**) (PBE1PBE/def2tzvp + GD3BJ + PCM-tetrahydrofuran) (DG298 [kcal/mol]) leading to the four stereoisomeric tricyclic compounds **3a** (attack of the S-terminus of **2a** to the β-C atom of **1**). Symbol ^‡^: transition state.

**Scheme 7:**
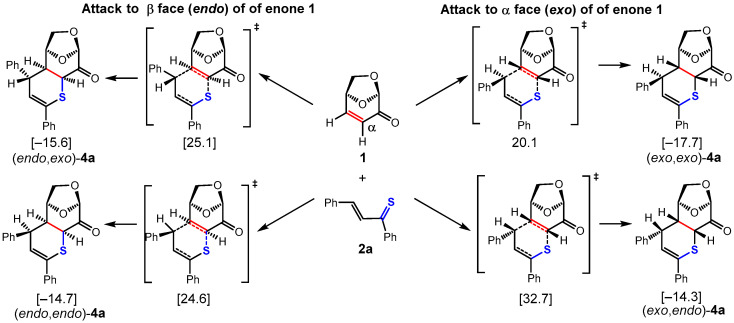
DFT results of the alternative (4+2)-cycloadditions of thiochalcone **2a** with levoglucosenone (**1**) (PBE1PBE/def2tzvp + GD3BJ + PCM-dichloromethane) (DG298 [kcal/mol]) leading to four stereoisomeric tricyclic compounds **4a** (attack of the S-terminus of **2a** to the α-carbon atom of **1**). Symbol ^‡^: transition state.

As shown in Scheme 6 and Scheme 7, thiochalcone **2a** (*E*-1,3-diphenylprop-2-en-1-thione) was employed as the standard hetero-diene for the calculations of the (4+2) cycloadditions with LGO (**1**) as used in the experiments. For the calculations of the reactions leading to **3a** and its hypothetical isomers, the configurations and conformations obtained from the X-ray structures of (*exo,exo*)-**3b** *(*Figure 2 left*)* and (*exo,endo*)-**3b** (Figure 2 right) were taken as starting structures for the complete optimizations. The transition states were localized by stepwise elongation of the respective C–C and C–S bonds (reaction path calculations), followed by transition state geometry optimization including frequency calculations.

Whereas the calculated NBO charges [49,50] of LGO **1** show the expected charge distribution of enones (O: −0.541 e^−^; C(O): 0.488 e^−^; C-α: −0.316 e^−^; C-β: −0.113 e^−^), the charge distribution of thiochalcone **2a** differs significantly due to the lower electronegativity of sulfur: S: −0.082 e^−^, C(S): −0.094 e^−^; C-α: −0.286 e^−^; C-β: −0.075 e^−^. Thus, the electrophilic and nucleophilic properties of the enone and of the thiochalcone are expected to be quite different, allowing unusual reactions as experimentally observed. Thus, the almost neutral sulfur atom of **2a** is able to attack at the quite nucleophilic α-atom as well as at the neighboring β-atom of the enone LGO (**1**). Scheme 6 and Scheme 7 show the eight possible pathways to cycloadducts **3a** and **4a** and the corresponding kinetic and thermodynamic data.

Scheme 6 presents the results of attacks of the almost neutral S terminus of **2a** to the β-carbon atom of enone **1** via the α-face (*exo*) and β-face (*endo*) (see Figure 1; compare refs. [28,29]). On the right-hand part of Scheme 6, the attacks to the *exo-*side of LGO (**1**) are given (O-bridge side, “from below”), on the left-hand part, the attacks to the *endo-*side (CH_2_-O-bridge side, “from above”) take place. In the upper line, the thiochalcone attacks with the β-C phenyl group in an *exo-*orientation, leading to (*exo,exo*)-**3a** or to (*endo,exo*)-**3a**. In the lower line of Scheme 6, this phenyl group is *endo-*positioned, giving (*exo,endo*)-**3a** and (*endo,endo*)-**3a**, respectively (see Scheme 5 for an alternative presentation).

As the calculations show, all four cycloadditions proceed exothermically (−19.2 to −8.7 kcal/mol). However, the calculated kinetic barriers (Gibbs free activation energies) are quite different, with **TS**-(*exo,exo*)-**3a** [14.8 kcal/mol] being the significantly lowest transition state calculated. Interestingly, this transition state leads to the experimentally observed major product (*exo,exo*)-**3a** (−19.2 kcal/mol), which is also the thermodynamically best isomer of all. Thus, this reaction is a good example of a kinetically controlled process leading to the experimentally observed major stereoisomer (*exo,exo*)-**3a**. The second best isomer, (*exo,endo*)-**3a** [−15.1 kcal/mol], is accessible over the second lowest barrier (15.7 kcal/mol); in fact, this compound was isolated as a minor product in the experiments (see above). The difference between (*exo,exo*)-**3a** and (*exo,endo*)-**3a** is clearly addressable to the sterically slightly more hindered position of the phenyl group at the β-C-position stemming from the thiochalcone. The hypothetical pathways leading to (*endo,exo*)-**3a** or (*endo,endo*)-**3a** have much higher Gibbs free activation energies (30.4 and 19.8 kcal/mol) due to the severe steric hindrance exhibited by the CH_2_-O-bridge of LGO (**1**). Hence, in accord with the experimental approaches of **2a** to this β-face of LGO (*endo*), they are unfavorable.

Similarly, as shown in Scheme 7, the calculated results from the attack of the S terminus of thiochalcone **2a** to the α-C of the LGO-enone show a thermodynamically and kinetically preferred attack to the α face (*exo*) of (LGO) **1**, giving (*exo,exo*)-**4a**. Interestingly, all four isomers of **4a** are thermodynamically in a narrow range (−14.3 to −17.7 kcal/mol), showing that steric interactions are similar for all products; in comparison, higher stability differences were calculated for isomer **3a**. However, the attack of the S terminus to the β-C of the enone to give (*exo,exo*)-**3a**, as described above, is kinetically preferred. For the hypothetical isomers **4a** attack to the β-face from the *endo*-side is again significantly disfavored compared to other stereochemical pathways. Although the mass balances of the experiments are only moderate to good (see Scheme 3), there is no experimental evidence for the formation of one of the **4a**-stereoisomers. From the kinetic point of view, no transition state shown in Scheme 7 is lower in energy compared to the data of Scheme 6. In general, the thermodynamically best isomers result from sulfur attacks towards the β-carbon atom of LGO (**1**) (see Scheme 6).

**Figure 4:**
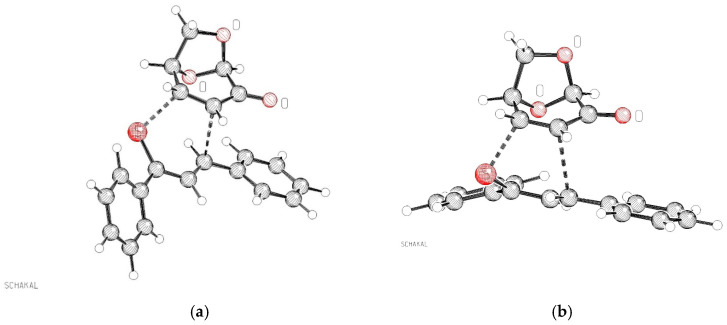
(**a**) **TS***-(exo,exo*)-**3a**, distance C⋯S: 2.289 Å, distance C⋯C: 2.608 Å. (**b**) **TS**-(*exo,endo*)-**3a**, distance C⋯S: 2.270 Å, distance C⋯C: 2.539 Å.

Both energy lowest transition states **TS**-(*exo,exo*)**-3a** and **TS**-(*exo,endo*)**-3a** (Figure 4a,b) show quite different atomic distances between the reacting C and S as well as C and C atoms [(a) **TS**-(*exo,exo*)-**3a**: C⋯S: 2.289 Å, C⋯C 2.608 Å, (b) **TS**-(*exo,endo*)-**3a**: C⋯S 2.270 Å, C⋯C 2.539 Å]. It is remarkable to see that the forming C⋯S bond is significantly shorter compared to the C⋯C bond, indicating a slightly asynchronous bond formation with moderate dominance of the C⋯S bond generation. Geometrical differences in the *exo,exo*- and *exo,endo-*transition states are only small, as illustrated in Figure 4 (**TS**-(*exo,exo*)-**3a**: C⋯S and C⋯C bonds are slightly larger than for **TS**-(*exo,endo*)-**3a**). As mentioned above, the low energetic difference of 0.9 kcal/mol between these two transition states results from the smaller steric interaction of the CH-phenyl group with the bicycle in **TS**-(*exo,exo*)-**3a**. The NBO-charges for **TS**-(*exo,exo*)-**3a** (LGO part: O: −0.586 e^−^; C(O): 0.457 e^−^; C-α: −0.350 e^−^; C-β: −0.179 e^−^; thiochalkone part: S: 0.093 e^−^; C(S): −0.136 e^−^; C-α: −0.275 e^−^; C-β: −0.060 e^−^) indicate a substantial charge transfer (0.279 e^−^) from the thiochalcogene to the LGO part of the transition state. For both forming bonds, the difference in charges of the reacting atoms is quite similar [C⋯S: 0.093 e^−^ − (−0.179 e^−^) = 0.272 e^−^ versus C⋯C: (−0.060 e^−^) − (−0.350 e^−^) = 0.290 e^−^].

**Figure 5:**
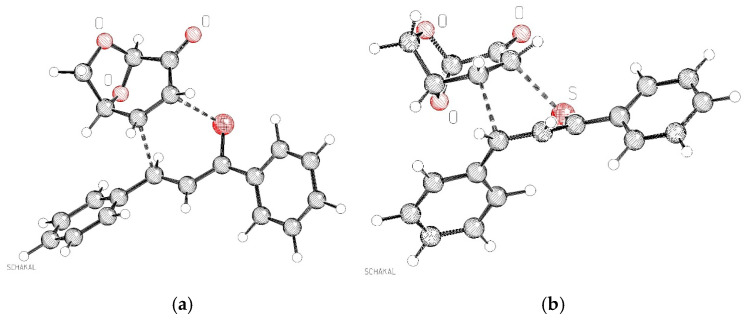
(**a**) **TS**-(*exo,exo*)-**4a**, distance C⋯S: 2.424 Å, distance C⋯C: 2.259 Å. (**b**) **TS**-(*exo,endo*)-**4a**, distance C⋯S: 2.394 Å, distance C⋯C: 2.342 Å.

Out of curiosity, the respective transitions **TS**-(*exo,exo*)-**4a** and **TS**-(*exo,endo*)-**4a** are depicted in Figure 5. In contrast to the **3a**-transition states they show shorter C⋯C distances compared to the S⋯C distances [(a) **TS**-(*exo,exo*)-**4a**, distance C⋯S: 2.424 Å, distance C⋯C: 2.259 Å; (b) **TS**-(*exo,endo*)-**4a**, distance C⋯S: 2.394 Å, distance C⋯C: 2.342 Å]. This can be interpreted as a preference for the bond formation to the β-carbon of enone **1**, regardless of an attack by a carbon or by a sulfur center of **2a**. The NBO charges of the relevant atoms for **TS**-(*exo,exo*)-**4a** (LGO part: O: −0.550 e^−^; C(O): 0.497 e^−^; C-α: −0.316 e^−^; C-β: −0.183 e^−^, thiochalkone part: S: 0.076 e^−^; C(S): −0.169 e^−^; C-α: −0.242 e^−^; C-β: −0.120 e^−^) differ significantly from those of **TS**-(*exo,exo*)-**3a**, being in sum by 0.106 e^−^ more positive. Remarkably, in the hypothetical **TS**-(*exo,exo*)-**4a,** the difference in charges of the reacting atoms is quite different (C⋯S: 0.391 e^−^ versus C⋯C: 0.062 e^−^) for the two forming bonds.

In summary, the DFT calculations reveal kinetic control of the studied hetero-Diels–Alder reactions and, in accord with the experimental results, the preferred attack of the sulfur atom of thiochalcone **2a** to the β-C of enone **1**. Not only is the correct regiochemistry proposed, but the observed diastereoselectivity with preferred attacks to the α-face (*exo*) leading to the thermodynamically best isomer (*exo,exo*)-**3a**) and to the second best isomer (*exo,endo*)-**3a**. The attack of the S-atom of **2a** to the α-C of LGO (**1**) is kinetically considerably disfavored, and in agreement with this result, isomers **4a** are experimentally not observed.

## 3. Materials and Methods

### 3.1. Materials

Starting materials*:* levoglucosenone (LGO) (**1**) was synthesized following the recently published procedure [4]; thiochalcones **2a**–**d** were prepared by treatment of the corresponding chalcones with Lawesson’s reagent as a thionating agent and purified by flash column chromatography according to the published procedures [25,51].

### 3.2. Analytical Methods and Equipment

General information*:* All commercially available solvents and reagents were used as received. If not stated otherwise, reactions were performed in flame-dried flasks under an argon atmosphere, and reactants were added by using a syringe; subsequent manipulations were conducted in the air. NMR spectra were taken with Bruker AVIII instruments [^1^H-NMR (600 MHz); ^13^C-NMR (151 MHz); Bruker, Billerica, MA, USA]. Chemical shifts are given relative to solvent residual peaks, integrals are in accordance with assignments, and coupling constants J are given in Hz. Elemental analyses were obtained with a Vario EL III instrument. Microwave experiments were carried out with CEM-focused Microwave-type (Yokohama-shi, Japan) Discover SPD at 150 W. Optical rotations were measured using an Anton Paar (Graz, Austria) MCP 500 polarimeter at the temperatures indicated. Melting points were determined in capillaries with an Aldrich (Saint Louis, MO, USA) Melt-Temp II apparatus and are uncorrected.

### 3.3. Quantum Chemical Calculations

Quantum Chemical calculations [PBE1PBE/def2-TZVP+PCM (tetrahydrofuran)+GD3BJ dispersion correction] [41,42,43,44,45,46,47] were performed on the basis of preceding B3LYP/6-31G(d) [39,40,41,42] +GD3BJ-geometry optimizations using the Gaussian 16, Revision B.01 [48], package of programs. Several conformers of each isomer were calculated, often after MM2-conformational analysis, to obtain the lowest energy isomer. The transition state localizations are based on reaction path calculations by elongation of both relevant bonds, starting with the cycloadducts (“retro-hetero-Diels–Alder reaction”) and full optimization of all other parameters. Transition state searches on the basis of the calculated 3D surfaces followed. In several cases, IRC calculations were performed in order to characterize the transition states obtained.

### 3.4. Synthesis

Reactions of thiochalcones **2a**–**d** with (−)-levoglucosenone (LGO) (**1**)-general procedure:


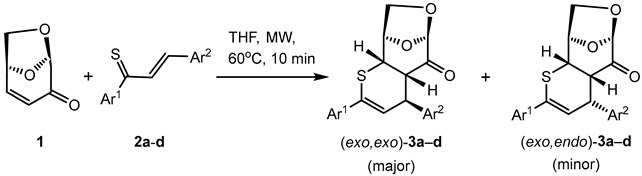


A solution of the corresponding thiochalcone **2** (1.1 mmol) and LGO (**1**) (126 mg, 1 mmol) in 4 mL of dry THF was irradiated in a microwave apparatus (200 W) at 60 °C for 10 min. Then, the reaction mixture was cooled down to room temperature, the solvent was evaporated, and the oily, brownish colored residue was preliminarily purified on a short chromatography column packed with silica gel (ca. 2–3 cm layer) using a CH_2_Cl_2_/petroleum ether (1:1) mixture as an eluent. The mixture of the crude products obtained thereafter was analyzed by ^1^H-NMR spectroscopy, which revealed the presence of two isomeric products. The repeated chromatography on a column (or on preparative plates coated with silica gel) allowed separation of isomeric products **3a**–**d**.

Attention*:* (1) Alternatively, a similar procedure was applied for experiments performed in THF solutions with reaction times estimated for 1h, and in this case, the reaction was carried out at 90 °C in a thick-walled, screwed test tube. Using this method, the formation of substantial amounts of decomposition products was observed, and the yields of isolated cycloadducts **3** were lower than in the case of the above-presented protocol with the MW support. (2) Syntheses of cycloadducts **3e** and **3f**: attempted separations of the two isomers failed, and the products were characterized as a mixture (their data are presented in the Supplementary Materials.


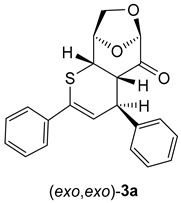


(4*R*,4a*S*,6*R*,9*R*,9a*S*)-2,4-Diphenyl-4a,8,9,9a-tetrahydro-4*H*-6,9-epoxythiopyrano [2,3-d]oxepin-5(6*H*)-one: *(exo,exo)-***3a** (major, isolated as less polar fraction). Yield: 140 mg (40%), colorless crystals, m.p. 148–149 °C (PE/CH_2_Cl_2_).

^1^H NMR (CDCl_3_): δ = 3.32 (*dd*, *J*_H,H_ = 6.1 Hz, *J*_H,H_ = 2.1 Hz, 1H); 3.83 (*dd*, *J*_H,H_ = 6.1 Hz, *J*_H,H_ = 2.0 Hz, 1H); 4.04–4.08 (*m*, 2H); 4.37 (*dd*, *J*_H,H_ = 6.1 Hz, *J*_H,H_ = 2.0 Hz, 1H); 4.70–4.71 (*m*, 1H); 5.32 (*s*, 1H); 6.26 (*d*, *J*_H,H_ = 6.2 Hz, 1H); 7.30–7.32 (*m*, 3*H*C_arom_); 7.35–7.39 (*m*, 5*H*C_arom_); 7.56–7.59 (*m*, 2*H*C_arom_).

^13^C NMR (CDCl_3_): δ = 37.9, 44.6, 46.5 (3H*C*); 67.2 (H_2_*C*-O); 75.9, 101.6 (2H*C*); 118.2, 126.9, 127.2, 128.1, 128.4, 128.5, 129.0 (10H*C*_arom_, and H*C*=); 134.1, 138.9, 142.7 (2*C*_arom_, Ph*C*=); 197.9 (C=O).

IR: ν 1741*s* (C=O), 1490*m*, 1448*m*, 1252*m*, 1110*s*, 1028*m*, 976*s*, 905*s*, 752*s*, 693*s*, cm^−1^.

EA for C_21_H_18_O_3_S (350.43): calcd. C 71.98, H 5.18, S 9.15; found C 71.78, H 5.15, S 9.37.

α = + 92.5 (CHCl_3_, c = 0.0020 g/mL).


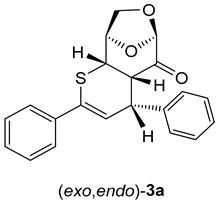


(4*S*,4a*S*,6*R*,9*R*,9a*S*)-2,4-Diphenyl-4a,8,9,9a-tetrahydro-4*H*-6,9-epoxythiopyrano [2,3-d]oxepin-5(6*H*)-one: (*exo*,*endo*)-**3a** (minor, more polar). Yield: 60 mg (17%), colorless crystals, m.p. 184–186 °C (diisopropyl ether/CH_2_Cl_2_).

^1^H NMR (CDCl_3_): δ = 3.62 (*pseudo t*, *J*_H,H_ = 3.3 Hz, 1*H*C); 3.81 (*dd*, *J*_H,H_ = 7.5 Hz, *J*_H,H_ = 2.8 Hz, 1*H*C); 4.10 (*dd*, *J*_H,H_ = 7.9 Hz, *J*_H,H_ = 5.6 Hz, 1*H*C); 4.21 (*d*, *J*_H,H_ = 7.9 Hz, 1*H*C); 4.33 (*dd*, *J*_H,H_ = 7.5 Hz, *J*_H,H_ = 1.6 Hz, 1*H*C); 4.75 (*dd*, *J*_H,H_ = 5.5 Hz, *J*_H,H_ = 1.5 Hz, 1*H*C); 5.12 (*s*, 1*H*C); 6.61 (*d*, *J*_H,H_ = 3.9 Hz, 1*H*C); 7.30–7.40 (*m*, 6H*C*_arom_); 7.53–7.58 (*m*, 4*H*C_arom_).

^13^C NMR (CDCl_3_): δ = 43.1, 46.7, 50.7 (3H*C*); 67.6 (H_2_*C*); 76.7, 102.5 (2H*C*); 123.5, 126.7, 126.8, 128.3, 128.4, 128.5, 128.8 (10H*C*_arom_, and H*C*=); 134.7, 138.8, 141.0 (2*C*_arom_, Ph*C*=); 197.7 (C=O).

IR: ν 1738*s* (C=O), 1491*m*, 1446*m*, 1300*m*, 1111*s*, 1029*m*, 972*s*, 916*s*, 749*s*, 693*s*, cm^−1^.

EA for C_21_H_18_O_3_S (350.43): calcd. C 71.98, H 5.18, S 9.15; found C 71.85, H 5.17, S 9.20.

α = + 31.1 (CHCl_3_, c = 0.0020 g/mL).


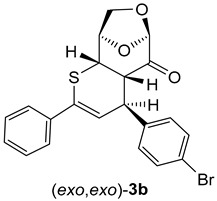


(4*R*,4a*S*,6*R*,9*R*,9a*S*)-(4-Bromophenyl)-2-phenyl-4a,8,9,9a-tetrahydro-4*H*-6,9-epoxythiopyrano [2,3-d]oxepin-5(6*H*)-one: (*exo*,*exo*)-**3a** (major, less polar). Yield: 170 mg (40%), yellowish crystals, m.p. 168–169 °C (petroleum ether/CH_2_Cl_2_).

^1^H NMR: δ = 3.26 (*dd*, *J*_H,H_ = 6.1 Hz, *J*_H,H_ = 2.1 Hz, 1H*C*); 3.77 (*dd*, *J*_H,H_ = 6.1 Hz, *J*_H,H_ = 2.1 Hz, 1H*C*); 4.06 (*d*, *J*_H,H_ = 2.8 Hz, 2*H*C); 4.32 (*dd*, *J*_H,H_ = 6.1 Hz, *J*_H,H_ = 2.1 Hz, 1*H*C); 4.69–4.71 (*m*, 1*H*C); 5.31 (*s*, 1*H*C); 6.20 (*d*, *J*_H,H_ = 6.2 Hz, 1*H*C=); 7.16, 7.49 (*AB-system*, ^3^*J*_H,H_ = 8.4 Hz, 4*H*C+_arom_); 7.32–7.38 (*m*, 3*H*C_arom_); 7.54–7.57 (*m*, 2*H*C_arom_).

^13^C NMR: δ = 37.5, 44.6, 46.4 (3H*C*); 66.9 (H_2_*C*-O); 75.7, 101.7 (2H*C*); 117.6, 126.5, 128.5, 129.8, 128.5 132.1 (9H*C*_arom_, and H*C*=); 121.1, 134.8, 138.7, 141.5 (3*C*_arom_, PhC=); 197.5 (C=O).

IR: ν 1744*s* (C=O); 1483*m*, 1435*m*, 1256*m*, 1103*s*, 1020*m*, 976*s*, 908*s*, 745*s*, 708*s*, 696*s*.

EA for C_21_H_17_BrO_3_S (429.33) calcd. C 58.75, H 3.99, S 7.47; found C 58.73, H 4.00, S 7.63.

α = +307.3 (CHCl_3_, c = 0.0018 g/mL).


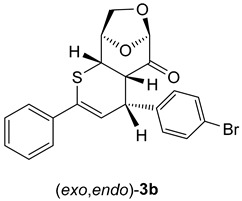


(4*S*,4a*S*,6*R*,9*R*,9a*S*)-2-Bromophenyl-4-phenyl-4a,8,9,9a-tetrahydro-4*H*-6,9-epoxythiopyrano [2,3-d]oxepin-5(6*H*)-one: (*exo*,*endo*)-**3b** (minor, more polar). Yield: 70 mg (16%), yellow crystals, m.p. 155–157 °C (petroleum ether/CH_2_Cl_2_).

^1^H NMR: δ = 3.57 (*t*, *J*_H,H_ = 3.4 Hz, 1*H*C); 3.75 (*dd*, *J*_H,H_ = 7.5 Hz, *J*_H,H_ = 2.8 Hz, 1*H*C); 4.09 (*dd*, *J*_H,H_ = 7.9 Hz, *J*_H,H_ = 5.1 Hz, 1*H*C); 4.19 (*d*, *J*_H,H_ = 7.9 Hz, 1*H*C); 4.31 (*dd*, *J*_H,H_ = 7.5 Hz, *J*_H,H_ = 1.6 Hz, 1*H*C); 4.74 (*dd*, *J*_H,H_ = 4.8 Hz, *J*_H,H_ = 1.2 Hz, 1*H*C); 5.11 (*s*, 1*H*C); 6.51 (*d*, ^3^*J*_H,H_ = 3.9 Hz, 1*H*C=); 7.32–7.37 (*m*, 3*H*C_arom_); 7.41, 7.49 (*AB-system*, *J*_H,H_ = 8.4 Hz, 4*H*C_arom_); 7.55–7.57 (2*H*C_arom_).

^13^C NMR: δ = 44.6, 46.6, 50.6 (3H*C*); 67.5 (H_2_*C*); 76.7, 102.4 (2H*C*); 122.6, 126.7, 127.7, 128.5, 130.6, 131.4 (9H*C*_arom_, H*C*=); 120.8, 135.2, 138.6, 141.0 (3*C*_arom_, and Ph*C*=); 197.6 (C=O).

IR: ν 1759*s* (C=O); 1483*m*, 1453*m*, 1300*m*, 1207*m*, 1000*s*, 942*s*, 905*s*, 752*s*, 695*s*, cm^−1^.

EA for C_21_H_17_BrO_3_S (429.33) calcd C 58.75, H 3.99, S 7.47; found C 58.75, H 3.97, S 7.54.

α = –7.8 (CHCl_3_, c = 0.0018 g/mL).


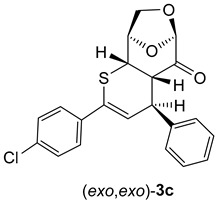


(4*R*,4a*S*,6*R*,9*R*,9a*S*)-2-(4-Chlorophenyl-4-phenyl-4a,8,9,9a-tetrahydro-4*H*-6,9-epoxythiopyrano [2,3-d]oxepin-5(6*H*)-one: (*exo*,*exo*)-**3c** (major, less polar). Yield: 145 mg (38%), yellowish crystals, m.p. 164–165 °C (petroleum ether/CH_2_Cl_2_).

^1^H NMR: δ = 3.26 (*dd*, *J*_H,H_ = 6.1 Hz, *J*_H,H_ = 2.3 Hz, 1*H*C); 3.76 (*dd*, *J*_H,H_ = 6.1 Hz, *J*_H,H_ = 1.9 Hz, 1*H*C); 4.06 (*d*, *J*_H,H_ = 2.9 Hz, *H*_2_C); 4.34 (*dd*, *J*_H,H_ = 6.2 Hz, *J*_H,H_ = 2.2 Hz, 1*H*C); 4.69–4.71 (*m,* 1*H*C); 5.30 (*s*, 1*H*C); 6.21 (*d*, *J*_H,H_ = 6.2 Hz, 1*H*C=); 7.22, 7.33 (*AB-*system, *J*_H,H_ = 8.8 Hz, 4*H*C_arom_); 7.35–7.39 (*m*, 3*H*C_arom_); 7.55–7.58 (*m*, 2*H*C_arom_).

^13^C NMR: δ = 37.4, 44.6, 46.3 (3H*C*); 67.0 (*C*H_2_-O); 75.7, 101.7 (2H*C*); 117.7, 126.4, 128.5, 128.7, 129.8, 132.0 (for 9H*C*_arom_, and H*C*=); 121.2, 134.9, 138.7, 141.5 (3*C*_arom_, Ph*C*=); 197.6 (C=O).

IR: ν 1744*s* (C=O); 1493*m*, 1448*m*, 1259*m*, 1107*s*, 1073*m*, 986*s*, 905*s*, 749*s*, 711*s*, 697*s*, cm^−1^.

EA for C_21_H_17_ClO_3_S (384.87) calcd C 65.54, H 4.45, S 8.33; found C 65.31, H 4.51, S 8.58.

α = + 117.0 (CHCl_3_, c = 0.0016 g/mL).


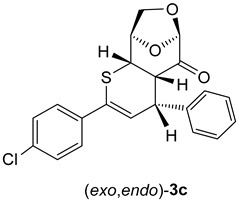


(4*S*,4a*S*,6*R*,9*R*,9a*S*)-2-(4-Chlorophenyl-4-phenyl-4a,8,9,9a-tetrahydro-4*H*-6,9-epoxythiopyrano [2,3-d]oxepin-5(6*H*)-one: (*exo*,*endo*)-**3b** (minor)—not isolated; underwent isomerization in the course of chromatographic purification on the SiO_2_ column.

Selected signals taken from the ^1^H-NMR spectrum of the crude mixture of isomeric products:

^1^H NMR: δ = 6.51 (*d*, 1H, *J*_H,H_ = 3.90 Hz), 5.11 (*s*, 1H), 4.73–4.75 (*m*, 1H).


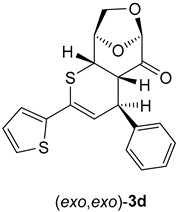


(4*R*,4a*S*,6*R*,9*R*,9a*S*)-4-Phenyl-2-(thiophen-2-yl)-4a,8,9,9a-tetrahydro-4*H*-6,9-epoxythiopyrano [2,3-d]oxepin-5(6*H*)-one: (*exo*,*exo*)-**3d** (major, less polar). Yield: 140 mg (39%), colorless crystals, m.p. 170 °C (diisopropyl ether/CH_2_Cl_2_).

^1^H NMR (CDCl_3_): δ **=** 3.33 (*dd*, *J*_H,H_ = 5.9 Hz, *J*_H,H_ = 2.2 Hz, 1*H*C); 3.83 (*dd*, *J*_H,H_ = 6.0 Hz, *J*_H,H_ = 2.0 Hz, 1*H*C); 4.04–4.09 (*m*, *H_2_*C); 4.34 (*dd*, *J*_H,H_ = 6.0 Hz, *J*_H,H_ = 2.2 Hz, 1*H*C); 4.71–4.72 (*m*, 1*H*C); 5.31 (*s*, 1*H*C); 6.34 (*d*, *J*_H,H_ = 6.0 Hz, 1*H*C=); 7.02 (*dd*, *J*_H,H_ = 5.0 Hz, *J*_H,H_ = 3.7 Hz, 1*H*C_arom_); 7.24–7.31 (*m*, 5*H*C_arom_); 7.36–7.38 (*m*, 2*H*C_arom_).

^13^C NMR: (CDCl_3_): δ **=** 37.9, 45.1, 47.1 (3H*C*); 67.0 (H_2_*C*); 75.6, 101.7 (2H*C*); 117.9, 124.1, 124.7, 127.3, 127.4, 128.1, 129.0 (for 8H*C*_arom_, and H*C*=); 127.7, 142.0, 142.7 (2*C*_arom_, and Ph*C*=); 197.7 (C=O).

IR: ν 1741*s* (C=O), 1599*m*, 1491*m*, 1449*m*, 1308*m*, 1256*m*, 1107*s*, 1017*m*, 976*s*, 916*s*, 898*m*, 771*m*, 697*s*, cm^−1^.

EA for C_19_H_16_O_3_S_2_ (356.45) calcd C 64.02, H 4.52, S 17.99; found C 63.89, H 4.60, S 17.89.

α = +72.4 (CHCl_3_, c = 0.0026 g/mL).


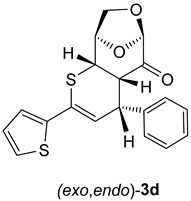


(4*R*,4a*S*,6*R*,9*R*,9a*S*)-4-Phenyl-2-(thiophen-2-yl)-4a,8,9,9a-tetrahydro-4*H*-6,9-epoxythiopyrano [2,3-d]oxepin-5(6*H*)-one: (*exo*,*endo*)-**3d** (minor, more polar). Yield: 50 mg (14%), beige crystals, m.p. 145 °C (diisopropyl ether/CH_2_Cl_2_).

^1^H NMR (CDCl_3_): δ **=** 3.66 (*pseudo t*, *J*_H,H_ = 3.4 Hz, 1*H*C); 3.80 (*dd*, *J*_H,H_ = 7.4 Hz, *J*_H,H_ = 2.8 Hz, 1*H*C); 4.09 (*dd*, *J*_H,H_ = 7.9 Hz, *J*_H,H_ = 5.0 Hz, 1*H*C); 4.20 (*d*, *J*_H,H_ = 7.9 Hz, 1*H*C); 4.35 (*dd*, *J*_H,H_ = 7.4 Hz, *J*_H,H_ = 1.7 Hz, 1*H*C); 4.73 (*dd*, *J*_H,H_ = 4.8 Hz, *J*_H,H_ = 1.2 Hz, 1*H*C); 5.12 (*s*, 1*H*C); 6.67 (*d*, *J*_H,H_ = 4.0 Hz, 1*H*C=); 7.00 (*dd*, *J*_H,H_ = 5.1 Hz, *J*_H,H_ = 3.6 Hz, 1*H*C_arom_); 7.21–7.27 (*m*, 3*H*C_arom_); 7.37–7.40 (*m*, 2*H*C_arom_); 7.51–7.52 (*m*, 2*H*C_arom_).

^13^C NMR (CDCl_3_): δ **=** 44.8, 46.7, 50.8 (3H*C*); 67.5 (H_2_*C*); 76.5, 102.4 (2H*C*); 122.2, 124.2, 124.8, 126.9, 127.3, 128.4, 128.9 (for 8H*C*_arom_, and H*C*=); 128.0, 140.6, 141.8 (2*C*_arom_, Ph*C*=); 197.5 (C=O).

IR: ν 1737*s* (C=O), 1589*m*, 1494*m*, 1450*m*, 1301*m*, 1226*m*, 1110*s*, 1025*m*, 969*s*, 902*s*, 864*m*, 752*m*, 693*s*, cm^−1^.

EA for C_19_H_16_O_3_S_2_ (356.45) calcd C 64.02, H 4.52, S 17.99; found C 64.05, H 4.15, S 17.57.

α = +36.4 (CHCl_3_, c = 0.0022 g/mL).

## 4. Conclusions

The presented study demonstrates for the first time that (−)-levoglucosenone (LGO) (**1**) can be successfully applied as a dienophile in the hetero-Diels–Alder reactions starting with thiochalcones **2** as easily available 1-thia-1,4-dienes. Thiopyran derivatives, which are formed as (4+2) cycloadducts, are known as practically useful pharmacophores applied for the preparation of biologically active compounds [52,53], which can act, for example, as enzyme inhibitors [54], antiproliferative [55], anti-inflammatory [56] or antibacterial [57] agents are well documented. Notably, even greater interest concerns biologically active derivatives of structurally similar thiochromane and thiochromene-based sulfur heterocycles [58]. Therefore, the presented method for the modification of levoglucosenone skeleton by annulation of the six-membered sulfur heterocycles can offer an attractive perspective for the search for its new, biologically active derivatives (see in [5,6]). Moreover, the presented method for the synthesis of 2,3-dihydro-4*H*-thiopyrans supplements the earlier described hetero-Diels–Alder reaction, which was based on the usage of typical 1,3-dienes and some thioaldehydes bearing a carboxamide group as reactive hetero-dienophile. In this study, the obtained (4+2) cycloadducts were evaluated for in vitro and ex vivo ACAT enzyme inhibition [59].

The studied hetero-Diels–Alder reaction occurred regioselectively and stereoselectively. Although eight isomers are possible, it led to only two diastereoisomeric products described as (*exo,exo*)- and (*exo,endo*)-cycloadducts, which differ by the orientation of the aryl substituent located at the newly created stereogenic center at *C*(4) atom of the thiopyran ring. Unexpectedly, the minor cycloadduct (*exo*,*endo*)-**3c** bearing the 4-ClC_6_H_4_ substituent at the *C*(4) atom, underwent slow epimerization in the course of chromatographic purification on the silica gel column. Remarkably, in contrast to electron-rich thiochalcones, electron-deficient heterodienes such as α-nitrosoethylenes and azoethylenes remain unreactive towards LGO (**1**).

The DFT calculations performed for the reactions of **1** with **2a** fully rationalize the experimentally observed selectivities and the preference for the formation of the sterically favored *exo*,*exo*-configured product **3a** as the major (4+2) cycloadduct. They also revealed a slightly asynchronous bond-forming scenario with shorter C⋯S distances in **TS**-*exo,exo*-**3a**, and **TS**-*exo,endo*-**3a** compared to the newly generated C⋯C bonds. Remarkably, the DFT calculations for the experimentally not observed regioisomers **4a** show the opposite effect. In these hypothetical reactions, the bond formations to the β-C atom of the enone system of LGO (**1**) are more advanced in the transition states than those to the α-C atom.

## Data Availability

Reported data are available from the authors via e-mail contact.

## Supplementary Materials

The following supporting information can be downloaded at: https://www.mdpi.com/article/10.3390/molecules30183783/s1. The Supplementary Materials contain some experimental details, the scanned ^1^H NMR and ^13^C NMR spectra of the described compounds, and information related to the single crystal X-ray measurements. The X-ray crystallography data of compounds (*exo*,*exo*)**-3b** and (*exo*,*endo*)-**3b** are deposited as CSD Communications under deposition numbers 2471272 and 2471271, respectively. The details of the DFT calculations, including the Gaussian archive entries, are also documented. Refs. [4,25,28,41,42,43,44,45,46,47,48,60,61,62,63,64,65] are cited in the Supplementary Materials.
