# Supplementary material for: A Remarkable Selectivity Observed in Hetero-Diels–Alder Reactions of Levoglucosenone (LGO) with Thiochalcones: An Experimental and Computational Study
Source: Molecules. 2025 Sep 17;30(18):3783. doi: 10.3390/molecules30183783 (PMC12472878; doi:10.3390/molecules30183783)
Supplement: Supplementary file 1 [file molecules-30-03783-s001.zip › molecules-3837022-supplementary.pdf]

A Remarkable Selectivity Observed in Hetero-Diels–Alder Reactions of Levoglucosenone (LGO) with Thiochalcones: An Experimental and Computational Study †

Grzegorz Mlostoń [1,*], Katarzyna Urbaniak [1], Marcin Palusiak [2], Ernst-Ulrich Würthwein [3,*], Hans-Ulrich Reissig [4] and Zbigniew J. Witczak [5]

[1] Department of Organic and Applied Chemistry, Faculty of Chemistry, University of Lodz, Tamka 12, PL-91-403 Lodz, Poland
[2] Department of Physical Chemistry, Faculty of Chemistry, University of Lodz, Pomorska 163/165, PL-90-236 Lodz, Poland; marcin.palusiak@chemia.uni.lodz.pl
[3] Organisch-Chemisches Institut and Center for Multiscale Theory and Computation (CMTC), Universität Münster, Corrensstrasse 40, D-48149 Münster, Germany
[4] Institut für Chemie und Biochemie, Freie Universität Berlin, Takustrasse 3, D-14195 Berlin, Germany; hans.reissig@chemie.fu-berlin.de
[5] Department of Pharmaceutical Sciences, Nesbitt School of Pharmacy, Wilkes University, 84 W. South Street, Wilkes-Barre, PA 18766, USA; zbigniew.witczak@wilkes.edu
* Correspondence: grzegorz.mloston@chemia.uni.lodz.pl (G.M.); wurthwe@uni-muenster.de (E.-U.W.); Tel.: +48-42-635-57-61 (G.M.)
† Dedicated to the memory of Professor Julian Chojnowski (Łódź) (1935–2025).

Contents:

Section 1: *General information* ..2
Section 2: *Starting materials* ..2
Section 3: *Reactions of levoglucosenone* (**1**) *with thiochalocnes* **2e**–**f**. ..2
Section 4: Copies of *^{1}H- and ^{13}C-NMR, and IR spectra of cycloadducts (exo,exo)-**3** and (exo,endo)-**3*** ..6
Section 5: *X-Ray structure determination of (exo,exo)-**3b*** and *(exo,endo)-**3b***, *Table 1* 19
Section 6: *DFT calculations* 22
Section 7: *References* 40

Section 1: *General information* (see also in Experimental)

1.1. *Reagents and solvents*: unless stated otherwise, used as commercially available with reagent grade and did not require further purification.

1.2. *NMR spectroscopy*: NMR spectra were recorded with a Bruker AVIII 600 (^{1}H NMR [600 MHz];^{13}C NMR [151 MHz]) or with a Varian Gemini 2000BB 200 MHz (^{19}F NMR [188 MHz]) instrument. Chemical shifts are reported relative to solvent residual peaks (^{1}H NMR, δ = 7.25 ppm [$CDCl_3$]; ^{13}C NMR, δ = 77.0 ppm [$CDCl_3$]).

1.3. *Optical rotations*: determined with an Anton Paar MCP 500 polarimeter at the temperatures indicated.

1.4. *Flash chromatography*: products were purified by flash column chromatography (CC) on silica gel (230–400 mesh, Merck).

1.5. *Preparative thin-layer chromatography (PLC)* was carried out using 20x20 cm glass plates coated with silica (60 PF_{254}, Merck). In all cases, separation of products was achieved using dichloromethane/ethanol (98:2) mixture for elution.

1.6. *Melting points* were determined in capillaries with a MEL-TEMP apparatus (Aldrich) and are uncorrected.

1.7. *Elemental analyses* were obtained with a Vario EL III (Elementar Analysensysteme GmbH) instrument.

1.8. *Preparation of starting materials and general procedures*: see also in Experimental in the main manuscript.

Section 2*: Starting materials*

Levoglucosenone (**1**) was prepared by pyrolysis of cellulose in polyethylene glycol, in the presence of H_2SO_4 according to the recently published procedure [1]. Thiochalcones **2e** [2] and **2f** [3] were obtained by heating of the corresponding chalcones with Lawesson's reagent in boiling THF.

Section 3: *Reactions of levoglucosenone* (**1**) *with thiochalocnes* **2e**–**f**

***Reactions of levoglucosenone (1) with thiochalcones 2e-f* – general procedure:** A solution of corresponding thiochalcone **2** (1.1 mmol) and levoglucosenone (LGO) (**1**) (126 mg, 1 mmol) in 4 mL of dry THF was irradiated in a MW apparatus (200 Watt) at 90 °C for 10 min.. After this time the reaction solution was cooled down to room temperature, the solvent was evaporated and the oily, brownish colored residue was preliminarily purified on a short

chromatographic column packed with silica gel (ca. 2-3 cm layer) using CH_2Cl_2/petroleum ether (1:1) mixture as an eluent. The crude products **3** obtained thereafter were analyzed by ^{1}H-NMR spectroscopy which revealed the presence of two isomeric products in different ratios (see Scheme 3 main manuscript).

The repeated chromatography on preparative plates coated with silica gel did not allow separation of the pure (*exo*,*exo*)-**3e** and (*exo*,*endo*)-**3e** as well as (*exo*,*exo*)-**3f** and (*exo*,*endo*)-**3f**. Attempted fractional crystallization of the cycloadducts was also unsuccessful. Therefore, they were spectroscopically characterized by selection of signals taken from the registered spectra of analytically pure mixtures.

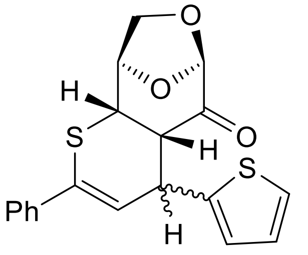 (*exo,exo*)- and (*exo,endo*)-**3e** mixuture of isomers	(4*R*,4a*S*,6*R*,9*R*,9a*S*)-2-Phenyl-4-(thiophen-2-yl)-4a,8,9,9a-tetrahydro-4*H*-6,9-epoxythiopyrano[2,3-d]oxepin-5(6*H*)-one: (*exo*,*exo*)-**3e** and (4*R*,4a*S*,6*R*,9*R*,9a*S*)-2-Phenyl-4-(thiophen-2-yl)-4a,8,9,9a-tetrahydro-4*H*-6,9-epoxythiopyrano[2,3-d]oxepin-5(6*H*)-one: (*exo*,*endo*)-**3e**, ca. 2:1 ratio. Yield: 270 mg (76 %), yellowish, viscous oil.

Cycloadduct (*exo*,*exo*)**-3e** (major):

^{1}H NMR ($CDCl_3$): δ = 3.43 (*dd*, $J_{H,H}$ = 6.1 Hz, $J_{H,H}$ = 2.2 Hz, 1*H*C); 3.95 (*dd*, $J_{H,H}$ = 6.0 Hz, $J_{H,H}$ = 2.8 Hz, 1*H*C); 4.09 (*dd*, $J_{H,H}$ = 9.3 Hz, $J_{H,H}$ = 5.0 Hz, 1*H*C); 4.15 (*d*, $J_{H,H}$ = 7.9 Hz, 1*H*C); 4.62 (*dd*, $J_{H,H}$ = 6.3 Hz, $J_{H,H}$ = 2.9 Hz, 1*H*C); 4.72 (*dd*, $J_{H,H}$ = 4.7 Hz, $J_{H,H}$ = 1.3 Hz, 1*H*C); 5.31 (*s*, 1*H*C); 6.29 (*d*, $J_{H,H}$ = 6.4 Hz, 1*H*C=); 6.95 (*d*, $J_{H,H}$ = 3.8 Hz, *H*C); 6.99–7.57 (*m*, 8*H*C_{arom}).

Cycloadduct (*exo,endo*)**-3e** (minor): δ = 3.82 (*dd*, $J_{H,H}$ = 7.8 Hz, $J_{H,H}$ = 2.6 Hz, 1*H*C); 3.84 (*pseudo t*, $J_{H,H}$ = 3.0 Hz, 1*H*C); 4.09 (*dd*, $J_{H,H}$ = 9.3 Hz, $J_{H,H}$ = 5.0 Hz, 1*H*C); 4.19 (*d*, $J_{H,H}$ = 7.9 Hz, 1*H*C); 4.30 (*dd*, $J_{H,H}$ = 7.8 Hz, $J_{H,H}$ = 1.5 Hz, *1HC*); 4.75 (*d*, $J_{H,H}$ = 5.2 Hz, 1*H*C); 5.15 (*s*, 1*H*C); 6.54 (*d*, $J_{H,H}$ = 4.0 Hz, 1*H*C=); 6.95 (*d*, $J_{H,H}$ = 3.8 Hz, 1*H*C_{arom}); 6.99–7.57 (*m*, 8*H*C_{arom}).

^{13}C NMR ($CDCl_3$), (mixture of isomers): δ = 37.8, 44.9, 45.1, 46.7, 47.2, 50.7 (all H*C*); 66.9, 67.5 (2 H_2*C*); 75.5, 76.5, 101.5, 102.5 (all H*C*); 117.9, 122.4, 124.3, 124.5, 124.9, 124.9, 127.6,

127.0, 127.5, 127.7, 128.3, 128.5, 129.0, 129.2 (for 12 HC_{arom}, and 2 H*C*=); 127.6, 128.6, 1407, 141.9, 142.2, 142.9 (for 4 C_{arom}, and 2 Ph*C*=); 197.3, 197.8 (for 2 C=O).

IR (mixture): ν 1741*s* (C=O), 1490*m*, 1450*m*, 1442*m*, 1305*m*, 1252*m*, 1107*s*, 1029*m*, 976*s*, 905*s*, 884*m*, 753*m*, 697*s*, cm^{-1}.

EA (mixture) for $C_{19}H_{16}O_3S_2$ (356.45): calcd. C 64.02, H 4.52, S 17.99; found C 63.96, H 4.43, S 17.94.

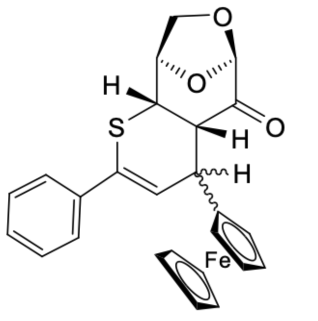<br>(*exo*,*exo*)- and (*exo*,*endo*)-**3f** mixture of isomers	(4*R*,4a*S*,6*R*,9*R*,9a*S*)-4-Ferrocenyl-2-phenyl-4a,8,9,9a-tetrahydro-4*H*-6,9-epoxythiopyrano[2,3-d]oxepin-5(6*H*)-one: (*exo*,*exo*)-**3f** and (4*S*,4a*S*,6*R*,9*R*,9a*S*)-4-Ferrocenyl-2-phenyl-4a,8,9,9a-tetrahydro-4*H*-6,9-epoxythiopyrano[2,3-d]oxepin-5(6*H*)-one: (*exo*,*endo*)-**3f**, ca. 5:1 ratio. Yield: 295 mg (64%), brownish-orange crystals, m.p. >158 °C (decomp.) (petroleum ether/CH_2Cl_2).

Cycloadduct (*exo*,*exo*)-**3f** (major) (based on the registered spectrum of the purified mixture): ^{1}H NMR ($CDCl_3$): δ = 3.21 (*dd*, $J_{H,H}$ = 6.2 Hz, $J_{H,H}$ = 2.3 Hz, 1*H*C); 3.76 (*dd*, $J_{H,H}$ = 6.2 Hz, $J_{H,H}$ = 1.8 Hz, 1*H*C); 4.04–4.18 (*m*, 4*H*C and 3*H*C_{Fc}); 4.22 (*s*, 5*H*C_{Fc}); 4.65–4.67 (*m*, 1*H*C_{Fc}); 5.28 (*s*, 1*H*C); 6.44 (*d*, $J_{H,H}$ = 6.2 Hz, 1*H*C=); 7.38–7.41 (*m*, 3CH_{arom}); 7.57–7.59 (*m*, 2*H*C_{arom}).

^{13}C NMR: δ = 32.5, 44.9, 47.3 (3H*C*); 66.6, 67.3, 68.1, 68.3 (4HC_{Fc}), 68.7 (5HC_{Fc}); 67.0 (H_2C); 75.7 (1H*C*), 89.8 (C_{Fc}); 101.7 (1H*C*); 118.9, 126.5, 128.4, 128.5 (for 5HC_{arom}, and H*C*=); 132.7, 139.2 (1C_{arom}, and Ph*C*=); 198.1 (C=O).

Cycloadduct (*exo*,*endo*)-**3f** (minor) (based on the registered spectrum of unseparated mixture): ^{1}H NMR ($CDCl_3$): δ = 3.55 (*pseudo t*, $J_{H,H}$ = 3.4 Hz, 1*H*C); 3.47 (*dd*, $J_{H,H}$ = 7.7 Hz, $J_{H,H}$ = 2.8 Hz, 1*H*C); 4.04–4.18 (*m*, 4*H*C, and 3*H*C_{Fc}); 4.20 (*s*, 5*H*C_{Fc}); 4.70 (*brd*, $J_{H,H}$ = 5.2 Hz, 1*H*C_{Fc}); 5.07 (*s*, 1*H*C); 6.63 (*d*, $J_{H,H}$ = 3.9 Hz, 1*H*C=); 7.34–7.37 (*m*, 3*H*C_{arom}); 7.59–7.60 (*m*, 2*H*C_{arom}).

^{13}C NMR ($CDCl_3$): δ = 40.3, 48.1, 50.5 (3H*C*); 67.4, 68.0, 68.5, 69.6 (4HC_{Fc}), 68.6 (5*H*C_{Fc}); 67.5 (H_2C); 76.8 (1H*C*), 88.7 (C_{Fc}); 102.4 (1H*C*); 124.6, 128.3, 128.6, 128.7 (5HC_{arom}, and H*C*=); 133.6, 138.9 (1C_{arom}, and Ph*C*=); 197.6 (C=O).

IR (mixture): ν 1737*s* (C=O); 1490*m*, 1448*m*, 1315*m*, 1252*m*, 1110*s*, 1022*m*, 1002*m*, 992*s*, 917*s*, 857*m*, 769*s*, 719*s*, cm^{-1}.

EA for $C_{25}H_{22}FeO_3S$ (458.35) (mixture): calcd C 65.51, H 4.84, S 6.99; found C 65.42, H 4.93, S 7.21.

Section 4: Copies of *^{1}H-, ^{13}C-NMR, and IR-spectra of cycloadducts **3***

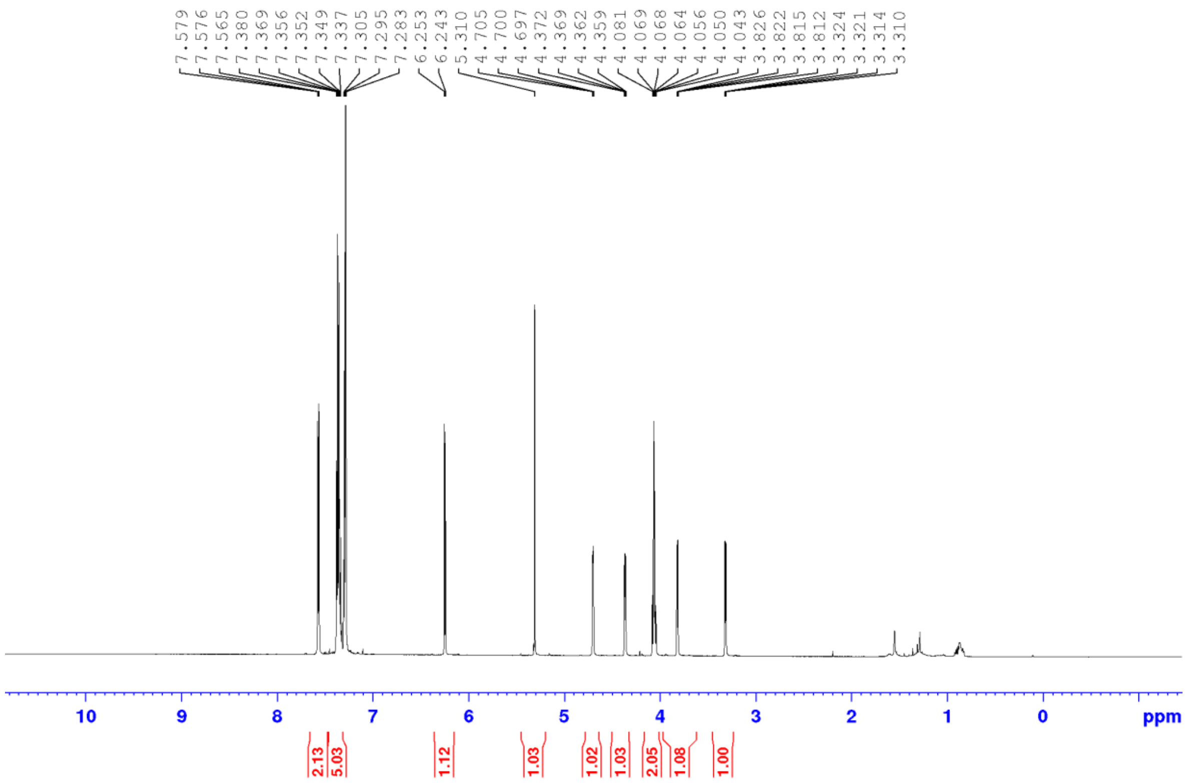

Figure S1a. The ^{1}H-NMR spectrum of (*exo*,*exo*)**-3a**.

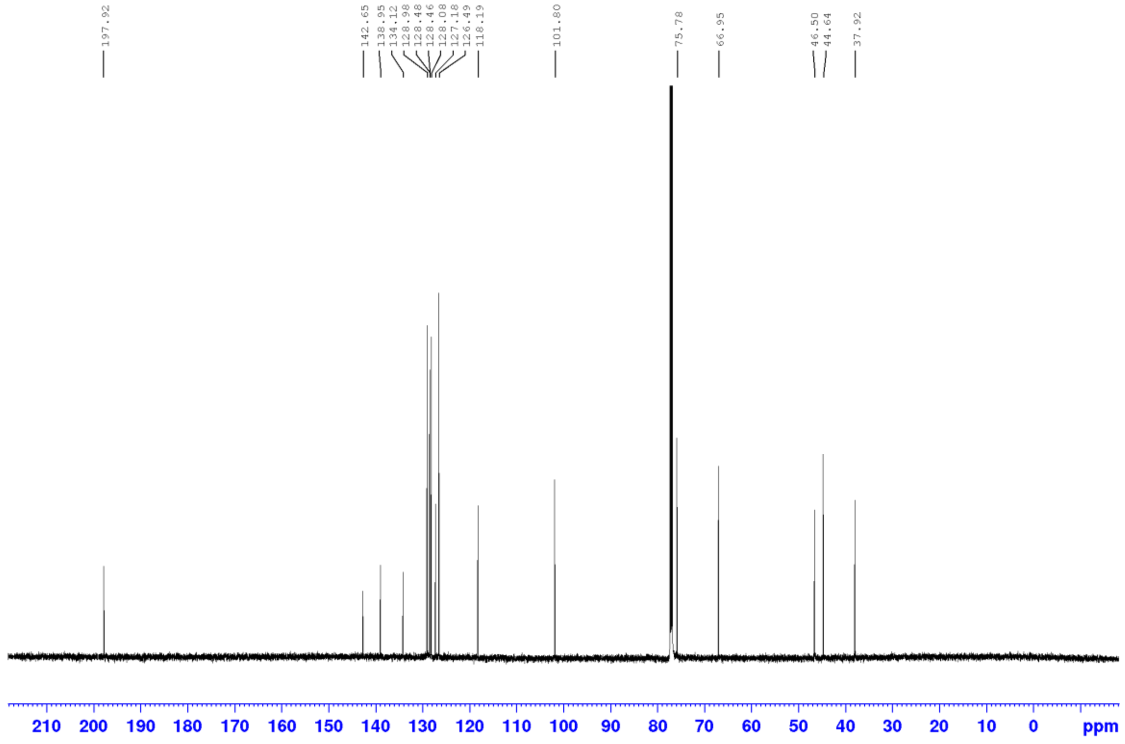

Figure S1b. The ^{13}C-NMR spectrum of (*exo*,*exo*)**-3a**.

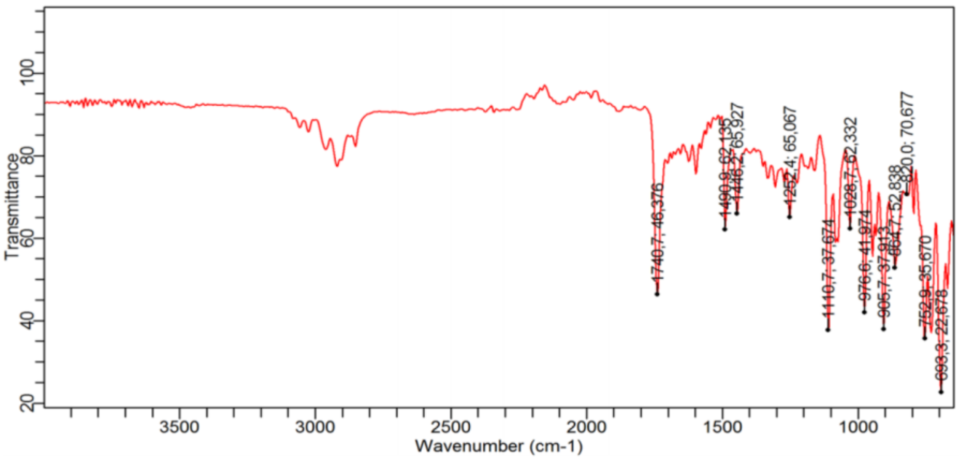

Figure S1c. The IR spectrum of (*exo*,*exo*)-**3a**.

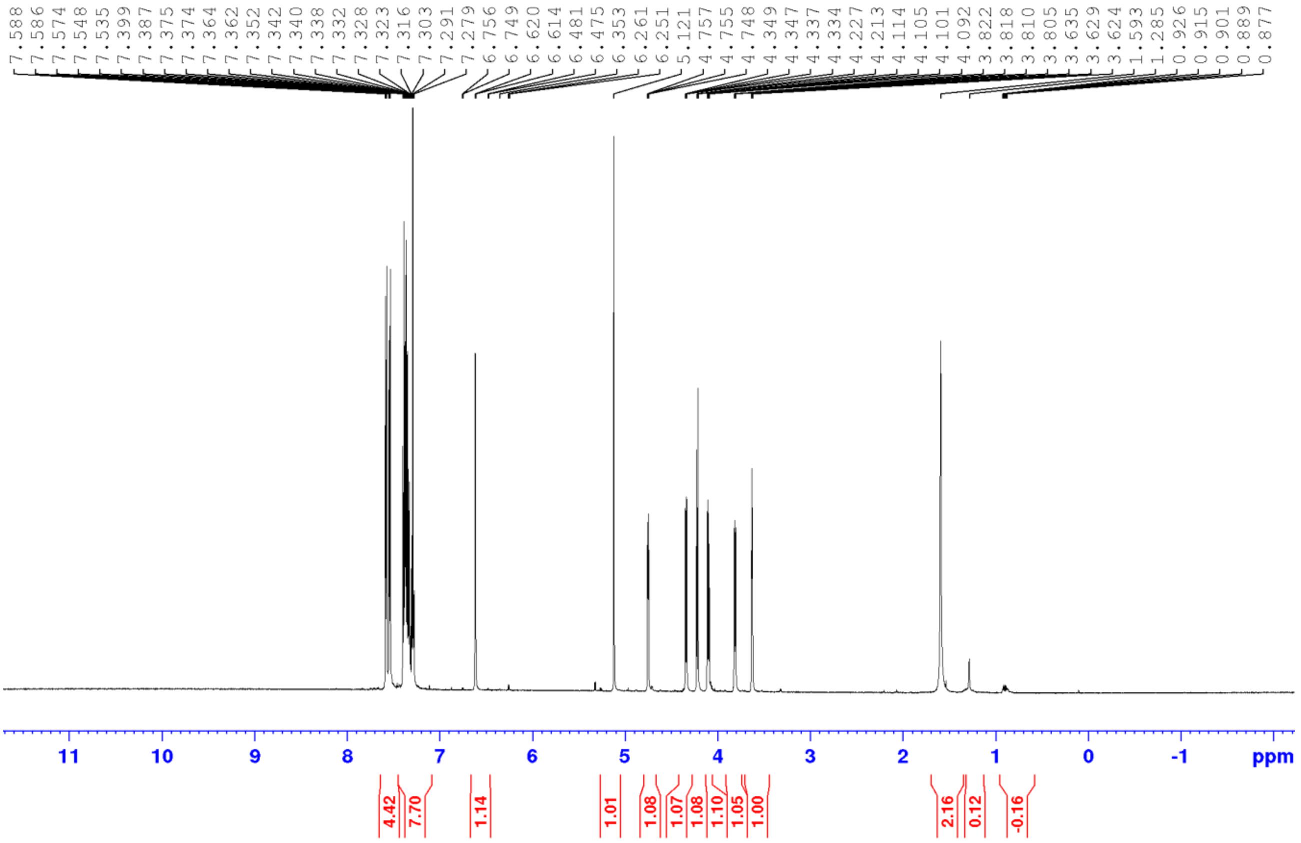

Figure S2a. The ^{1}H NMR spectrum of (*exo*,*endo*)**-3a**.

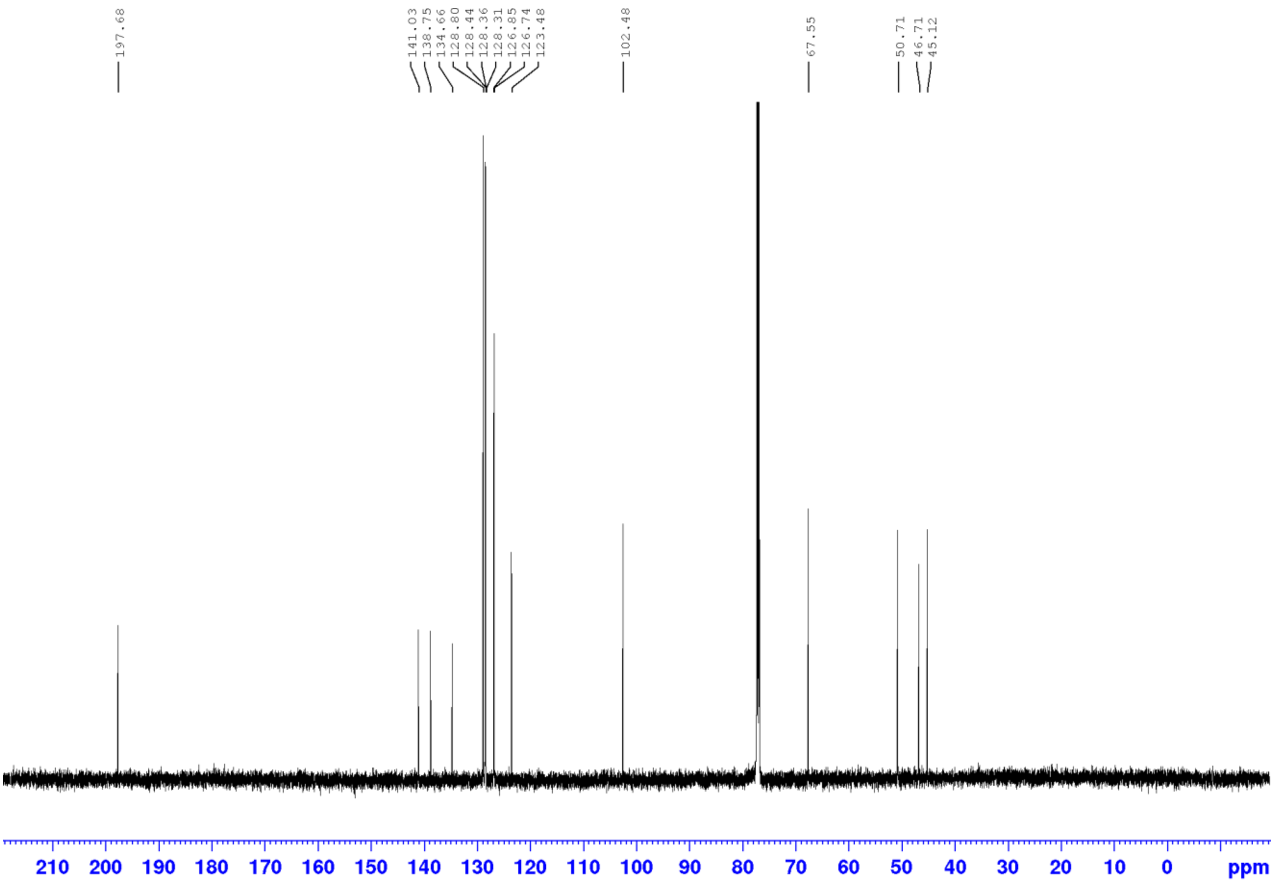

Figure S2b. The ^{13}C NMR spectrum of (*exo*,*endo*)-**3a**.

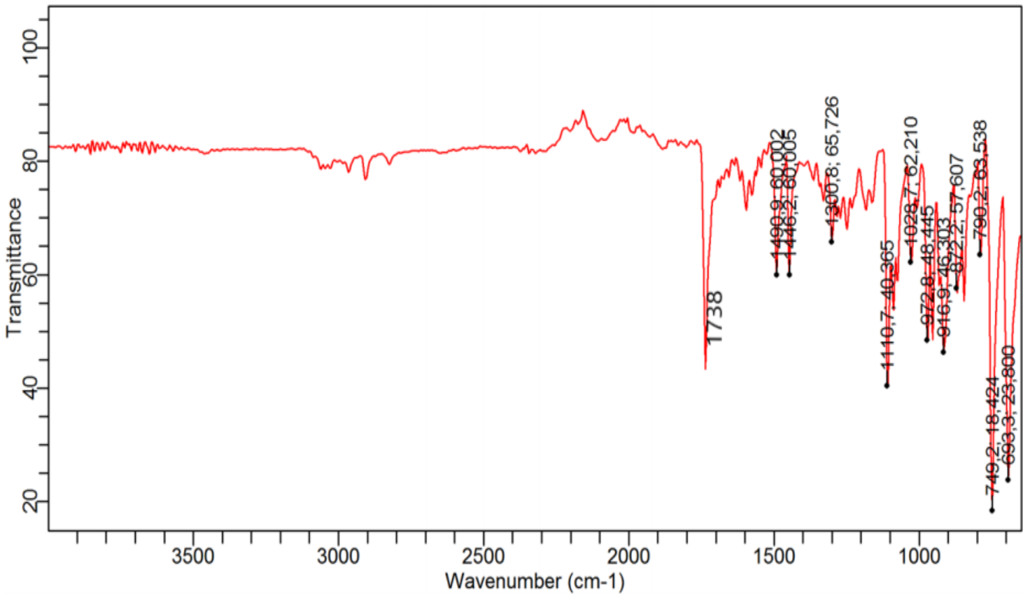

Figure S2c. The IR spectrum of (*exo*,*endo*)-**3a**.

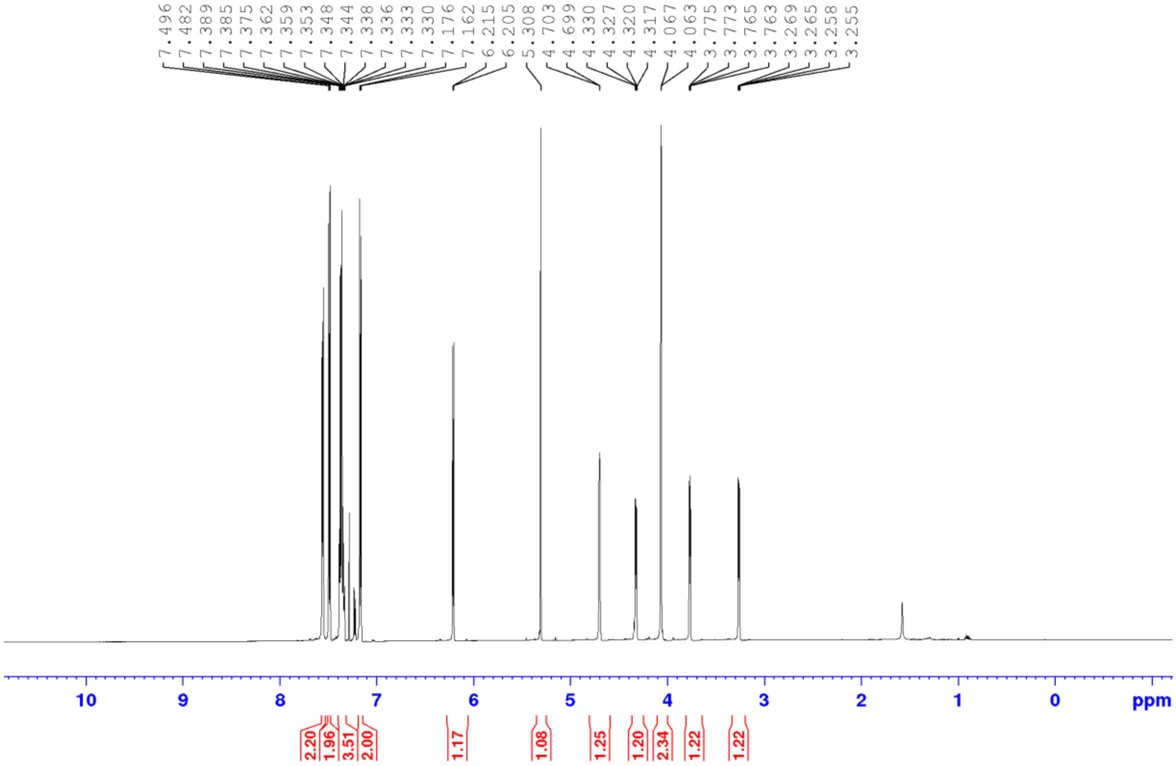

Figure S3a. The ^{1}H NMR spectrum of (*exo*,*exo*)-**3b**.

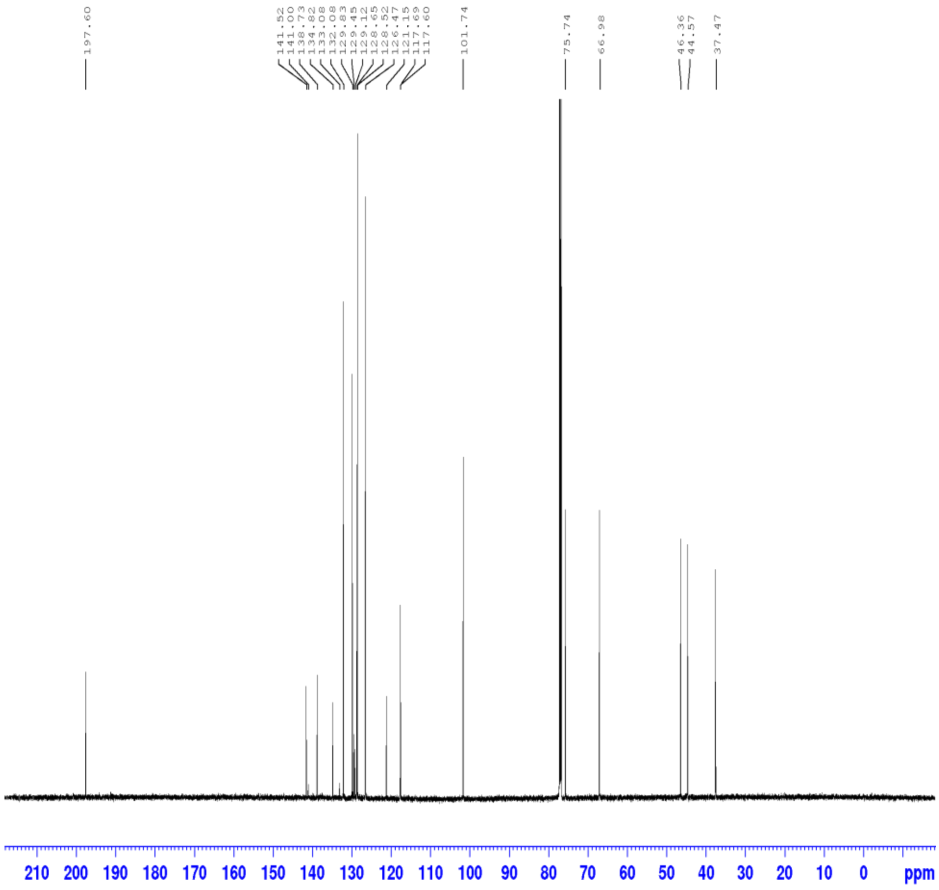

Figure S3b. The ^{13}C NMR spectrum of (*exo*,*exo*)-**3b**.

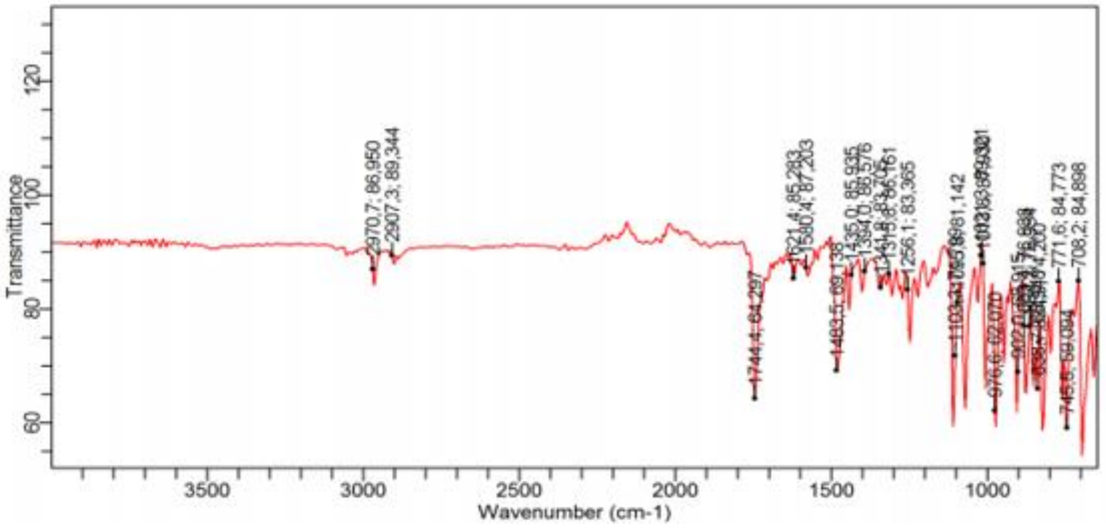

Figure S3c. The IR spectrum of (*exo*,*exo*)-**3b**.

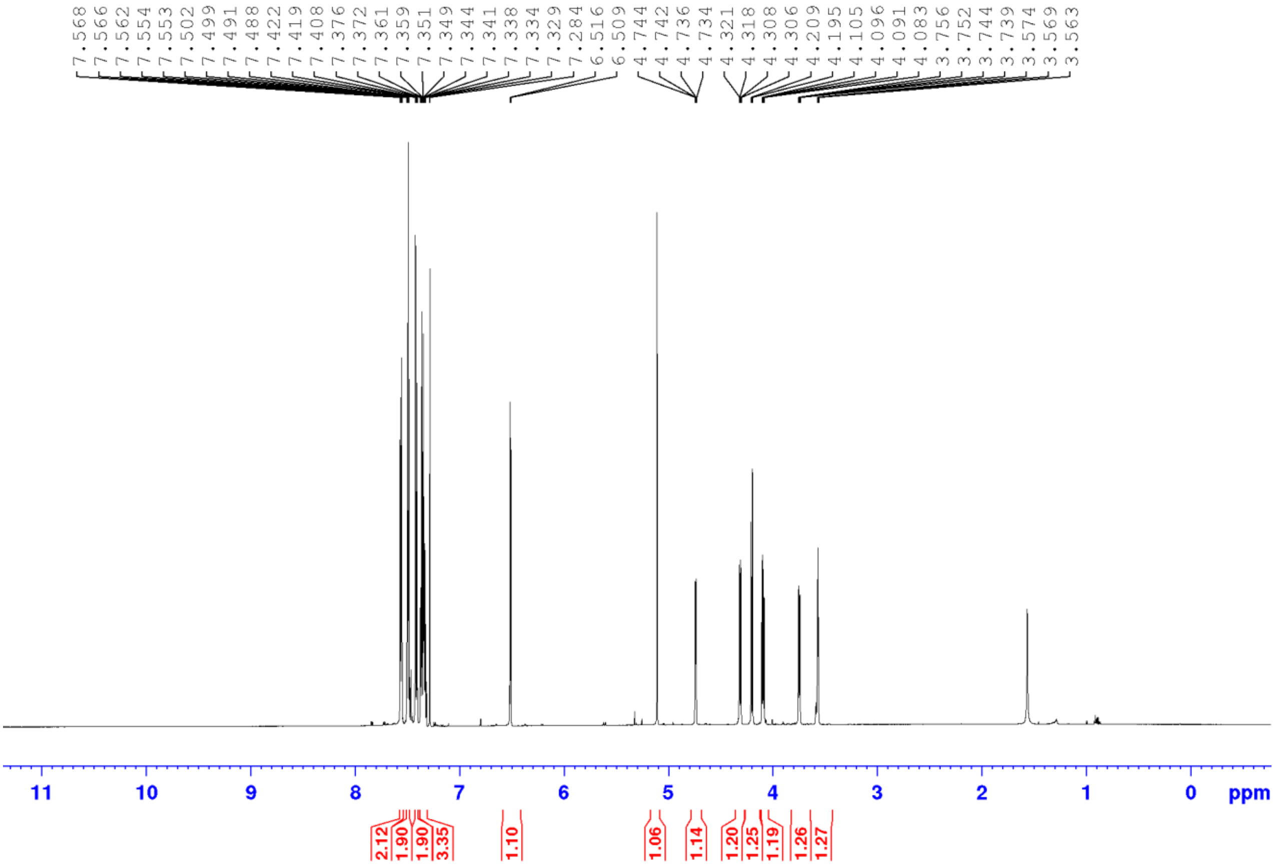

Figure S4a. The ^{1}H NMR spectrum of (*exo*,*endo*)**-3b**.

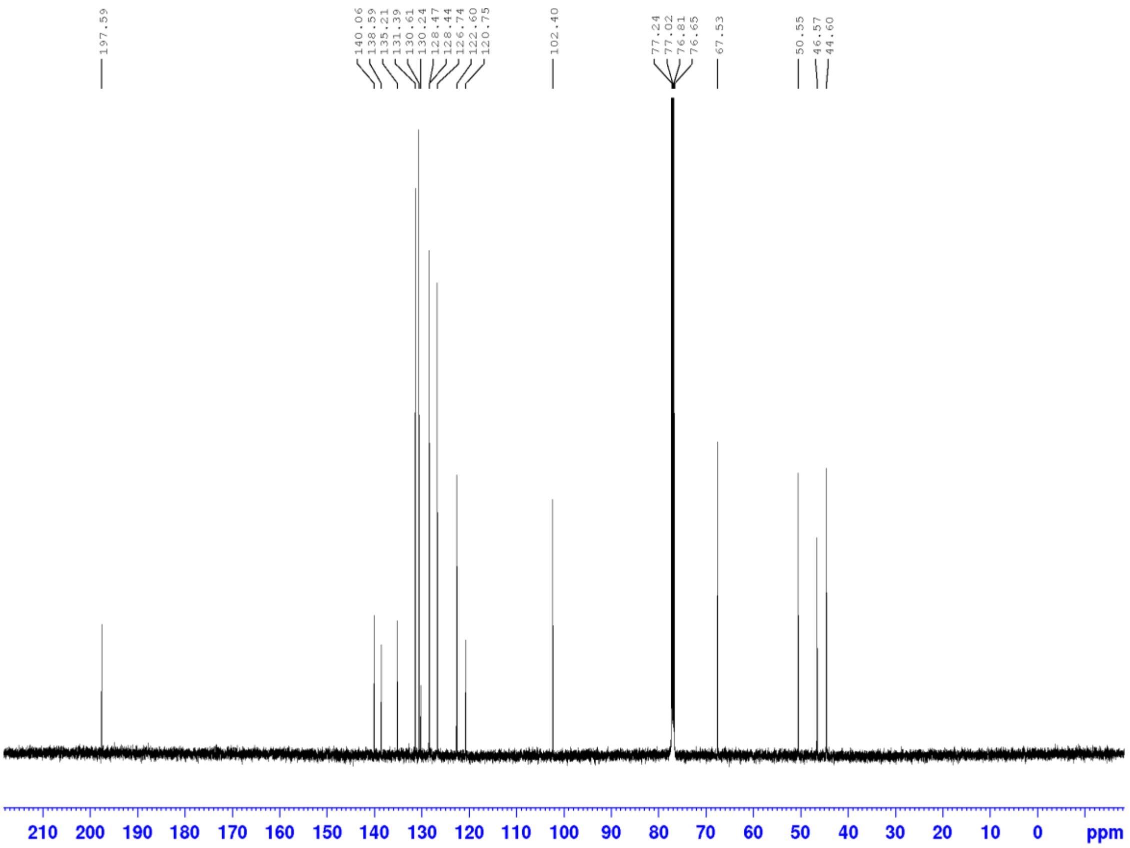

Figure S4b. The ^{13}C NMR spectrum of (*exo*,*endo*)-**3b**.

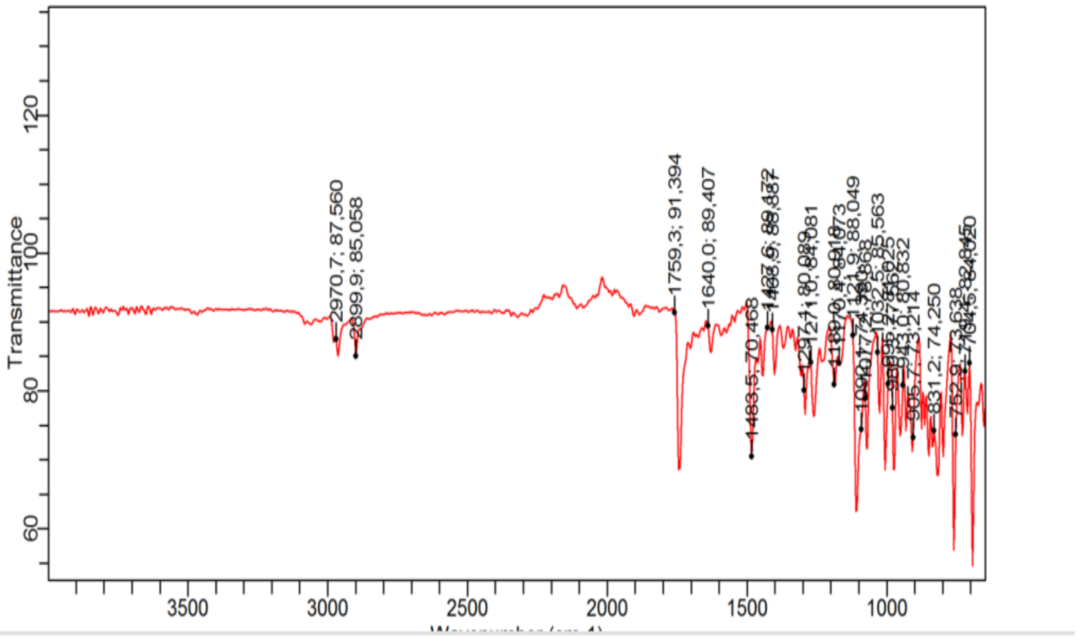

Figure S4c. The IR spectrum of (*exo*,*endo*)-**3b**.

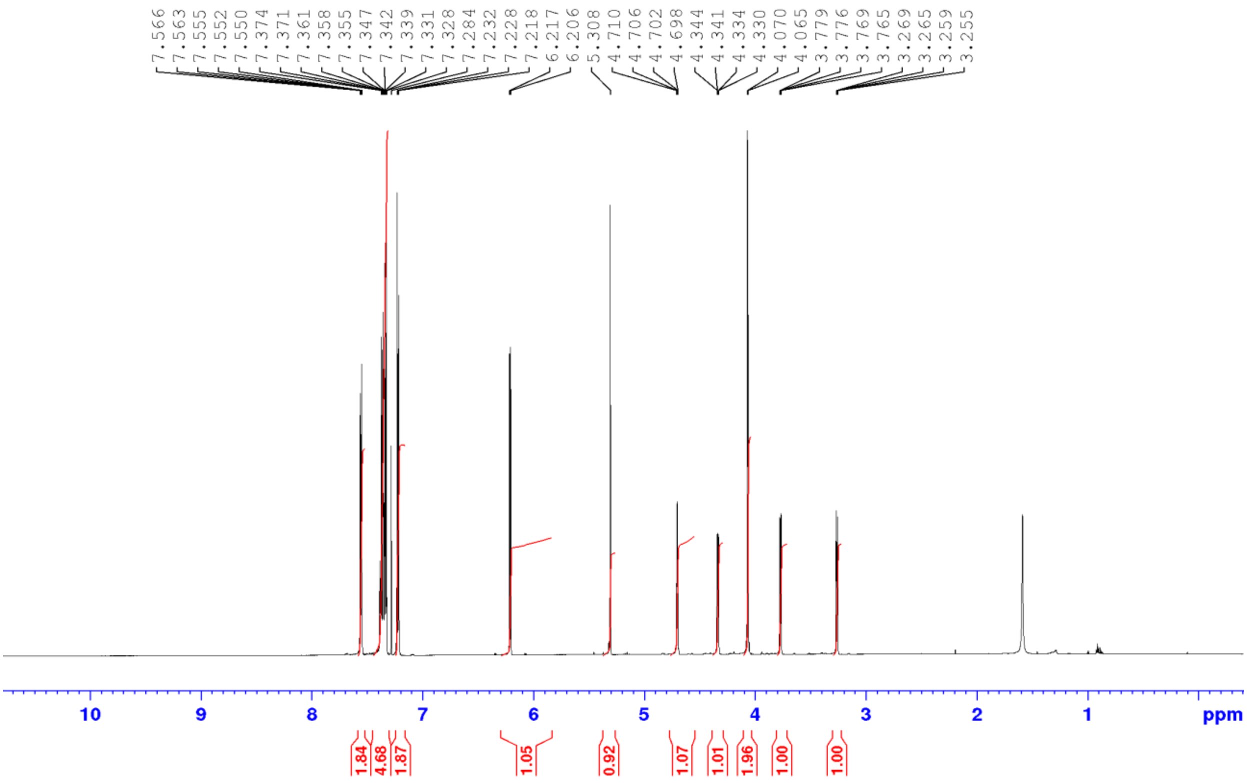

Figure S5a. The ^{1}H NMR spectrum of (*exo*,*exo*)**-3c**.

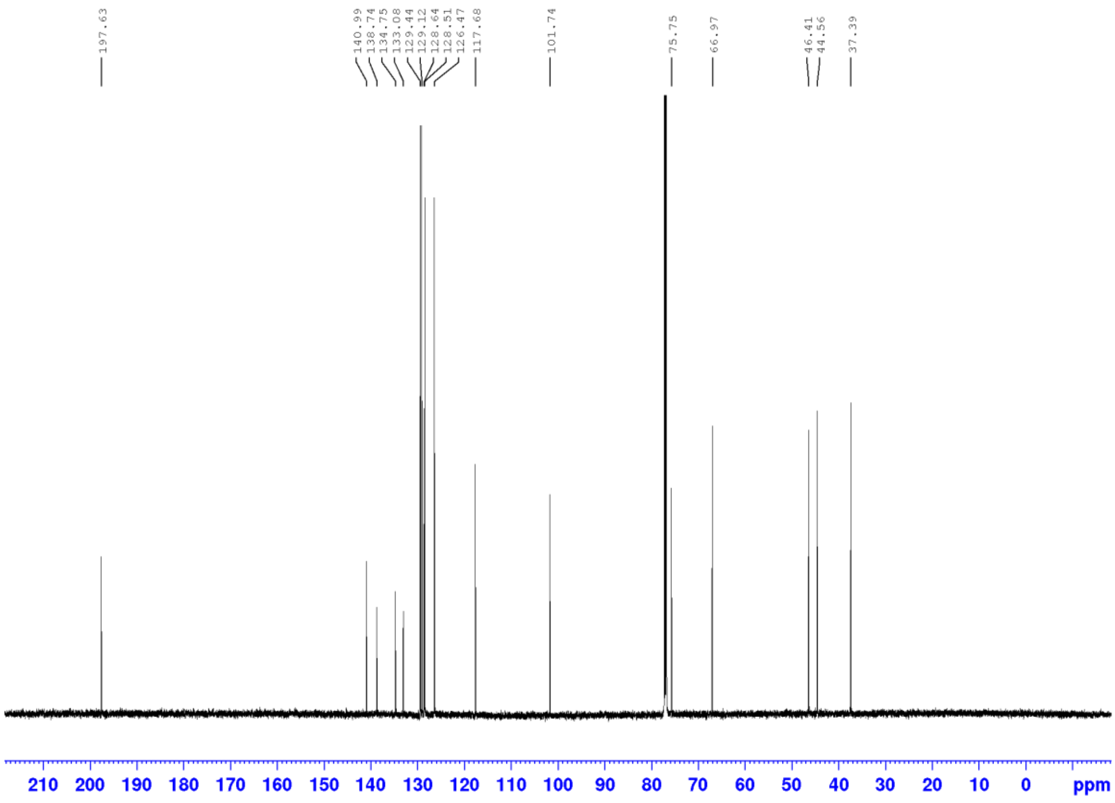

Figure S5b. The ^{13}C NMR spectrum of (*exo*,*exo*)**-3c**.

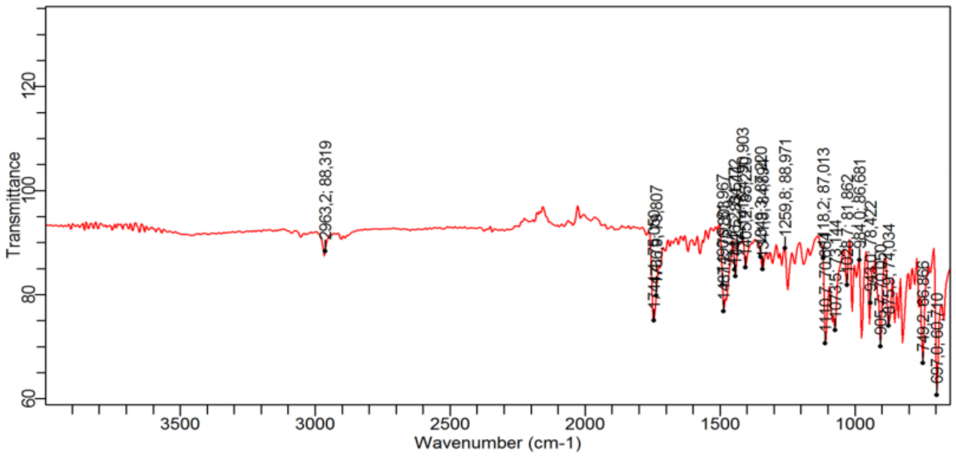

Figure S5c. The IR spectrum of (*exo*,*exo*)-**3c**.

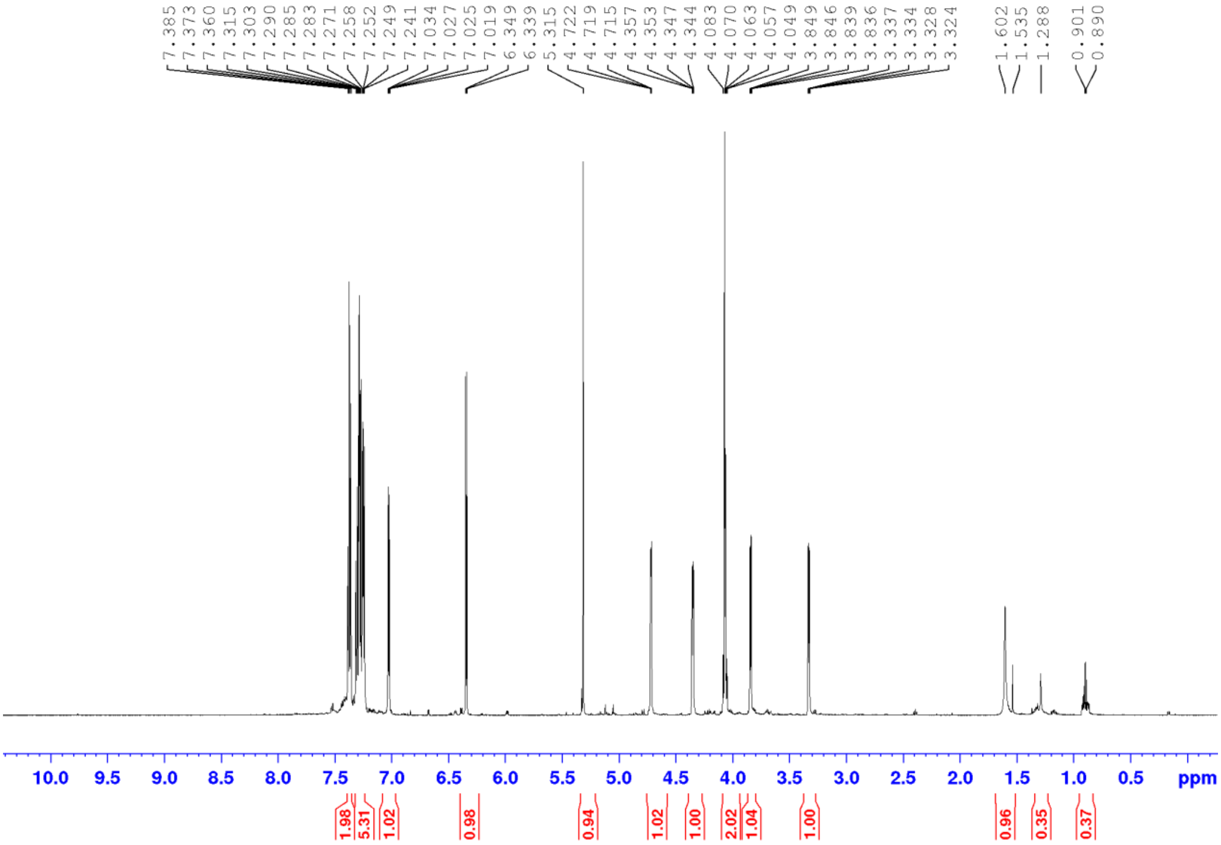

Figure S6a. The ^{1}H NMR spectrum of (*exo*,*exo*)-**3d**.

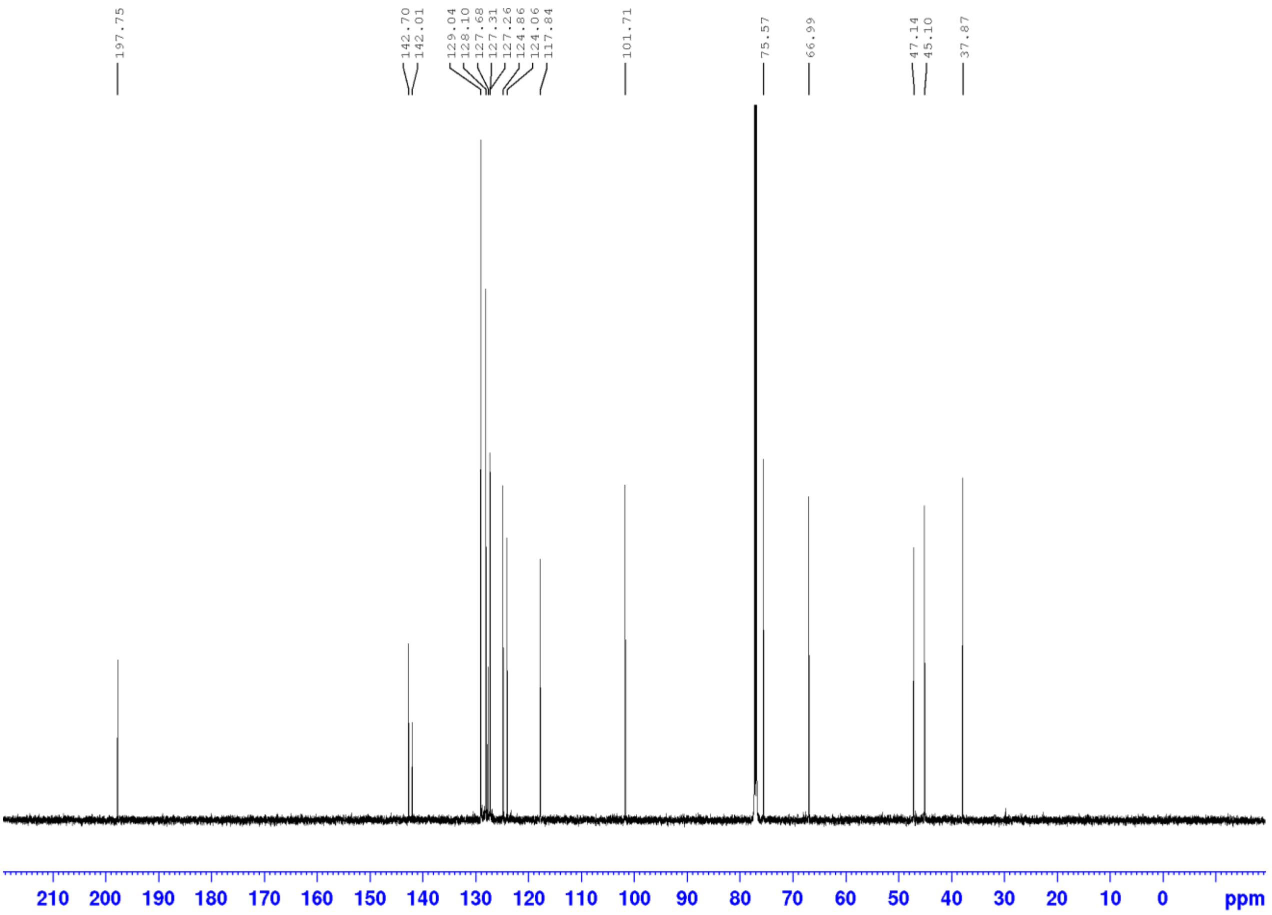

Figure S6b. The ^{13}C NMR spectrum of (*exo*,*exo*)**-3d**.

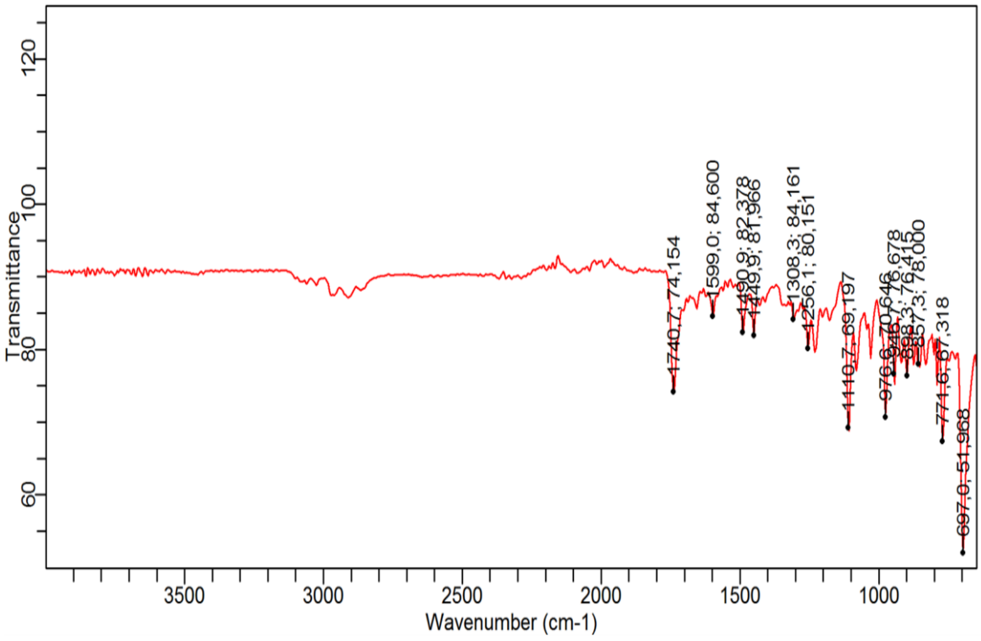

Figure S6c. The IR spectrum of (*exo*,*exo*)-**3d**.

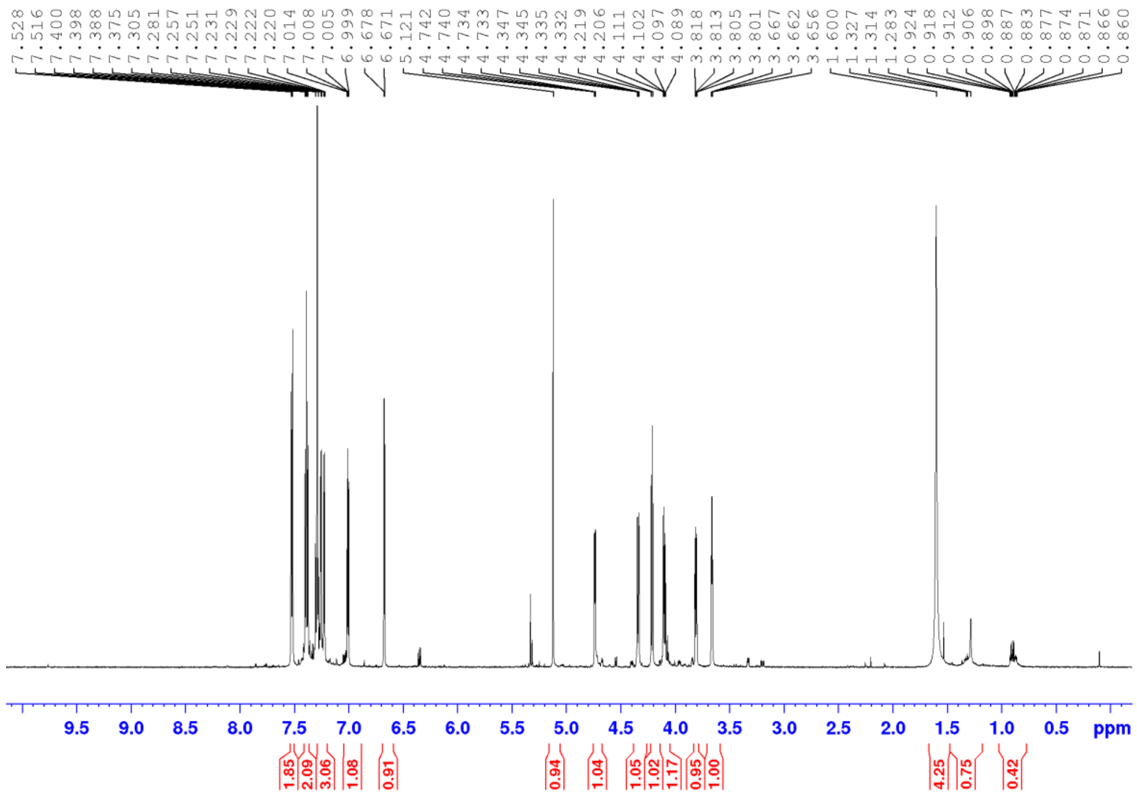

Figure S7a. The ^{1}H NMR spectrum of (*exo*,*endo*)-**3d**.

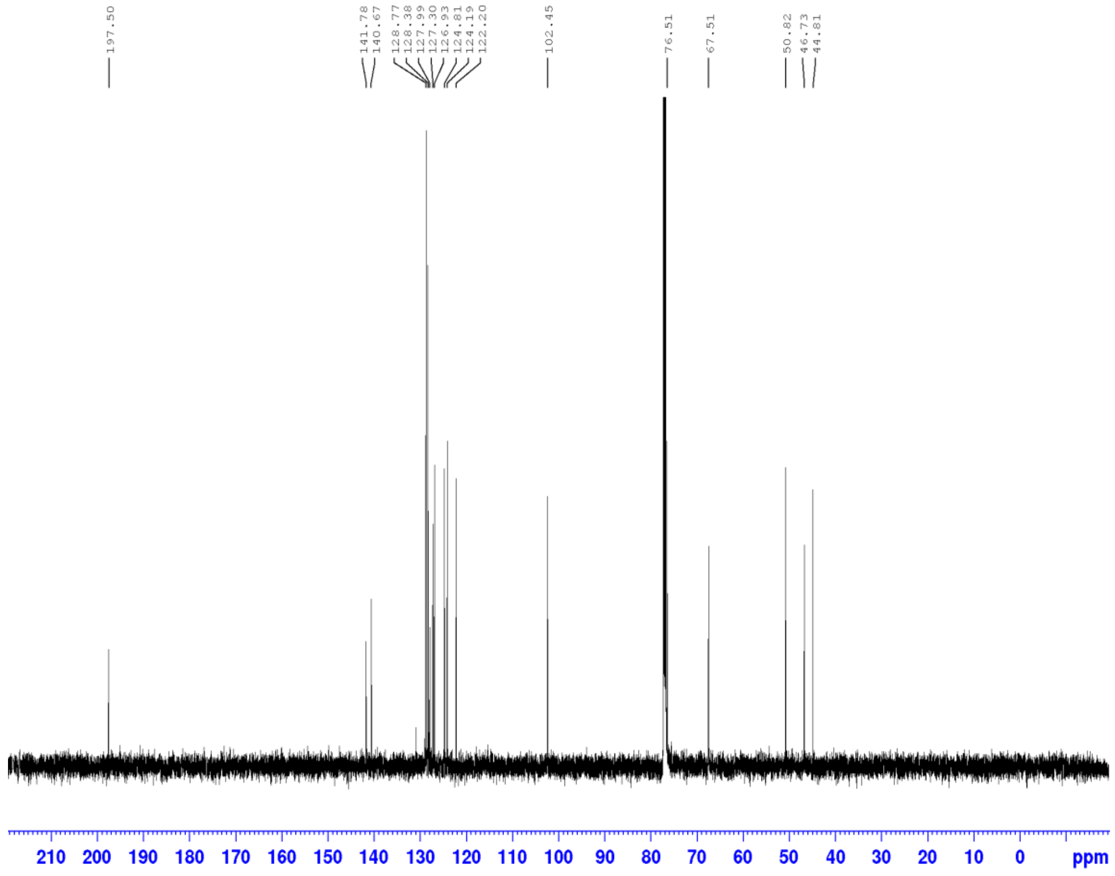

Figure S7b. The ^{13}C NMR spectrum of (*exo*,*endo*)-**3d**.

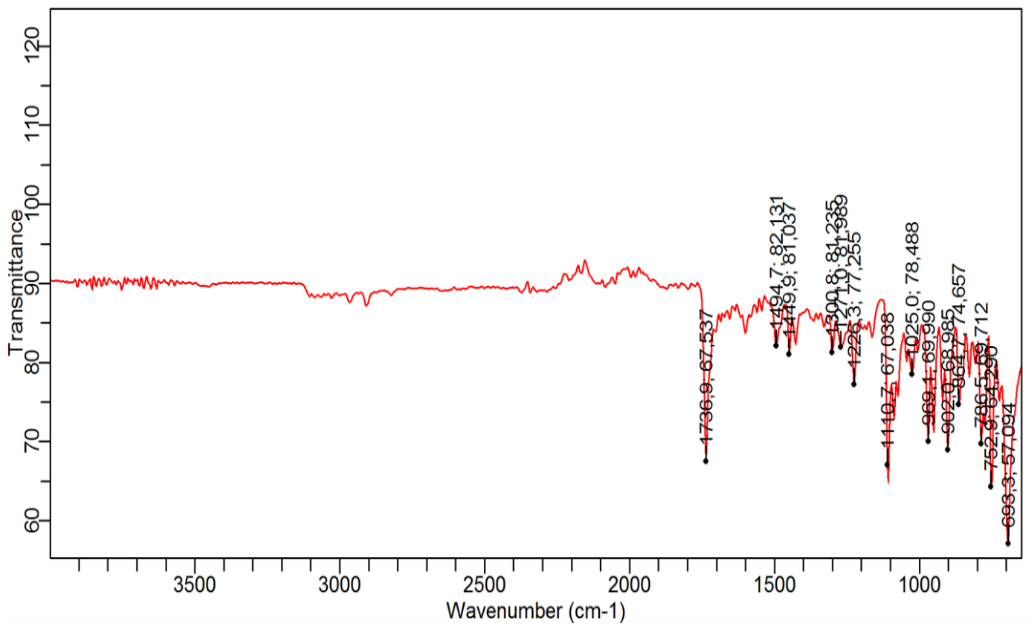

Figure S7c. The IR spectrum of (*exo*,*endo*)-**3d**.

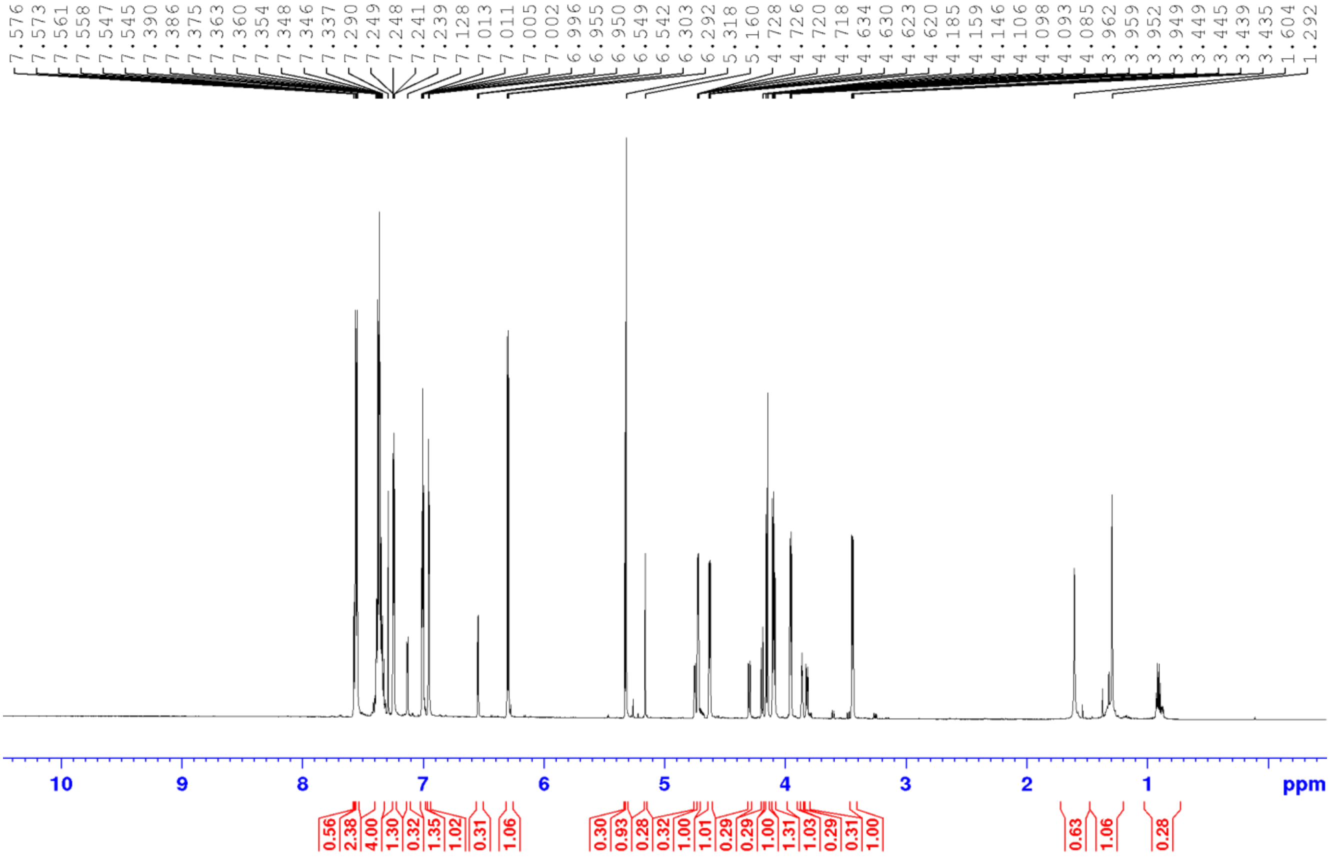

Figure S8a. The ^{1}H NMR spectrum of the purified mixture of (*exo*,*exo*)-**3e** and (*exo*,*endo*)-**3e** (ratio ca. 2:1).

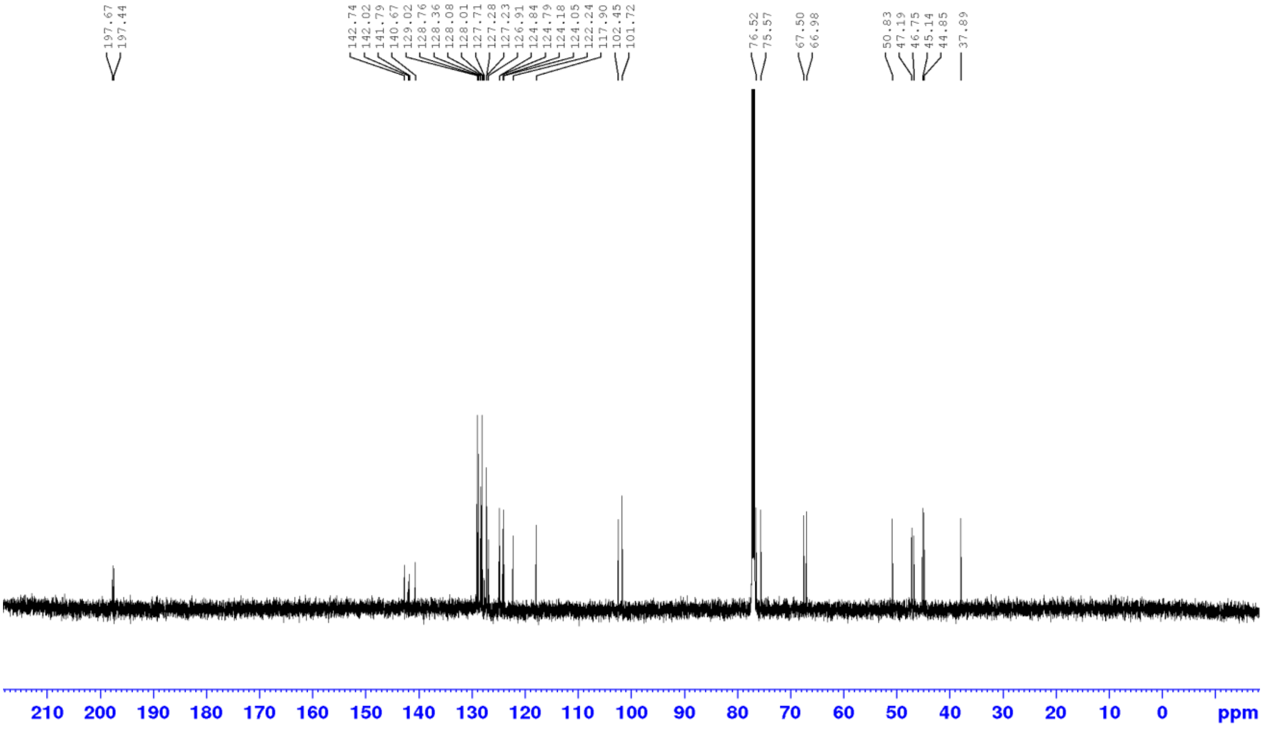

Figure S8b. The ^{13}C NMR spectrum of the purified mixture of (*exo*,*exo*)-**3e** and (*exo*,*endo*)-**3e** (ratio ca. 2:1).

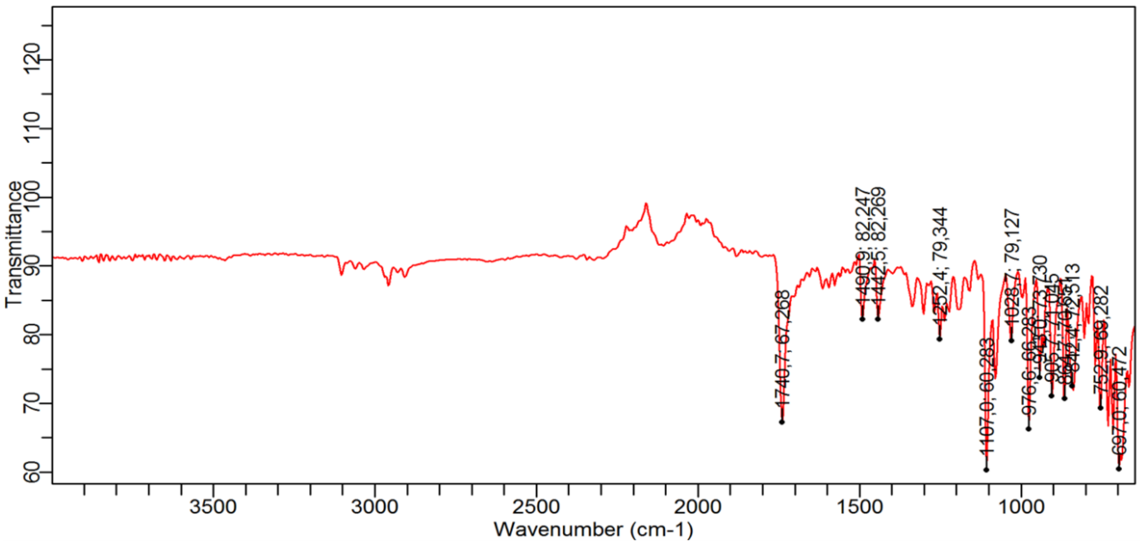

Figure S8c. The IR spectrum of the purified mixture of (*exo*,*exo*)-**3e** and (*exo*,*endo*)-**3e** (ratio ca. 2:1).

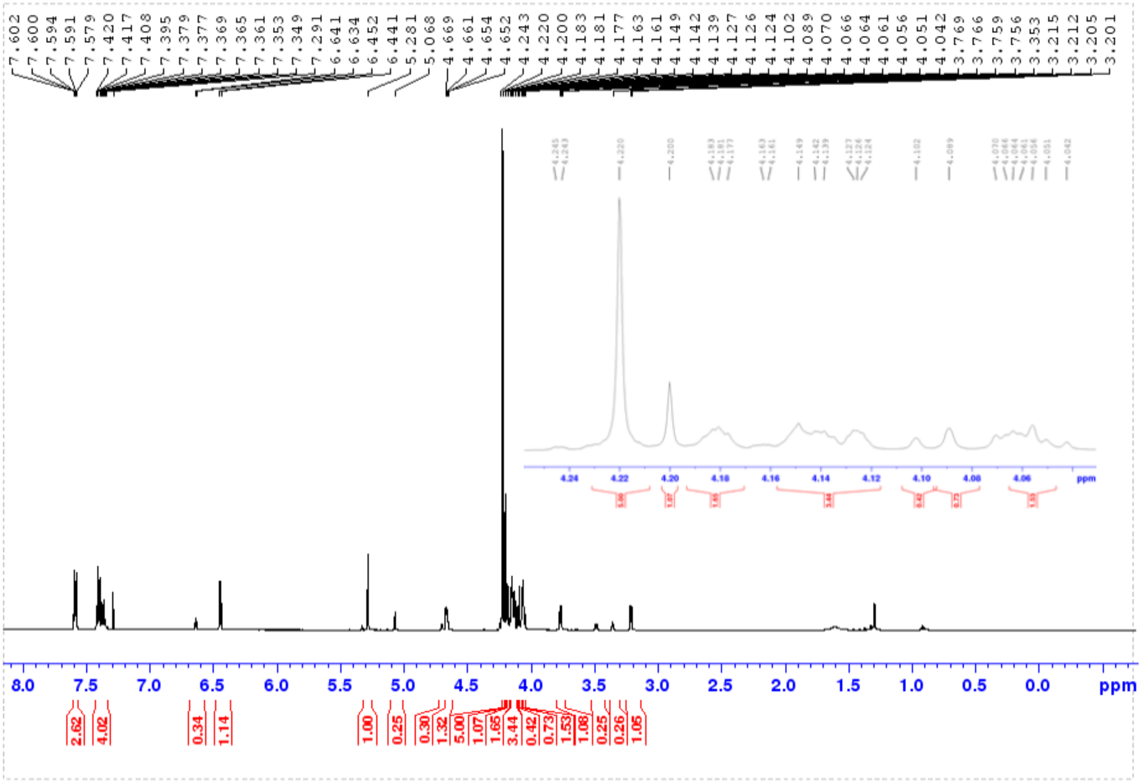

Figure S9a. The ^{1}H NMR spectrum of the purified mixture of (*exo*,*exo*)-**3f** and (*exo*,*endo*)-**3f** (ratio ca. 5:1).

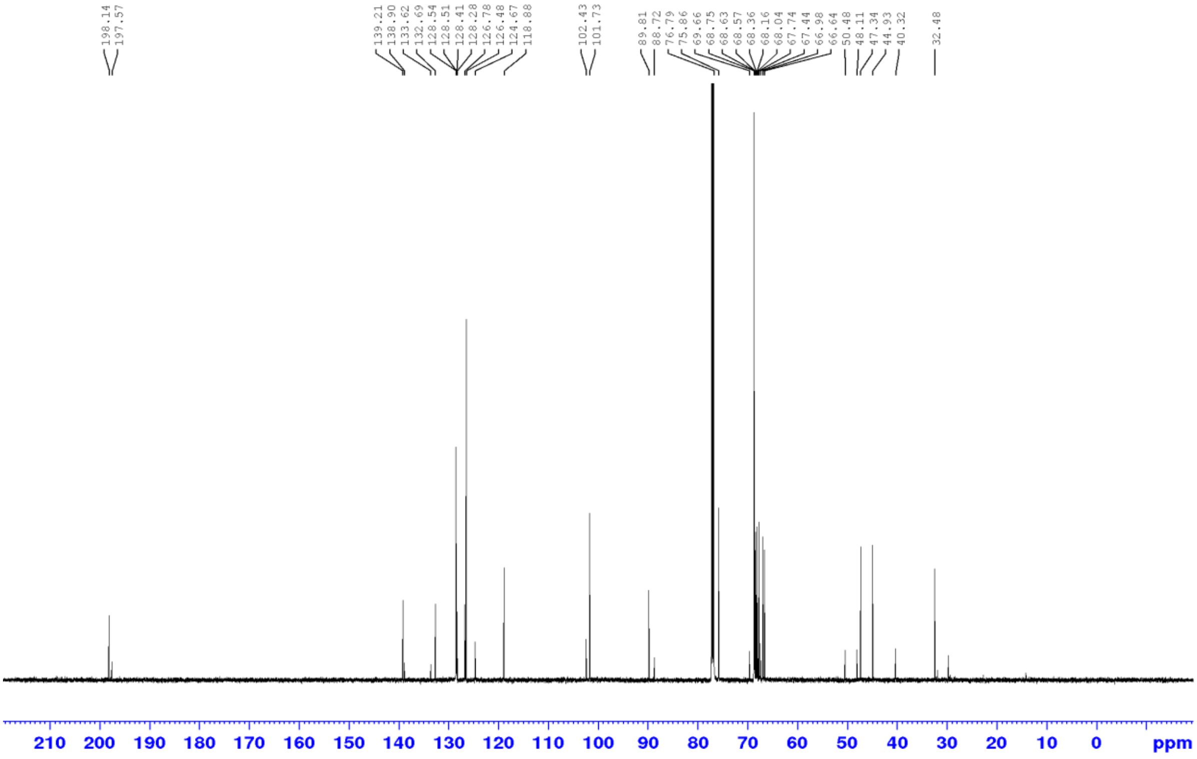

Figure S9b. The ^{13}C NMR spectrum of the purified mixture of (*exo*,*exo*)-**3f** and (*exo*,*endo*)-**3f** (ratio ca. 5:1).

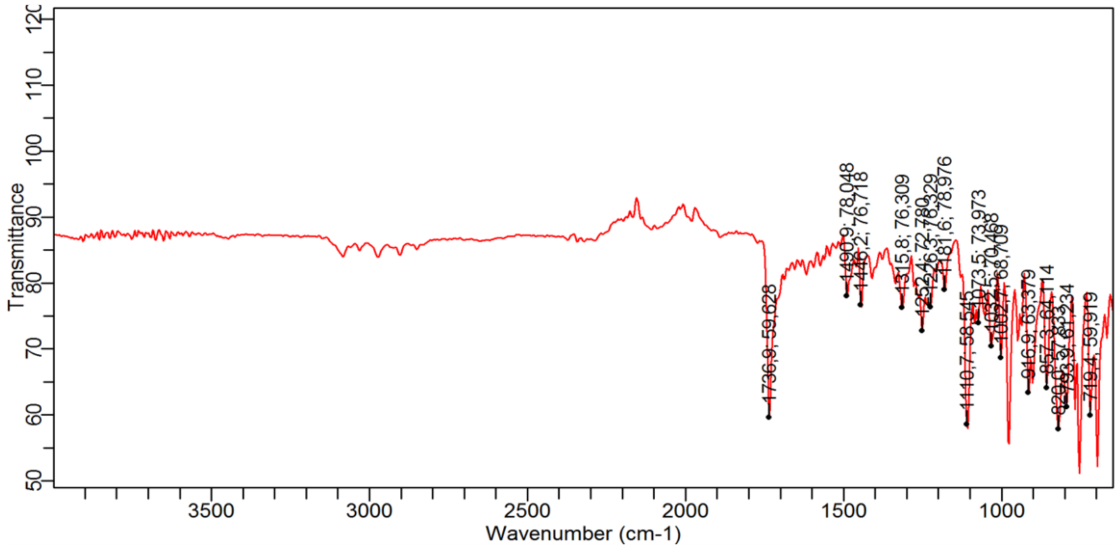

Figure S9c. The IR spectrum of the purified mixture of (*exo*,*exo*)-**3f** and (*exo*,*endo*)-**3f** (ratio ca. 5:1).

Section 5: *X-Ray structure determination of compounds (exo,exo)-**3b** and (exo,endo)-**3b***

Description of X-ray data collection experiments: X-ray diffraction data for (*exo,exo*)-**3b** and (*exo,endo*)-**3b** was collected on an XtaLAB Synergy, Dualflex, HyPix diffractometer. Integration of the intensities and corrections for Lorentz effects, polarization effects, and analytical absorption were performed with CrysAlis PRO [4]. Using Olex2 [5], the structure was solved with the SHELXT [6] structure solution program using Intrinsic Phasing and refined with the SHELXL [7] refinement package using Least Squares minimization. The hydrogen atoms were introduced in the calculated positions with an idealized geometry and constrained using a rigid body model with isotropic displacement parameters equal to 1.2 of the equivalent displacement parameters of their parent atoms. The molecular geometries were calculated by the PLATON program [8]. The relevant crystallographic data are given in Table S1 (SI). Atomic coordinates, displacement parameters, and structural factors of the analyzed crystal structures are deposited with the Cambridge Crystallographic Data Centre CCDC (reference number: 2471272 and 2471271 for (*exo,exo*)-**3b** and (*exo,endo*)-**3b**, respectively) [9].

Table S1. Crystal data and structure refinement for *(exo,exo)*-**3b,** and (*exo*,*endo*)-**3b**.

Identification code	(*exo,exo*)-**3b**	(*exo*,*endo*)-**3b**
Empirical formula	$C_{21}H_{17}BrO_3S$	$C_{21}H_{17}BrO_3S$
Formula weight	429.32	429.32
Temperature/K	294.22(10)	294.21(10)
Crystal system	orthorhombic	orthorhombic
Space group	P $2_12_12_1$	P $2_12_12_1$
a/Å	6.15250(10)	6.57040(10)
b/Å	12.55870(10)	15.45580(10)
c/Å	24.1965(3)	18.5614(2)
α/°	90	90
β/°	90	90
γ/°	90	90
Volume/Å^3	1869.60(4)	1884.92(4)
Z	4	4
ρ_{calc}mg/mm^3	1.525	1.513
μ/mm^{-1}	4.189	4.155
F(000)	872.0	872.0
Crystal size/mm^3	0.5 × 0.4 × 0.4	0.5 × 0.5 × 0.3
2Θ range for data collection	7.306 to 152.832°	7.444 to 153.422°
Index ranges	-5 ≤ h ≤ 7, -15 ≤ k ≤ 15, -30 ≤ l ≤ 29	-7 ≤ h ≤ 7, -19 ≤ k ≤ 13, -23 ≤ l ≤ 23
Reflections collected	17995	18342
Independent reflections	3761 [Rint = 0.0374, Rsigma = 0.0240]	3788 [Rint = 0.0363, Rsigma = 0.0262]
Data/restraints/parameters	3761/0/235	3788/0/236
Goodness-of-fit on F^2	1.078	1.058
Final R indexes [I>=2σ (I)]	R1 = 0.0339, wR2 = 0.0969	R1 = 0.0365, wR2 = 0.1022
Final R indexes [all data]	R1 = 0.0354, wR2 = 0.0983	R1 = 0.0375, wR2 = 0.1033
Largest diff. peak/hole / e Å^{-3}	0.27/-0.64	0.49/-0.59
Flack parameter	-0.044(8)	-0.023(8)
CCDC number	2471272	2471271

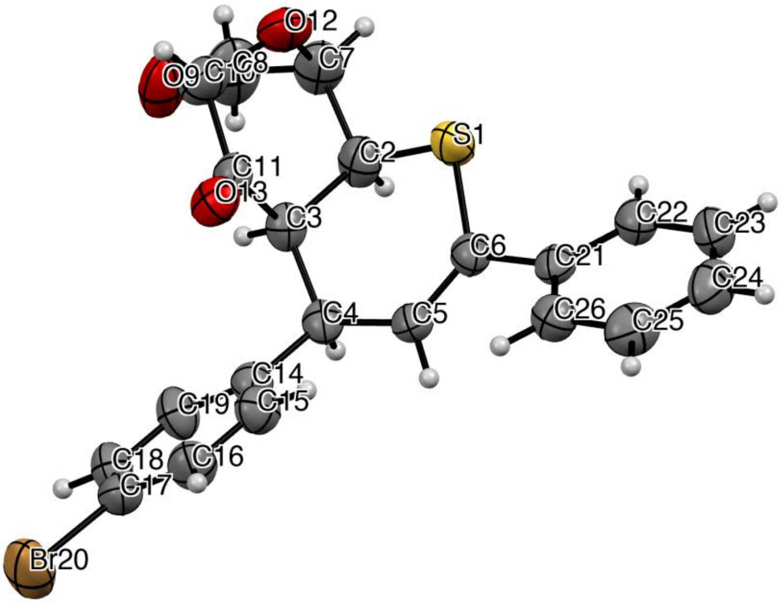

(a)

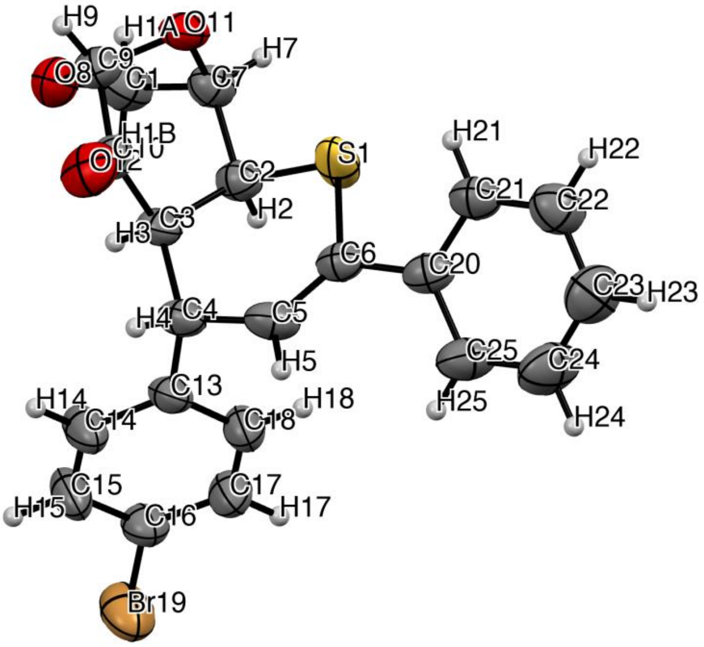

(b)

Figure S10. ORTEP molecular graphs with crystallographic labelling scheme for (*exo*,*exo*)-**3b** (a) and (*exo*,*endo*)-**3b** (b). Ellipsoids drawn with 50% probability.

Section 6: *DFT calculations*

All calculations were performed using the GAUSSIAN 16, B.01 package of programs [10]. The structures were fully optimized using the PBE1PBE/def2tzvp [11–15] + GD3BJ [16, 17] functional including the PCM-solvent sphere for dichloromethane [18]. Zero point vibrational energies and free enthalpy contributions were determined analytically.

Table S2. Total energies (E_{tot}) and Gibbs free energies (G_{298}) [a.u.], of all compounds and of the corresponding transition states as given in the Gaussian archive entries. The relative energies [kcal/mol] include all involved species according to the respective reactions as given in the schemes in the manuscript.

**Species**	**E_{tot} [a.u.]**	**E_{rel} [kcal/mol]**	**G_{298} [a.u.]**	**E_{rel} [kcal/mol]**
**LGO**	- 457,56164		-457,47656	
**Thiochalcone**	-976,40693		-976,22627	
**TS**-*(exo,exo)*-**3a**	-1.433,96885	-0,18	-1.433,67933	14,75
(exo,exo)-**3a**	-1.434,02786	-37,21	-1.433,73339	-19,17
**TS**-*(exo-endo)*-**3a**	-1.433,96756	0,63	-1.433,67782	15,70
(exo-endo)-**3a**	-1.434,02154	-33,24	-1.433,72686	-15,08
**TS**-*(endo-exo)*-**3a**	-1.433,94429	15,24	-1.433,65432	30,44
(endo-exo)-**3a**	-1.434,01170	-27,06	-1.433,71664	-8,66
**TS**-*(endo-endo)*-**3a**	-1.433,96195	4,15	-1.433,67130	19,78
(endo-endo)-**3a**	-1.434,01762	-30,78	-1.433,72422	-13,42
**TS**-*(exo-exo)*-**4a**	-1.433,96560	1,86	-1.433,67568	17,04
(exo,exo)-**4a**	-1.434,02339	-34,40	-1.433,72842	-16,06
**TS**-*(exo-endo)*-**4a**	-1.433,95057	11,29	-1.433,66120	26,13
(exo-endo)-**4a**	-1.434,02710	-36,73	-1.433,73270	-18,74
**TS**-*(endo-exo)*-**4a**	-1.433,95202	10,39	-1.433,66280	25,12
(endo-exo)-**4a**	-1.434,02274	-33,99	-1.433,72776	-15,64
**TS**-*(endo-endo)*-**4a**	-1.433,95357	9,41	-1.433,66361	24,61
(endo-endo)-**4a**	-1.434,02105	-32,93	-1.433,72627	-14,70

Gaussian Archive Entries

(Total energies (a.u.), number of imaginary frequencies (for transition states: imaginary frequencies), coordinates)

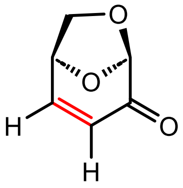

1 (LGO)

HF=-457.5616357 a.u. NIMAG=0
1\1\GINC-R10N01\FOpt\RPBE1PBE\def2TZVP\C6H6O3\WURTHWE\06-Mar-2022\0\\#
pbe1pbe/def2tzvp Opt=readfc geom=check guess=read Pop=NBO Freq emp=gd3bj
scrf=(solvent=thf)\\ Educt LGO 1\\0,1\
C,0.0324783281,0.0241590837,4.5042473707\C,0.341986787,0.6047541116,5.6669562943\
C,-0.6771393736,-1.2590911069,4.5298474853\C,-0.991216242,-1.7525020515,5.9434727114\
O,0.0895592737,-1.4978716891,6.785151999\C,-0.0011542082,-0.0837295992,6.9503232044\
O,-2.0692442941,-0.9641728657,6.4293337986\C,-1.5009280333,0.0550928928,7.2429583135\
O,-1.0449905741,-1.8616915353,3.5448793365\H,-1.9142001696,1.0242498172,6.9625676941\
H,-1.7054000339,-0.1523151773,8.2961262506\H,0.6386124874,0.2156640906,7.7773473354\
H,-1.2495938328,-2.8117909032,5.9568828578\H,0.8067184061,1.5836908338,5.7131419158\
H,0.2601933412,0.4681856117,3.542329786\\Version=ES64L-G16RevB.01\State=1-A\HF=-
457.5616357\RMSD=9.296e-09\RMSF=1.620e-06\Dipole=0.7218885,1.6675711,1.4026357\
Quadrupole=-1.3648581,3.5563917,-2.1915337,1.5856853,-1.846897,-1.0916828\PG=C01
[X(C6H6O3)]\\@

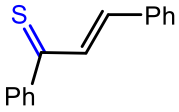

2a

HF=-976.4069321 a.u., NIMAG=0
1\1\GINC-R01N13\FOpt\RPBE1PBE\def2TZVP\C15H12S1\WURTHWE\09-Feb-2022\0\
\# pbe1pbe/def2tzvp Opt=(readfc,maxstep=7) geom=check guess=read Pop=NBO Freq emp=gd3bj
scrf=(solvent=thf)\\Thiochalcone 2a; s-cis-E\\0,1\
C,-0.980381896,-1.6309498359,0.4035950771\C,-2.3023159982,-1.6946266783,0.648322906\
C,-0.1351654811,-2.7903642621,0.2021353381\S,-0.7207340392,-4.3179669682,-0.0132413501\
C,1.3182117052,-2.5359726413,0.189031736\C,2.1738786277,-3.3158497092,-0.5928970973\
C,3.5369476886,-3.0840913418,-0.593037903\C,4.0748098013,-2.0828278379,0.2066056242\
C,3.2382699945,-1.3080135908,0.9972396163\C,1.8698420989,-1.5214476445,0.9776760798\
C,-3.7246394573,1.7767696773,0.9352511671\C,-2.8108912876,0.7588874301,0.7453083016\
C,-3.2089192005,-0.5817947947,0.8354320687\C,-4.5506052729,-0.8605816458,1.1195496933\
C,-5.4658881765,0.1605923762,1.3097317025\C,-5.0550131238,1.4824594993,1.2185514241\
H,-0.4897154226,-0.6664787566,0.341675 2134\H,-2.7437401113,-2.6862068689,0.7183860694\
H,1.7462491193,-4.0978533748,-1.2082786441\H,4.1851125874,-3.6860887679,-1.2190507746\

H,5.1442160921,-1.9066920924,0.2118174178\H,3.652193193,-0.5334100533,1.631999765\
H,1.2297860521,-0.9188236385,1.610780518\H,-3.4028956931,2.8089808896,0.8619952543\
H,-1.7800372839,1.0064906099,0.5229400242\H,-4.8687115482,-1.8951104306,1.1894253661\
H,-6.5006675044,-0.0745157503,1.5288647061\H,-5.7678964635,2.2852142015,1.3663507002\\
Version=ES64L-G16RevB.01\State=1-A\HF=-976.4069321\RMSD=6.641e-09\RMSF=6.652e-
07\Dipole=-0 .0458408,2.0090877,0.374942\Quadrupole=13.9801865,-6.1239336,-7.8562529,-
2.9327885,-3.4380801,2.0788812\PG=C01 [X(C15H12S1)]\\@

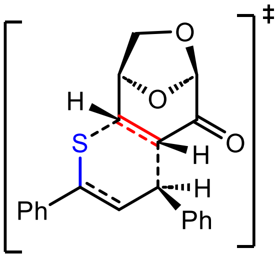

TS-*(exo,exo)*-**3a**

HF=-1433.9688542a.u., NIMAG=1, -241.4911 cm-1

1\1\GINC-R01N21\FTS\RPBE1PBE\def2TZVP\C21H18O3S1\WURTHWE\13-Mar-2022\0
\\# pbe1pbe/def2tzvp Opt(ts,noeigentest,calcfc,maxstep=12) Pop=NBO Fre
q emp=gd3bj scrf=(solvent=thf)\\TS exo,exo-3a\\0,1\C,-0.3936
049053,-0.0644715823,-0.0419402673\C,-0.6560582744,-0.699689725,1.1765
509105\C,0.3056463673,-0.7536697283,2.1693826999\C,1.5537546875,-0.186
7895283,1.9529800396\C,1.8352431403,0.4321523106,0.7395572961\C,0.8732
723937,0.4929756509,-0.2497607065\C,-1.4452399802,-0.0179295435,-1.031
2198219\C,-1.1166168335,-2.0934061462,-2.5752244043\C,-2.4092931906,-2
.2895666642,-3.0489486059\S,-3.6883994384,-0.3913817532,-3.0134620425\
C,-2.5448476973,0.8323267775,-3.036962331\C,-1.5038459523,0.8489098649
,-2.0921674248\C,-3.2814723382,-3.2215447855,-2.2497217816\O,-2.990132
8034,-3.0655224507,-0.8659659048\C,-1.7460747022,-3.6973639894,-0.7697
147055\C,-0.6596814611,-2.8570842527,-1.4425729482\O,-1.87086578,-4.90
78716819,-1.4956039369\C,-2.8813486931,-4.6859859606,-2.4740658793\O,0
.4996712294,-2.9342202354,-1.0651205463\C,-2.5611661532,1.8511844166,-
4.1000175085\C,-3.0424811211,1.5598623717,-5.3780303625\C,-3.039333944
9,2.5247584812,-6.3697771691\C,-2.5724212425,3.8046502876,-6.098916605
3\C,-2.1043345991,4.1108483966,-4.8285478609\C,-2.092435826,3.14207612
73,-3.8391373911\H,-0.3742043156,-1.5723075358,-3.1658086135\H,-2.5756
829443,-2.2831072267,-4.1220524625\H,-4.3429285585,-3.0408532803,-2.40
7771106\H,-1.5039882487,-3.9081343061,0.272266918\H,-2.4881852061,-4.8
715911963,-3.4752413493\H,-3.7231429709,-5.3541838556,-2.278388884\H,-
0.6859706608,1.5403988938,-2.2613812649\H,-2.3435030102,-0.5873863279,
-0.8101068203\H,-3.4101400382,0.5619449307,-5.5840009767\H,-3.40306246
15,2.2776970872,-7.3603760482\H,-2.5773374897,4.5617600617,-6.87432422
74\H,-1.7512686658,5.1107846842,-4.6050011\H,-1.7442949745,3.397364543
3,-2.845096832\H,-1.6319473345,-1.1450502736,1.3381303254\H,0.08544905
75,-1.2433380812,3.1104716054\H,2.3125862185,-0.233654162,2.7252949316
\H,2.8146692248,0.8618731062,0.5657569826\H,1.1105051755,0.9604468625,

-1.1978552324\\Version=ES64L-G16RevB.01\State=1-A\HF=-1433.9688542\RMS
D=5.361e-09\RMSF=4.130e-06\Dipole=-0.52012,1.4630195,-0.8626257\Quadru
pole=-7.6530912,-2.8031862,10.4562774,14.9602272,5.1739333,0.8851864\P
G=C01 [X(C21H18O3S1)]\\@

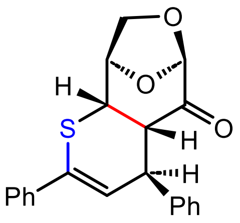

(exo,exo)-**3a**

HF=-1434.0278626 a.u., NIMAG=0
1\1\GINC-R02N09\FOpt\RPBE1PBE\def2TZVP\C21H18O3S1\WURTHWE\10-Mar-2022\
0\\# pbe1pbe/def2tzvp opt freq pop=nbo emp=gd3bj scrf=(solvent=thf)\\exo,exo-3a\\0,1\
C,0.3357112219,-0.0395434818,-0.004272967\C,0.3927412216,-0.309403215,1.3632952946\
C,1.4388385914,-1.094706639,1.8490953119\C,2.3898810493,-1.6145412247,0.9863024099\
C,2.3187133296,-1.3492620979,-0.3747017843\C,1.2897658252,-0.5586719028,-0.8658433673\
C,-0.6299761117,0.213898931,2.2910949993\C,-1.1397464571,-0.5242363408,3.2789535279\
C,-2.1696414719,-0.0893978036,4.2645678677\C,-3.5515773818,- 0.6132347791,3.92485866\
C,-4.4443792815,-0.9173254544,4.9498257345\C,-5.7330626766,-1.3422285761,4.6660523644\
C,-6.1497246684,-1.468353243,3.3473365083\C,-5.2671005414,-1.1710664312,2.3195993892\
C,-3.9760414536,-0.7490249761,2.6070470367\S,-1.0627943696,1.8796572877,1.9555795665\
C,-2.3566749584,2.2467232678,3.1766057691\C,-2.2274568809,1.4233052826,4.4621045999\
C,-1.128992132,1.9900312404,5.3347217827\C,-1.1724945466,3.5205275508,5.3998524834\
O,-0.9845101877,4.0084224813,4.1028629269\C,-2.244407265,3.7382904659,3.4947631951\
C,-3.2059051531,4.1687120997,4.5987816116\O,-2.4583194728,3.938797968,5.7956155639\
O,-0.3242596702,1.3391313202,5.9481174243\H,-4.1300017303,3.5870315283,4.6164690388\
H,-3.4472860809,5.230508628,4.5251621208\H,-2.3228325014,4.3457366582,2.5937148292\
H,-0.4102026048,3.9153144951,6.0721625537\H,-3.3289980419,2.0477890329,2.719623821\
H,-3.1405711167,1.6309368806,5.0359083843\H,-0.8124833925,-1.5563868064,3.351386527\
H,-1.8921315516,-0.5087729597,5.2367985629\H,-0.4726401283,0.5670352001,-0.3963020742\
H,1.225630767,-0.3468988598,-1.9269422106\H,3.0661805294,-1.7518558037,-1.0483429531\
H,3.1980567798,-2.2197223647,1.3807407647\H,1.5109716751,-1.2801529692,2.9143552004\
H,-4.1227035051,-0.8226066788,5.9824583838\H,-6.4125811095,-1.5806279054,5.4764371884\
H,-7.1558966943,-1.8032310518,3.1232890315\H,-5.5816574789,-1.2721147376,1.2870260942\
H,-3.2853454877,-0.5327296238,1.7995237982\\Version=ES64L-G16RevB.01\State=1-A\HF=-
1434.0278626\ RMSD=1.295e-09\RMSF=3.033e-06\Dipole=-1.6436537,0.1146738,-
0.7226229\Quadrupole= 3.6447943,-0.6692413,-2.975553,-7.5576178,-5.2100564,-
1.3987527\PG=C01 [X(C21H18O3S1)]\\@

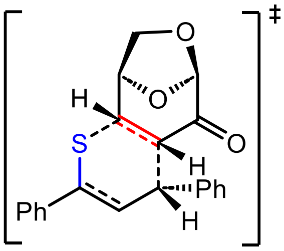

TS-*(exo,endo)*-**3a**

HF=-1433.9675644 a.u., NIMAG=1, -261.8788 cm-1

1\1\GINC-R08N35\FTS\RPBE1PBE\def2TZVP\C21H18O3S1\WURTHWE\06-Mar-2022\0\\#
pbe1pbe/def2tzvp Opt=(ts,noeigentest,readfc,maxstep=8) geom=check guess=read freq pop=nbo
emp=gd3bj scrf=(solvent=thf)\\TS-exo,endo-3a\\0,1\
C,-0.0647962064,-0.1376996743,0.1958708917\C,-0.0236056022,-0.0462744172,1.5899556054\
C,1.221212012,-0.0726659643,2.2230495534\C,2.3887106737,-0.1733356825,1.4869035225\
C,2.3343587164,-0.2636644607,0.1022074938\C,1.1033386739,-0.2506089148,-0.5390418951\
C,-1.2577425344,0 .0754082857,2.3817864862\C,-2.3815711555,0.7011444263,1.8321257533\
C,- 3.5663978295,0.8432591164,2.5147418821\C,-4.7730683768,1.4376823446,1.9891353602\
C,-4.9929291351,1.6161274483,0.6185755191\C,-6.1616949545,2.197586027,0.1653303007\
C,-7.1361122438,2.6042156582,1.068904378\C,-6.9382366435,2.4219646367,2.4316002381\
C,-5.7700526844,1.8381923176,2.8854786079\S,-1.3716418781,-0.6584289871,3.8961741264\
C,-2.965224912,-2.1927090997,3.3871031366\C,-4.1502217983,-1.5857232072,2.9682495209\
C,-4.6202731982,-1.7986216696,1.6217002163\C,-3.6835063194,-2.6386716159, 0.754534478\
O,-2.3474857473,-2.4093447338,1.088482365\C,-2.252629085,-3.0397815662,2.3591503866\
C,-3.0095950923,-4.3484652459,2.0961653077\O,-3.9298889629,-4.000196545,1.0662401727\
O,-5.7123858194,-1.4571186383,1.1927305979\H,-3.5438623244,-4.7156644253,2.9743545151\
H,-2.3320206793,-5.1214787122,1.7260130719\H,-1.203413702,-3.1901545843,2.5988236961\
H,-3.8476622011,-2.4610252279,-0.3086715139\H,-2.8987924501,-2.5405279176,4.4135049563\
H,-4.8523127621,-1.1831235775,3.6872788849\H,-2.3143038814,1.0507908356,0.808333306\
H,-3.5242529073,0.6898567979,3.5882518613\H,1.2620439921,-0.0005838693,3.3034415843\
H,3.3458524889,-0.1791055867,1.995270387\H,3.2479764136,-0.3478302133,-0.4747056185\
H,1.0513471482,-0.3387682181,-1.6179991223\H,-1.0188141022,-0.1659864951,-0.314 74742\
H,-5.6133693667,1.6902426093,3.9486434723\H,-7.6989445789,2.7318494528,3.1382237857\
H,-8.0531658263,3.056365754,0.7095418097\H,-6.3214541504,2.3272012144,-0.8985029838\
H,-4.2505616086,1.2848360826,-0.0971924432\\Version=ES64L-G16RevB.01\State=1-A\HF=-
1433.9675644\RMSD=4.112e-09\RMSF=1.660e-06\Dipole=1.0699917,0.4457851,0.3395548\
Quadrupole=-1.8393336,-2.6907989,4.5301325,-12.3135504,-6.9036781,-7.2481649\PG=C01
[X(C21H18O3S1)]\\@

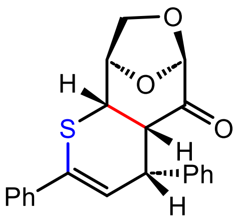

(exo,endo)-**3a**

HF=-1434.0215428 a.u., NIMAG=0
1\1\GINC-R03N27\FOpt\RPBE1PBE\def2TZVP\C21H18O3S1\WURTHWE\10-Mar-2022\ 0\\#
pbe1pbe/def2tzvp Opt Pop=NBO Freq emp=gd3bj scrf=(solvent=thf)\\exo,endo-3a X-Ray\\0,1\
S,1.2509426328,6.4598033369,3.4885530985\O,4.5540220868,4.3762122204,3.1857063949\
O,5.6077387346,7.36685786114.4843645266\O,4.0146842678,7.5545922107,2.9127620707\
C,3.4558123282,5.5907478433,4.979505715\H,4.1256144894,5.8905053206,5.7943123524\
C,3.8537336211,2.1370383804,5.1569488375\H,3.4019825099,2.0445795363,4.1773425579\
C,5.0639252207,2.3824666591,7.6379341896\H,5.5288363459,2.4845380866,8.6119408739\
C,1.6087068617,3.9400998359,4.5212135637\H,1.2965433229,2.9023880581,4.5679880226\
C,3.5904267734,3.2610122625,5.9317442984\C,5.3194296933,1.2648666002,6.8559139545\
H,5.987970107,0.4898771905,7.2127278423\C,4.3710374197,5.3990865729,3.7896602667\
C,4.2033140757,3.3672882432,7.1776828786\H,3.9968858173,4.2322302792,7.801276946\
C,-2.2644553545,4.4788541269,1.7198458084\H,-3.1654831336,5.0593377441,1.5582958466\
C,0.9534576192,4.7396073998,3.6773195145\C,-0.1063658562,4.2390864095,2.7797889413\
C,2.6725256099,4.3732854037,5.4735476511\H,2.1641480211,4.7258005819,6.3829202448\
C,4.7092873994,1.1458678396,5.6155264436\H,4.9036015916,0.27740876,4.9962732668\
C,0.043946486,3.0059005404,2.143632014\H,0.9599158505,2.4446388613,2.2861920032\
C,4.6173180472,8.2640313568,4.9923582472\H,4.9563813386,9.2921791849,4.8531058709\
H,4.4533455379,8.0772398589,6.0558014281\C,-0.9527816307,2.5108376496,1.3190015602\
H,-0.8178876508,1.5525601059,0.8306052892\C,-1.2696365723,4.9739871268,2.5489094429\
H,-1.402828364,5.9324161607,3.0375895633\C,5.0394524281,6.7166564125,3.3734250855\
H,5.7821566026,6.5569553121,2.5911839739\C,3.3965058696,7.959133933,4.1299816629\
H,2.7814666842,8.8345900018,3.9244805419\C,-2.1115580674,3.2450797691,1.1041070897\
H,-2.8884040988,2.8599001578,0.4539238473\C,2.5631996002,6.8094902771,4.6999404044\
H,2.096756734,7.1367185275,5.6316818685\\Version=ES64L-G16RevB.01\State=1-A\HF=-
1434.0215428\RMSD=4.804e-09\RMSF=4.661e-06\Dipole=-0.4724197,0.9699488,1.4795309\
Quadrupole=-5.9106054,7.7272331,-1.8166277,0.4 757868,7.0935883,8.8521273\PG=C01
[X(C21H18O3S1)]\\@

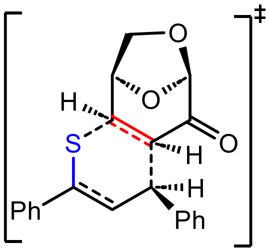

TS-*(endo,exo)*-**3a**

HF=-1433.9442878 a.u., NIMAG=1, -307.9778 cm-1
1\1\GINC-R08N40\FTS\RPBE1PBE\def2TZVP\C21H18O3S1\WURTHWE\14-Jul-2024\0\\#
pbe1pbe/def2tzvp Opt=(ts,noeigentest,maxstep=7,readfc) geom=check guess=read Pop=NBO Freq
emp=gd3bj scrf=(solvent=thf)\\TS-endo,exo-3a\\0,1\
C,0.3274467901,-0.839658599,0.3333839549\C,-0.1996431677,-0.3689344842,1.5194840559\
C,-0.0184094973,0.9604539988,1.8895398937\C,0.7184444818,1.8081511125,1.0764412475\
C,1.2561535181,1.3343558845,-0.1067823763\C,1.0459868528,0.0123372101,-0.5139414672\
C,1.7167474307,-0.4495982747,-1.7174650523\C,1.701735845,-1.6954972844,-2.3217324698\
C,0.6417201457,-2.5738558113,-2.5550512504\S,-0.9573566139,-2.0715189509,-2.4209752133\
C,-0.8119713789,-0.115832233,-3.7177403179\C,0.4122780333,0.5352923194,-3.6426856912\
C,0.4304977026,1.9389209252,-3.2649694561\C,-0.9533990024,2.551006995,-2.9887322454\
O,-1.9144138491,1.9822749816,-3.8274289976\C,-2.0243207603,0.6663761054,-3.2652233382\
C,-2.0703883639,1.0009928295,-1.7722855098\O,-1.3420979227,2.2244073844,-1.6751453224\
C,0.9220616534,-3.899338886,-3.145478077\C,1.9806499444,-4.1071096172,-4.0334344635\
C,2.21317745,-5.3611230034,-4.5746887302\C,1.4052 872089,-6.4346030739,-4.2279153642\
C,0.3528715259,-6.2420482647,-3.343031821\C,0.1096015547,-4.9862058939,-2.8146591877\
O,1.4297238963,2.6342397005,-3.2166902048\H,2.5709093457,0.1672136696,-1.9851205635\
H,2.6092074951,-3.2793839427,-4.3379777089\H,-0.7118649812,-4.8362471465,-2.1239776806\
H,-0.2811332306,-7.0747965023,-3.0616933335\H,1.5932437663,-7.416039516,-4.6472561502\
H,3.0275240351,-5.4975962431,-5.2767114312\H,2.6321590142,-1.955753098,-2.8200187009\
H,-0.9686310232,-0.8209206727,-4.5250925886\H,1.263750749,0.1818851403,-4.2081125897\,-
0.9228869471,3.6329586775,-3.1218241219\H,-2.9460104253,0.214936289,-3.6263211797\
H,-3.099521743,1.1758469275,-1.4512451653\H,-1.6045545551,0.2382639064,-1.1516611856\
H,-0.7501360083,-1.0416499175,2.166693473\H,-0.4380324247,1.3265158855,2.8194202627\
H,0.8782905339,2.8398054127,1.366045519\H,1.8408311019,1.9930954147,-0.7379480056\
H,0.1974635657,-1.877896549,0.0618768734\\Version=ES64L-G16RevB.01\State=1-A\HF=-
1433.9442878\RMSD=3.794e-09\RMSF=2.917e-06\Dipole=-0.0205662,-1.8800547,0.31621\
Quadrupole=1.006668,-4.5802435,3.5735755,-4.0937803,-5.6735962,8.9126959\PG= C01
[X(C21H18O3S1)]\\@

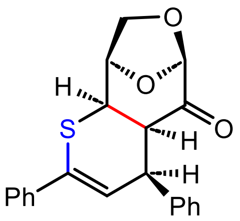

(endo,exo)-**3a**

HF=-1434.0116961 a.u., NIMAG=0

1\1\GINC-R08N11\FOpt\RPBE1PBE\def2TZVP\C21H18O3S1\WURTHWE\13-Jul-2024\ 0\\#
pbe1pbe/def2tzvp Opt=readfc geom=check guess=read Pop=NBO Freq emp=gd3bj
scrf=(solvent=thf)\\endo,exo-3a)\\0,1\
C,0.3632227517,-3.2792796773,-1.8443686114\C,1.289353239,-2.3072964056,-2.2288326971\
C,1.8346650907,-2.3805739416,-3.5107705887\C,1.4512860929,-3.3809893656,-4.3900421243\
C,0.5235864771,-4.3356415506,-3.9999569576\C,-0.0158747115,-4.2814630025,-2.7215525529\
C,1.6699975236,-1.2206429749,-1.3116159274\C,0.8690079181,-0.7251124985,-0.3633799483\
C,1.1988702616,0.4803380903,0.4750913494\H,0.5003471451,0.5001597253,1.3131650839\
S,3.2792691841,-0.5220759785,-1.5153716847\C,3.6749561301,-0.3529096802,0.2495439189\
C,2.606454306,0.3777390011,1.0934985286\C,3.0719999172,1.6557111821,1.7583999797\
C,4.5835848675,1.8666304899,1.7775507122\O,5.2353760762,0.6327286518,1.7732070002\
C,5.0872236272,0.2154761433,0.4151178289\C,5.3919907524,1.5109849666,-0.3216287585\
O,4.9337041093,2.5215066397,0.5752938593\O,2.321674031,2.4319167211,2.2917388777\
C,1.4658387047,3.6262569658,-1.7113614844\C,0.1261695901,3.906177588,-1.927780912\
C,-0.8403821479,3.0970523286,-1.3446557911\C,-0.4618346134,2.0176567779,-0.5642351979\
C,0.8833628151,1.7183073277,-0.3503237405\C,1.8398488576,2.5408880893,-0.9298597998\
H,-0.0471545217,-3.2634503272,-0.8417068625\H,2.5598119023,-1.6401926995,-3.8302825875\
H,1.8810047731,-3.4137008487,-5.384540572\H,0.22815251,-5.1221770832,-4.6843873192\
H,-0.72936512,-5.0317215713,-2.4005112788\H,-0.1192673015,-1.1538163471,-0.2381888153\
H,3.7159721505,-1.3799373832,0.6129223373\H,2.4701530502,-0.269563253,1.969731432\
H,4.8907147928,2.45670452,2.6420516851\H,5.8277590208,-0.5563228242,0.212099598\
H,6.4675764123,1.6235681098,-0.4729825174\H,4.8782243705,1.6036711517,-1.2797146445\
H,2.2317446378,4.2575062841,-2.1475773195\H,-0.1646388141,4.7540973161,-2.5370093503\
H,-1.8927448734,3.3107063584,-1.4936326491\H,-1.2238552588,1.393217044,-0.108602198\
H,2.8918682737,2.36242194,-0.7616143004\\Version=ES64L-G16RevB.01\State=1-A\HF=-
1434.0116961\RMSD=8.161e-09\RMSF=1.493e-06\Dipole=0.2171083,-1.5784566,-0.4440955\
Quadrupole=7.2749744,-4.2940909,-2.9808835,4.0605705,-1.4020403,-6.794707\PG=C01
[X(C21H18O3S1)]\\@

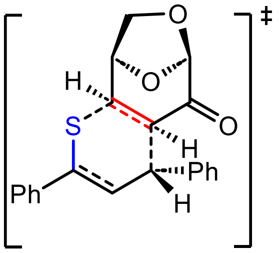

TS-*(endo,endo)*-**3a**

HF=-1433.9619482 a.u., NIMAG=1, -274.5877 cm-1

1\1\GINC-R08N10\FTS\RPBE1PBE\def2TZVP\C21H18O3S1\WURTHWE\14-Jul-2024\0\\# pbe1pbe/def2tzvp Opt=(ts,noeigentest,maxstep=7,readfc) geom=check guess=read Pop=NBO Freq emp=gd3bj scrf=(solvent=thf)\\TS-endo,endo-3a\\0,1\ O,-0.9795920724,-1.106141514,1.3308493215\ C,-0.0087500754,-0.6223934407,2.2439885039\ O,1.104925043,-0.2628055087,1.4806210018\C,1.1877355476,-1.3652752072,0.5655449821\ C,-0.273429457,-1.4420064574,0.1383498188\C,1.7162295296,-2.5665450778,1.3251732403\ C,1.2342895034,-2.7626129707,2.614800825\C,0.3763242166,-1.7526634619,3.2046909982\ C,-0.5581463164,-4.4708180165,2.3333679222\C,0.3117885479,-5.5188885666,2.1283180035\ C,1.1654871555,-5.6109732511,1.0251354932\S,1.2538150611,-4.3596571972,-0.0959157795\ C,2.0731207273,-6.766134431,0.9024505108\C,2.5813842761,-7.4076106488,2.0355282628\ C,3.4414320291,-8.4865247903,1.9110367995\C,3.7989962663,-8.9534794297,0.6543305133 \ C,3.2978961258,-8.3267075336,-0.4793235087\C,2.4495594967,-7.2406463841,-0.3561482947\ C,-1.4542287475,-4.3476477973,3.4616157029\C,-1.2784900741,-5.065921722,4.6503491469\ C,-2.1782330629,-4.9317386949,5.6895739987\C,-3.269049132,-4.0775542901,5.5648403776\ C,-3.4480200444,-3.3482024836,4.3981093881\C,-2.5427366907,-3.4750525076,3.3592857657\ O,0.0197846935,-1.7333952243,4.3683004316\H,2.3326821544,-7.0408783687,3.0239470055\ H,2.0567979311,-6.7528779946,-1.2404822327\H,3.5679307274,-8.6882721255,-1.4646685288\ H,4.4672713319,-9.8011904486,0.5580225016\H,3.839005597,-8.9602215892,2.801027647\ H,0.368390798,-6.3046270692,2.8728274838\H,-0.7621313199,-3.8268202282,1.485395865\ H,-0.4253981318,-5.7235491769,4.7662946193\H,-2.0299012551,-5.4901963284,6.6062676222\ H,-3.9725953324,-3.9752994428,6.3828778426\H,-4.2899261189,-2.672962424,4.3017523699\ H,-2.6634475564,-2.8913527222,2.4536391302\H,-0.3876840663,0.2416530227,2.7911419415\ H,1.8535811858,-1.0854426763,-0.2481457844\H,-0.5802489968,-2.4262757132,-0.2093040924\ H,-0.4738721975,-0.692482219,-0.6316219677\H,2.7422937598,-2.8474919633,1.119187618\ H,1.7356806407,-3.4355844783,3.2980891059\\ Version=ES64L-G16RevB.01\State=1-A\HF=-1433.9619482\RMSD=7.284e-09\RMSF=9.976e-06\Dipole=0.3484755,-1.4946494,-0.5467296\Quadrupole=2.560432,-2.8709536,0.3105216,-7.1386935,-4.0276783,-9.9676503\PG=C01 [X(C21H18O3S1)]\\@

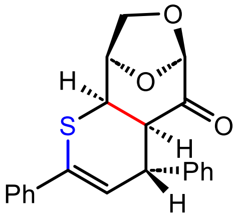

(endo,endo)-**3a**

HF=-1434.0176193 a.u., NIMAG=0

1\1\GINC-R08N18\FOpt\RPBE1PBE\def2TZVP\C21H18O3S1\WURTHWE\19-Jul-2024\0\\#
pbe1pbe/def2tzvp Opt Pop=NBO Freq emp=gd3bj scrf=(solvent=thf)\\endo,endo-3a)\\0,1\
C,-2.1197643348,1.2110055276,0.5080915462\C,-1.6256210785,0.0741121935,-0.1357172864\
C,-2.5387309681,-0.8201388101,-0.6947689274\C,-3.9019461861,-0.5773083863,-0.6319133716\
C,-4.3800895063,0.5598457298,0.0021074228\C,-3.4817335399,1.4509199226,0.5745236594\
C,-0.177379734,-0.1689275403,-0.2362480581\C,0.7517533782,0.7920638417,-0.2251072552\
C,2.2126192169,0.5573432236,-0.4693234169\C,3.0199130915,1.8081335533,-0.2188600166\
C,2.9913724871,2.4186120631,1.0328301762\C,3.7198204556,3.5725727125,1.2763103897\
C,4.4888690448,4.1350125654,0.2660742093\C,4.5220591926,3.5350147713,-0.984036576\
C,3.7922529223,2.3784270224,-1.2227260471\S,0.3639327039,-1.8442234119,-0.3842269017\
C,1.7641676851,-1.6856443431,0.7609553352\C,2.7945965188,-0.6428342184,0.3045706271\
C,3.9436180153,-1.2933306078,-0.426786672\C,4.3667128417,-2.6440715494,0.1615623716\
O,3.769382568,-2.8356051377,1.4084043424\C,2.4065982881,-3.05559413,1.0408187173\
C,2.573927236,-3.9922678518,-0.1548019548\O,3.8507738466,-3.6444935506,-0.6877703792\
H,-1.4327988892,1.9034270818,0.9798537815\H,-2.1798551257,-1.7137579665,-1.1934440648\
H,-4.5926308712,-1.2819709551,-1.0804461432\H,-5.4459392718,0.7475936312,0.0574573038\
H,-3.8445768574,2.3347953565,1.0865173905\H,0.4379809466,1.8250265124,-0.1181794481\
H,2.3161498443,0.3010650226,-1.5320169133\H,2.3874359993,1.9883643823,1.82644334\
H,3.6860055463,4.0352812786,2.2561136264\H,5.0593700652,5.0372993516,0.4537351461\
H,5.1201231974,3.9668590105,-1.7784400498\H,3.8292019113,1.904222732,-2.1968526148\
H,5.4512609657,-2.7295354867,0.2393980231\H,1.9014192157,-3.5429638012,1.8719355159\
H,1.8068051447,-3.872052514,-0.9210970429\H,2.5985050261,-5.0322963809,0.1761857431\
H,1.3235895476,-1.3274478383,1.6917044976\H,3.255300159,-0.2688796687,1.2283416267\
O, 4.4910813013,-0.8390563373,-1.3970226519\\Version=ES64L-G16RevB.01\State=1-A\HF=-
1434.0176193\RMSD=5.979e-09\RMSF=2.714e-06\Dipole=-1.0287141,0.2249258,1.3580983\
Quadrupole=-1.6760102,5.2789119,-3.6029018,4.444166,3.9313516,0.0843659\PG=C01
[X(C21H18O3S1)]\\@

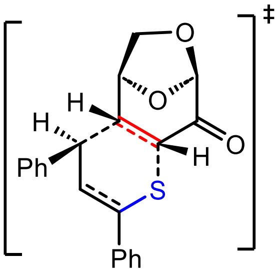

TS-*(exo,exo)*-**4a**

HF=-1433.9656043 a.u., NIMAG=1, -338.3135 cm-1

1\1\GINC-R01N03\FTS\RPBE1PBE\def2TZVP\C21H18O3S1\WURTHWE\25-Oct-2024\0\\#
pbe1pbe/def2tzvp opt=(ts,noeigentest,readfc,maxstep=12,maxcycle=200) geom=check guess=read
freq pop=nbo emp=gd3bj scrf=(solvent=thf)\\TS-exo,exo-4a\\0,1\
C,-0.2035851963,0.5542825267,-0.0027743675\S,0.0707524071,0.389754263,2.4052292722\
C,1.7338193835,0.2395452704,2.4470976828\C,2.5326448576,1.1674298194,1.7653037514\
H,3.5873182598,0.9355915365,1.6699802595\C,2.0133358229,2.239795187,1.0679959363\
H,1.0156132911,2.5610339483,1.3521900891\C,0.8973995002,1.1542345009,-0.5942280025\
H,0.8034585999,2.1396090125,-1.0325870502\C,1.9733236821,0.2501018941,-1.1325658226\
O,2.0464460049,-0.9025612334,-0.3397994462\C,0.8715568749,-1.5944850787,-0.7279949949\
H,0.9224833161,-2.6291872988,-0.3962225719\C,-0.3619042869,-0.9013934559,-0.1787998495\
O,-1.4147017031,-1.4907388574,-0.053773975\H,-1.1213526051,1.1120709383,0.1369206536\
C,0.9038483498,-1.4209598363,-2.2553203013\O,1.6297477174,-0.2098417895,-2.4330153653\
H,1.4501213823,-2.2396052293,-2.7284243597\H,-0.0931228191,-1.3423366587,-2.6939474485\
H,2.9513349731,0.728422164,-1.1820102315\C,2.8169157867,3.2063982024,0.3263708722\
C,4.1349942294,2.9572259975,-0.0679955568\C,4.8480576279,3.905321511,-0.7809661888\
C,4.2612109606,5.1193878949,-1.1139350528\C,2.9523742279,5.3795830761,-0.7309849334\
C,2.2374400547,4.4297138074,-0.0224609357\H,4.6120539901,2.01684072,0.1814142311\
H,5.8684830765,3.6960429026,-1.0796683698\H,4.8226800831,5.8594114503,-1.6719342886\
H,2.4877808175,6.3245038346,-0.9869341241\H,1.2135214342,4.6318376682,0.2740484342\
C,2.3786011491,-0.9429826301,3.0511471547\C,3.6416199101,-0.8303248378,3.6349899383\
C,4.2611287827,-1.9346767496,4.1982481004\C,3.6343148961,-3.1722831212,4.1766745749\
C,2.3770020476,-3.294326162,3.5985479166\C,1.7508405988,-2.1885931327,3.0502408845\
H,4.1334666351,0.1350562964,3.6723257338\H,5.2358996073,-1.8259986715,4.6595712693\
H,4.1204315033,-4.0372776691,4.6126325084\H,1.880885299,-4.2578001547,3.5758649603\
H,0.7662390616,-2.2792623238,2.6072819372\\Version=ES64L-G16RevB.01\State=1-A\HF=-
1433.9656043\RMSD=3.648e-09\RMSF=2.203e-06\Dipole=1.9934321,1.2930915,-
0.6909871\Quadrupole=-6.3611371,12.516807,-6.1556699,-4.9034352,3.9392933,-
5.7973049\PG=C01 [X(C21H18O3S1)]\\@

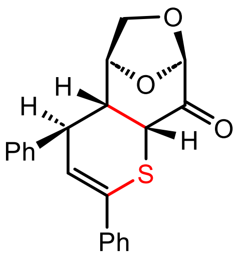

(exo,exo)-**4a**

HF=-1434.0233886 a.u., NIMAG=0

1\1\GINC-R01N18\FOpt\RPBE1PBE\def2TZVP\C21H18O3S1\WURTHWE\20-Jul-2024\ 0\\# pbe1pbe/def2tzvp opt=readfc geom=check guess=read freq pop=nbo emp=gd3bj scrf=(solvent=thf)\\exo,exo-4a\\0,1\
C,0.8712332987,1.4444257875,0.8663276973\C,2.0779143046,0.4955455242,0.7451279599\
C,0.3682981614,2.0186550534,-0.4635232218\C,1.3860915527,2.0372469054,-1.5852179867\
O,2.0322874657,0.7763952383,-1.6410916117\C,2.8686845242,0.8190000877,-0.5138423144\
O,-0.7289663016,2.4895490173,-0.6072304293\S,-0.513070635,0.7316294154,1.7913842142\
C,1.638818144,-0.9978255937,0.8438475478\C,0.2950303967,-1.2653997573,0.2465967414\
C,-0.7899320123,-0.6125513748,0.6817783211\C,-2.5458087336,-2.2361979645,0.0172369681\
C,-2.1682289807,-0.9157646998,0.2723188154\C,-3.1171369052,0.0953979045,0.1169251903\
C,-4.4026918066,-0.2051242012,-0.3045099201\C,-4.764353072,-1.5188408078,-0.5693665689\
C,-3.8306225645,-2.5336942033,-0.4057232987\C,3.8405835784,-3.248124334,-1.3020913849\
C,2.8212723058,-2.3892922511,-0.9185477124\C,2.7163311967,-1.9529851759,0.4001005067\
C,3.6581453768,-2.3980085876,1.3232268164\C,4.6807818992,-3.2549893243,0.943181718\
C,4.7747688401,-3.6839102365,-0.3728266837\O,3.3807528143,2.139716346,-0.447332416\
C,2.5475881626,2.9645904011,-1.2483546423\H,1.1757346967,2.3255793297,1.4428005155\
H,2.750256462,0.6801312529,1.5871751989\H,0.8962652764,2.2464395354,-2.5338573128\
H,3.6921968962,0.1215620757,-0.6644454345\H,1.5044162984,-1.1396347442,1.9244429057\
H,0.1911220796,-2.0267649687,-0.51835011\H,-1.8276855411,-3.0334502113,0.1712749986\
H,-2.8263105578,1.1209751723,0.3101849844\H,-5.1257776221,0.5927363114,-0.4300173973\
H,-5.771750437,-1.7526433227,-0.8938428491\H,-4.108683421,-3.5641664427,-0.5953453639\
H,3.905893341,-3.5764493464,-2.3331685337\H,2.1095053911,-2.0423054249,-1.659158833\
H,3.584663232,-2.0722744487,2.356067416\H,5.4016491084,-3.5928551235,1.6789327289\
H,5.5702128311,-4.3562333437,-0.6726323979\H,3.0739364153,3.2677232221,-2.1573525259\
H,2.2488745406,3.8527893082,-0.6872562956\\Version=ES64L-G16RevB.01\State=1-A\HF=-1434.0233886\RMSD=7.679e-09\RMSF=1.278e-06\Dipole=1.131436,-0.2209912,-0.2844535\
Quadrupole=-0.9656277,3.1754417,-2.209814,2.357629,3.5474642,-0.1456607\PG=C01 [X(C21H18O3S1)]\\@

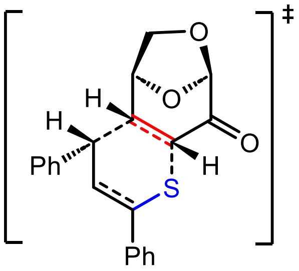

TS-*(exo,endo)*-**4a**

HF=-1433.9505696 a.u., NIMAG=1, -314.7305 cm-1

1\1\GINC-R08N36\FTS\RPBE1PBE\def2TZVP\C21H18O3S1\WURTHWE\11-Sep-2024\0\\# pbe1pbe /def2tzvp opt=(ts,noeigentest,maxstep=15,readfc) geom=check guess=read freq pop=nbo emp=gd3bj scrf=(solvent=thf)\\TS-exo-endo-4a)\\0,1\
C,0.0017937813,-0.0085614111,-0.0023328315\C,-0.0040926545,-0.0028997493,1.3944032929\
C,1.2218191041,-0.0105980006,2.064752372\C,2.4126027807,-0.022691189,1.3577852322\
C,2.403801027,-0.040938472,-0.0301696454\C,1.1923510403,-0.035188228,-0.7080122635\
C,-1.2919071687,0.0071823056,2.114055812\C,-1.3808630552,-0.5958884551,3.3711955175\
C,-2.4732392069,-0.7384578841,4.2058988494\C,-3.8535219136,-1.0870117997,3.8989160671\
C,-4.7919416142,-1.0550950487,4.9367036435\C,-6.1041176979,-1.4354955039,4.7210572454\
C,-6.4953871871,-1.8871745314,3.4673959225\C,-5.5632321878,-1.9658227625,2.4394487216\
C,-4.2557417529,-1.5689665089,2.6511029677\S,-2.5512042698,0.9644888161,1.5390351043\
C,-2.6848661195,2.4097483558,3.325482976\C,-2.8723491045,1.7320749769,4.5274489786\
C,-1.8494249095,1.7987830393,5.5485569277\C,-0.608479301,2.5906442139,5.1401020101\
O,-0.3390451813,2.4221865257,3.7810691401\C,-1.3888339697,3.1690809555,3.1786961758\
C,-1.3880132923,4.4314262151,4.0504704415\O,-0.9132081917,3.9649772734,5.3098945881\
O,-1.959021479,1.3646977182,6.6852619074\H,-2.3789555073,4.8751607886,4.161016763\
H,-0.6913461916,5.1747661918,3.6556900401\H,-1.1315845023,3.3621176432,2.1408813681\
H,0.2618766981,2.3202524542,5.738761928\H,-3.5506577932,2.8420867531,2.8348750968\
H,-3.8580576901,1.4036595873,4.8239912789\H,-0.4286855322,-0.8373410567,3.835652894\
H,-2.2216522393,-0.8944645751,5.2517685827\H,-0.9443776275,-0.0048079334,-0.5306161493\
H,1.1744156962,-0.0531506501,-1.791523963\H,3.3371487317,-0.0554355516,-0.580822118\
H,3.353710535,-0.0078924082,1.8951944289\H,1.2500325146,0.041605446,3.1454014923\
H,-4.480380011,-0.716339527,5.919392184\H,-6.8201588418,-1.3908184858,5.5330511922\
H,-7.520790437,-2.1931536127,3.2953698552\H,-5.8588879177,-2.3428704645,1.467392642\
H,-3.5314324751,-1.6481210581,1.8519883011\\Version=ES64L-G16RevB.01\State=1-A\HF=-1433.9505696\RMSD=3.574e-09\RMSF=9.920e-07\Dipole=-0.3443424,-0.231971,-1.6551711\
Quadrupole=13.0681735,-0.1606851,-12.9074884,0.5124802,-3.5159808,-5.3148456\PG=C01 [X(C21H18O3S1)]\\@

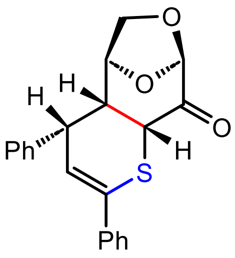

(exo,endo)-**4a**

HF=-1434.027101 a.u., NIMAG=0

1\1\GINC-R03N44\FOpt\RPBE1PBE\def2TZVP\C21H18O3S1\WURTHWE\21-Jul-2024\ 0\\#
pbe1pbe/def2tzvp opt=readfc geom=check guess=read freq pop=nbo emp=gd3bj
scrf=(solvent=thf)\\exo,endo-4a)\\0,1\
C,0.04064715,1.7580499997,-1.6856007602\C,0.8040703765,0.9601086087,-0.6192371454\
C,0.8369002199,1.8519824857,-2.9785160359\C,2.3058845314,2.1744667148,-2.7186234503\
O,2.8331083718,1.1485581982,-1.8836274208\C,2.2536289318,1.4497642402,-0.6375703511\
O,0.3899934709,1.7174485267,-4.0855175646\S,-1.6865137258,1.3156765859,-1.8580552213\
C,0.7735813688,-0.5587700979,-0.8382846788\C,-0.5648000566,-1.1017560805,-1.2276812173\
C,-1.6354190611,-0.4245269421,-1.6473200792\C,-3.3779145653,-2.1298213236,-1.1510414564\
C,-2.9141880522,-1.092007332,-1.9610136233\C,-3.6793035299,-0.7086614099,-3.0630074411\
C,-4.8664638376,-1.360908108,-3.3580883007\C,-5.3118585295,-2.4004889592,-2.5542692184\
C,-4.5630275941,-2.7821070795,-1.4489611278\H,1.4678457039,-0.7851184496,-1.6565038956\
O,2.3160637338,2.8571494439,-0.511885953\C,2.4820638871,3.3936939591,-1.8217918541\
C,0.5331727971,-1.311939931,1.5561947058\C,1.0084593646,-1.9520585582,2.6896247017\
C,2.2542021361,-2.5667622804,2.6723477151\C,3.0170749962,-2.5375235724,1.5147856953\
C,2.5375199982,-1.8943565984,0.3811837595\C,1.2940650182,-1.2731272202,0.3901062198\
H,0.0024464667,2.7900409241,-1.3168779633\H,0.3673513157,1.1959830673,0.3543807736\
H,2.860398233,2.2085675819,-3.6538658545\H,2.8517299102,0.9823844598,0.1458059892\
H,-0.6504011419,-2.1835076962,-1.1852270034\H,-2.8078732426,-2.4119854398,-0.2733725534\
H,-3.3283858786,0.0934301014,-3.7019432479\H,-5.4437207446,-1.0573510937,-4.2238443825\
H,-6.2418517358,-2.9074563432,-2.7839052244\H,-4.9100337644,-3.5843358071,-0.8078253885\
H,3.4881223389,3.8056718314,-1.9315448739\H,1.7463616254,4.1812805522,-1.9990559931\
H,-0.4449058602,-0.8413562739,1.571182879\H,0.404188919,-1.9746013616,3.5894133978\
H,2.6259303179,-3.0691122588,3.5578793134\H,3.9887301721,-3.0175959938,1.4909953842\
H,3.1366549648,-1.87365507,-0.5234682543\\Version=ES64L-G16RevB.01\State=1-A\HF=-
1434.027101\RMSD=2.097e-09\RMSF=2.264e-06\Dipole=0.7002287,0.0671659,0.89958\
Quadrupole=3.2883007,3.401052,-6.6893527,6.0962625,-0.711367,-5.21785\PG=C01
[X(C21H18O3S1)]\\@

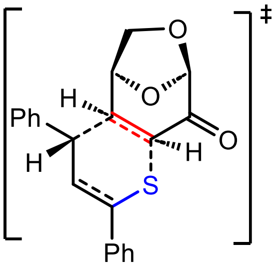

TS-*(endo,exo)*-**4a**

HF=-1433.9520172 a.u., NIMAG=1, -387.8642 cm-1

1\1\GINC-R02N22\FTS\RPBE1PBE\def2TZVP\C21H18O3S1\WURTHWE\20-Jul-2024\0\\# pbe1pbe/def2tzvp Opt=(ts,noeigentest,readfc) geom=check guess=read freq pop=nbo emp=gd3bj scrf=(solvent=thf)\\TS-endo-exo-4a\\0,1\
C,0.1897727483,-0.4494509095,-0.1745162444\C,0.2842321413,-0.3345417313,1.2008717005\
C,1.4643354139,0.1071876092,1.7873663973\C,2.5492494118,0.4373412003,0.9875655346\
C,2.4534141909,0.3284119396,-0.3897683918\C,1.2763862757,-0.1242023136,-0.9914242112\
C,1.2103071262,-0.2224014464,-2.444878858\C,0.2530029166,1.8103392852,-3.0002454973\
C,-0.0667428354,1.7544703471,-4.3494684253\S,1.161734837,-0.0056576662,-5.426194407\
C,0.372824318,-1.1814695959,-4.5288876923\C,0.4261813749,-1.1299999362,-3.1292236514\
C,1.2348405031,2.8653979601,-2.5687453063\O,0.8447208979,4.1069915647,-3.1932871721\
C,1.2691647387,3.9028196857,-4.5038480674\C,0.3578144338,2.8885952405,-5.2062523553\
C,2.6546437185,2.7463392301,-3.1303014749\O,2.5588306227,3.3331778274,-4.4236195118\
O,-0.0160129639,3.0559651693,-6.3427436553\C,-0.5157664162,-2.1670113866,-5.1752319228\
C,-0.7004196845,-3.4262760311,-4.5994185842\C,-1.5338680991,-4.3600604106,-5.1934746403\
C,-2.2060707189,-4.0483138311,-6.3669006779\C,-2.0291471003,-2.7990695281,-6.9479014826\
C,-1.1851334969,-1.8706381898,-6.3634661658\H,-0.9840968824,1.2746953257,-4.6687236261\
H,-0.4718547778,1.4777889173,-2.2718103719\H,1.2923993539,4.8461368045,-5.0506060918\
H,1.2216368391,3.0105372823,-1.4913518078\H,3.027259109,1.7301003994,-3.2337259384\
H,3.346696023,3.3274266642,-2.5159516081\H,2.0478309716,0.1732560119,-3.0075904891\
H,-0.261844384,-1.7585961613,-2.5734877117\H,-0.1649818836,-3.6872702216,-3.6937483686\
H,-1.038759942,-0.8984035877,-6.8187959494\H,-2.5515530407,-2.5470668404,-7.8635583872\
H,-2.860803384,-4.7773171369,-6.8300008986\H,-1.653989364,-5.3373848788,-4.7404935162\
H,-0.5683189978,-0.5857200071,1.8209631043\H,1.5349107607,0.1974004644,2.8648937402\
H,3.4726566807,0.782257825,1.437703145\H,3.3053712998,0.582917472,-1.0109278196\
H,-0.7416150549,-0.7792154128,-0.6208498837\\Version=ES64L-G16RevB.01\State=1-A\HF=-1433.9520172\RMSD=2.534e-09\RMSF=2.217e-06\Dipole=-0.282047,-1.3168251,3.1380268\
Quadrupole=3.0863914,-3.8477231,0.7613318,7.8176483,6.3942263,7.9490331\PG=C01
[X(C21H18O3S1)]\\@

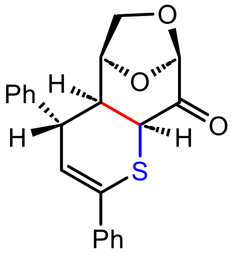

(endo,exo)-**4a**

HF=-1434.0227392, NIMAG=0
1\1\GINC-R08N18\FOpt\RPBE1PBE\def2TZVP\C21H18O3S1\WURTHWE\19-Jul-2024\0\\#
pbe1pbe/def2tzvp opt=readfc geom=check guess=read freq pop=nbo emp=gd3bj
scrf=(solvent=thf)\\endo,exo-4a\\0,1\
C,0.0346916807,1.3637703143,1.1398146508\C,-1.1690595655,0.7990006829,1.260646265\
S,-1.5229089144,-0.8781354106,0.906122359\C,1.2703939007,0.6829370832,0.6530262134\
C,1.3353692742,-0.8135167218,0.9958672275\C,0.1135470671,-1.6161406795,0.5441510404\
C,0.0577428691,-2.9830252836,1.2092409757\C,0.7025427443,-3.067811434,2.594877486\
O,1.9928140017,-2.5377018926,2.4992381827\C,1.7287818132,-1.1301677819,2.4363473099\
C,0.6586170967,-0.9896070537,3.5132119377\O,0.008280146,-2.2640648217,3.5111386496\
O,-0.4452344329,-3.9387339772,0.6761332447\C,-3.6828127679,3.5692876623,1.6563362344\
C,-2.5875794842,2.8393062886,1.2240409782\C,-2.3589203027,1.5508633056,1.7079542625\
C,-3.2638536103,1.0017566253,2.6168475236\C,-4.3594748704,1.7339781059,3.0468140614\
C,-4.5721252024,3.0196121828,2.5698599995\C,3.0230416117,1.1001950119,-2.6906107782\
C,1.9581030598,1.1315584833,-3.5809832596\C,0.660831524,1.0404782238,-3.0988753926\
C,0.4273188528,0.9194560596,-1.7357015756\C,1.4881171212,0.8791440039,-0.8370110131\
C,2.7869730351,0.9735277234,-1.3305490192\H,0.1250520184,2.4111994712,1.4080834644\
H,2.1667736665,-1.2205264592,0.4104868281\H,0.1445918871,-1.7691982411,-0.535203422\
H,0.7381025744,-4.1035306631,2.9355700655\H,2.6496701562,-0.6065076287,2.6932299129\
H,1.1128448749,-0.8290215311,4.4932661229\H,-0.0636475541,-0.2018082739,3.3091036469\
H,-3.8490691709,4.5678074843,1.2688021037\H,-1.9071700586,3.2596142243,0.4925793963\
H,-3.0973843331,0.0015286233,3.0000560012\H,-5.048588499,1.2977468053,3.7606100408\
H,-5.4316805788,3.5891543457,2.9034813586\H,4.040688717,1.1784487622,-3.0560244775\
H,2.1394688342,1.2316234097,-4.6448221929\H,-0.1773289425,1.0675208167,-3.785887282\
H,-0.5904836185,0.8609540055,-1.3673286511\H,3.6231007803,0.9505355008,-0.6383765523\
H,2.125491599,1.148371648,1.1544720726\\Version=ES64L-G16RevB.01\State=1-A\HF=-
1434.0227392\RMSD=2.322e-09\RMSF=1.396e-06\Dipole=1.0302424,2.1367528, 0.1654288\
Quadrupole=7.8653713,-11.2205394,3.3551681,-4.0579264,-2.9568236,-0.1648221\PG=C01
[X(C21H18O3S1)]\\@

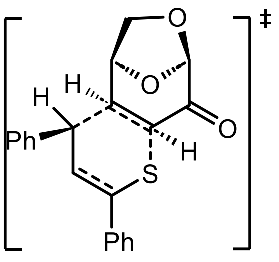

TS-*(endo,endo)*-**4a**

HF=-1433.953573 a.u., NIMAG=1, -377.2272 cm-1

1\1\GINC-R01N40\FTS\RPBE1PBE\def2TZVP\C21H18O3S1\WURTHWE\19-Jul-2024\0\\#
pbe1pbe/def2tzvp Opt=(ts,noeigentest,readfc) geom=check guess=read freq pop=nbo emp=gd3bj
scrf=(solvent=thf)\\TS-endo,endo-4a\\0,1\
C,-0.4688577072,-0.5491493797,0.117974866\C,-0.2761504937,-0.1286982175,1.4339618165\
C,0.9668910619,0.3929845093,1.7979383451\C,1.9906967352,0.4910330855,0.8691129287\
C,1.7844829908,0.0815446577,-0.4404855381\C,0.5498325766,-0.4359134351,-0.8119988898\
C,-1.355169045,-0.2262667391,2.4375869807\C,-1.5026511132,0.8130564461,3.3655772026\
C,-2.5456285322,0.8844142114,4.2677284767\C,-4.4120260056,1.0359602793,2.9014179263\
C,-4.5317938906,-0.2415052341,2.3658081078\S,-2.4845295523,-1.4566364186,2.3702185871\
C,-4.3641236004,2.2022768892,1.951321423\O,-5.3604675953,1.9707426036,0.9290936621\
C,-4.743126112,0.9807370379,0.163988154\C,-4.8201605908,-0.3537470878,0.9153184273\
C,-3.1419182239,2.3234970786,1.0504363946\O,-3.3849779501,1.3521690928,0.0378360577\
C,-2.7421970136,1.9862133422,5.2037725013\C,-3.5012417709,1.7623141628,6.355164363\
C,-3.7197027276,2.7779109925,7.2708627733\C,-3.1912940704,4.0410565844,7.0446985993\
C,-2.4440304612,4.2805101868,5.8975666339\C,-2.2212754038,3.2648810572,4.9850774678\
O,-5.1833357042,-1.3704345363,0.3721739032\H,-0.8361609255,1.6636866526,3.2687897437\
H,-3.0231044042,-0.0567978187,4.5222558742\H,-4.8440700424,1.2264711939,3.8740531612\
H,-4.9630728603,-1.0299520857,2.9707703308\H,-5.2226504595,0.9017714756,-0.8128806375\
H,-4.6095931433,3.1347583226,2.4546072594\H,-2.1945999763,2.1064580331,1.5327185682\
H,-3.1140299299,3.3226787771,0.6073381238\H,2.9545885391,0.8834270154,1.1719812027\
H,1.1430837518,0.6974769814,2.8235002215\H,-1.4337605543,-0.9490518321,-0.1675747701\
H,0.3794787274,-0.7527376602,-1.8344669147\H,2.5834697771,0.1614607449,-1.168397943\
H,-3.9156365515,0.7751257213,6.5309821368\H,-1.6478877276,3.4732393433,4.0893935831\
H,-2.0356299155,5.2672170291,5.7129887845\H,-3.3639103685,4.8391645494,7.7570743804\
H,-4.3049499563,2.5839728293,8.162017763\\Version=ES64L-G16RevB.01\State=1-A\HF=-
1433.953573\RMSD=2.706e-09\RMSF=3.201e-06\Dipole=0.8563786,2.5686203,2.0608607\
Quadrupole=-0.798645,-5.2094686,6.0081136,-1.3863585,-12.0968737,0.2474141\PG=C01
[X(C21H18O3S1)]\\@

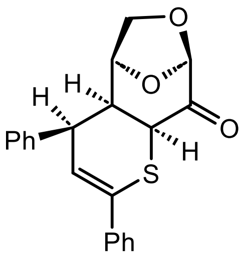

(endo,endo)-**4a**

HF=-1434.0210498 a.u., NIMAG=0

1\1\GINC-R08N03\FOpt\RPBE1PBE\def2TZVP\C21H18O3S1\WURTHWE\19-Jul-2024\0\\#
pbe1pbe/def2tzvp Opt=readfc geom=check guess=read freq pop=nbo emp=gd3bj
scrf=(solvent=thf)\\endo,endo-4a\\0,1\
C,0.2915315999,0.368608965,-0.9205503999\C,-0.5700283496,-0.2908768589,-1.6983104083\
S,-0.1066264968,-1.2825240496,-3.0629043538\C,1.7721414465,0.4021791616,-1.1172455132\
C,2.3565901998,-0.9290614569,-1.634121548\C,1.7202555069,-1.3912301182,-2.9478538623\
C,2.0252941085,-2.8498481237,-3.2530305662\C,2.2675551467,-3.7396593519,-2.0339519652\
O,3.2299094586,-3.1100552099,-1.2394655923\C,2.4676870758,-2.0648186224,-0.620070839\
C,1.1927143025,-2.8169143769,-0.2562159296\O,1.1053501948,-3.8380037436,-1.2547343463\
O,2.0971127911,-3.268304469,-4.3803411105\C,-4.1727701421,-1.3551688437,-1.3425032093\
C,-2.8091558978,-1.4034444121,-1.5866426564\C,-2.0297985194,-0.2509163722,-1.4803131921\
C,-2.6509555489,0.9529554261,-1.1456066886\C,-4.013627811,0.9979510798,-0.9004577508\
C,-4.7795655065,-0.156034581,-0.9971549263\H,1.9844406279,1.1298833577,-1.912691628\
C,3.7094076521,1.5805084005,-0.0457953994\C,2.5249418177,0.8657573267,0.107022724\
C,2.0953862818,0.5587196649,1.3949188335\C,2.831734644,0.9551434695,2.5021921402\
C,4.0112087697,1.6664979754,2.337415222\C,4.4484875581,1.9789589111,1.057743184\
H,-0.1175484137,0.9326214679,-0.0893253774\H,3.3997595425,-0.7137130358,-1.8899742013\
H,2.105149456,-0.796647893,-3.7765988234\H,2.6129204769,-4.7270345562,-2.3439327227\
H,3.0181467095,-1.7310699869,0.2573876783\H,1.2890062312,-3.2899773161,0.7236104881\
H,0.2981617838,-2.1981510346,-0.2796894645\H,-4.7629150628,-2.2609853065,-1.419689986\
H,-2.3384664367,-2.3447503562,-1.8458011099\H,-2.0588535725,1.8591842916,-1.0937081725\
H,-4.4816673534,1.9414971971,-0.6444298484\H,-5.8465444589,-0.1189107458,-0.8108872714\
H,4.0536739433,1.8325462373,-1.0440795608\H,1.1763735619,0.0004320092,1.5388935386\
H,2.4811057135,0.7064418371,3.4973143906\H,4.585420093,1.9788411697,3.2018459417\
H,5.3655668762,2.539680873,0.9178582827\\Version=ES64L-G16RevB.01\State=1-A\HF=-
1434.0210498\RMSD=9.360e-09\RMSF=1.103e-06\Dipole=0.1041313,1.6292159,1.7894883\
Quadrupole=9.7338499,-3.2669776,-6.4668723,5.3585462,0.3578541,-5.6613364\PG=C01
[X(C21H18O3S1)]\\@

Section 7: *List of references*

[1] Klepp, J.; Dillon, W.; Lin, Y.; P. Feng, P.; Greatrex, B. W. Preparation of (-)-Levoglucosenone from Cellulose Using Sulfuric Acid in Polyethylene Glycol. *Org. Synth.* **2020**, *97*, 38–53. DOI: 10.15227/orgsyn.097.0038.

[2] Mlostoń, G.; Grzelak, P.; Heimgartner, H.; Hetero-Diels-Alder reactions of hetaryl-thiochalcones with acetylenic dienophiles. *J. Sulfur Chem.*, **2017**, *38*, 1−10. DOI: 10.1080/17415993.2016.1230857).

[3] Mlostoń, G.; Urbaniak, K.; Jasiński, M.; Würthwein, E.-U.; Heimgartner, H.; Zimmer, R.; Reissig, H.-U. The (4+2)-cycloaddition of α-nitrosoalkenes with thiochalcones as a prototype of periselective hetero-Diels-Alder reactions – Experimental and computational studies. *Chem. Eur. J.*, **2020**, *26*, 237–248. DOI: 10.1002/chem.201903385.

[4] CrysAlisPRO software system, Oxford Diffraction/Agilent Technologies UK Ltd, Yarnton, England, 2015.

[5] Dolomanov, O.V.; Bourhis, L.J.; Gildea, R.J.; Howard, J.A.K.; Puschmann, H. OLEX2: A Complete Structure Solution, Refinement and Analysis Program. *J. Appl. Crystallogr.* **2009**, *42*, 339–341. DOI: 10.1107/S0021889808042726.

[6] Sheldrick, G.M. SHELXT - Integrated space-group and crystal-structure determination. *Acta Cryst. Sect. A: Foundations and Advances* **2015**, *71*, 3–8. DOI: 10.1107/S2053273314026370.

[7] Sheldrick, G. M. Crystal structure refinement with SHELXL. *Acta Crystallogr. Sect. C: Struct. Chem.* **2015**, *71*, 3–8. DOI: 10.1107/S2053229614024218.

[8] Spek, A. L. Structure validation in chemical crystallography. *Acta Crystallogr. Sect. D: Biol. Crystallogr.* **2009**, *65*, 148–155. DOI: 10.1107/S090744490804362X.

[9] C. R. Groom, I. J. Bruno, M. P. Lightfoot and S. C. Ward, The Cambridge Structural Database. *Acta Cryst*. **2016**. *B72*, 171–179. DOI: 10.1107/S2052520616003954.

[10] Gaussian 16, Revision B.01: M. J. Frisch, G. W. Trucks, H. B. Schlegel, G. E. Scuseria, M. A. Robb, J. R. Cheeseman, G. Scalmani, V. Barone, G. A. Petersson, H. Nakatsuji, X. Li, M. Caricato, A. V. Marenich, J. Bloino, B. G. Janesko, R. Gomperts, B. Mennucci, H. P. Hratchian, J. V. Ortiz, A. F. Izmaylov, J. L. Sonnenberg, D. Williams-Young, F. Ding, F. Lipparini, F. Egidi, J. Goings, B. Peng, A. Petrone, T. Henderson, D. Ranasinghe, V. G. Zakrzewski, J. Gao, N. Rega, G. Zheng, W. Liang, M. Hada, M. Ehara, K. Toyota, R. Fukuda, J. Hasegawa, M. Ishida, T. Nakajima, Y. Honda, O. Kitao, H. Nakai, T. Vreven, K. Throssell, J. A. Montgomery, Jr., J. E. Peralta, F. Ogliaro, M. J. Bearpark, J. J. Heyd, E. N. Brothers, K. N. Kudin, V. N. Staroverov, T. A. Keith, R. Kobayashi, J. Normand, K. Raghavachari, A. P. Rendell, J. C. Burant, S. S. Iyengar, J. Tomasi, M. Cossi, J. M. Millam, M. Klene, C. Adamo, R. Cammi, J. W. Ochterski, R. L. Martin, K. Morokuma, O. Farkas, J. B. Foresman, and D. J. Fox, Gaussian, Inc., Wallingford CT, 2016.

[11] J. P. Perdew, K. Burke, M. Ernzerhof, *Phys. Rev. Lett.* **1996**, *77*, 3865–3868. DOI: 10.1103/PhysRevLett.77.3865

[12] J. P. Perdew, K. Burke, M. Ernzerhof, *Phys. Rev. Lett.* **1997**, *78,* 1396.
DOI: 10.1103/PhysRevLett.78.1396

[13] C. Adamo V. Barone, *J. Chem. Phys.* **1999**, *110*, 6158–6169. DOI: 10.1063/1.478522.

[14] M. Ernzerhof, G. E. Scuseria, *J. Chem. Phys.* **1999**, *110*, 5029–5036. DOI: 10.1063/1.478401

[15] R. Weigend, R. Ahlrichs, *Phys. Chem. Chem. Phys.* **2005**, *7*, 3297–3305. DOI: 10.1039/B508541A

[16] S. Grimme, S. Ehrlich, L. Goerigk, *J. Comput. Chem.* **2011**, *32*, 1456–1465. DOI: 10.1002/jcc.21759

[17] S. Grimme, A. Hansen, J. G. Brandenburg, C. Bannwarth, *Chem. Rev.* **2016**, *116*, 5105–5154. DOI: 10.1021/acs.chemrev.5b00533

[18] J. Tomasi, B. Mennucci, R. Cammi, *Chem. Rev.* **2005**, *105*, 2999–3093. DOI: 10.1021/cr9904009.
